# Generation of Quaternary Carbons in Cycloalkanones and Lactones with Arynes through a Domino Process

**DOI:** 10.1021/acs.joc.4c02257

**Published:** 2024-11-30

**Authors:** Jih Ru Hwu, Khagendra Prasad Bohara, Mohit Kapoor, Animesh Roy, Shu-Yu Lin, Chun-Cheng Lin, Kuo-Chu Hwang, Wen-Chieh Huang, Shwu-Chen Tsay

**Affiliations:** † Department of Chemistry & Frontier Research Center on Fundamental and Applied Sciences of Matters, National Tsing Hua University, Hsinchu 300, Taiwan; ‡ Institute of Biotechnology and Pharmaceutical Research, National Health Research Institutes, Zhunan, Miaoli County 350401, Taiwan

## Abstract

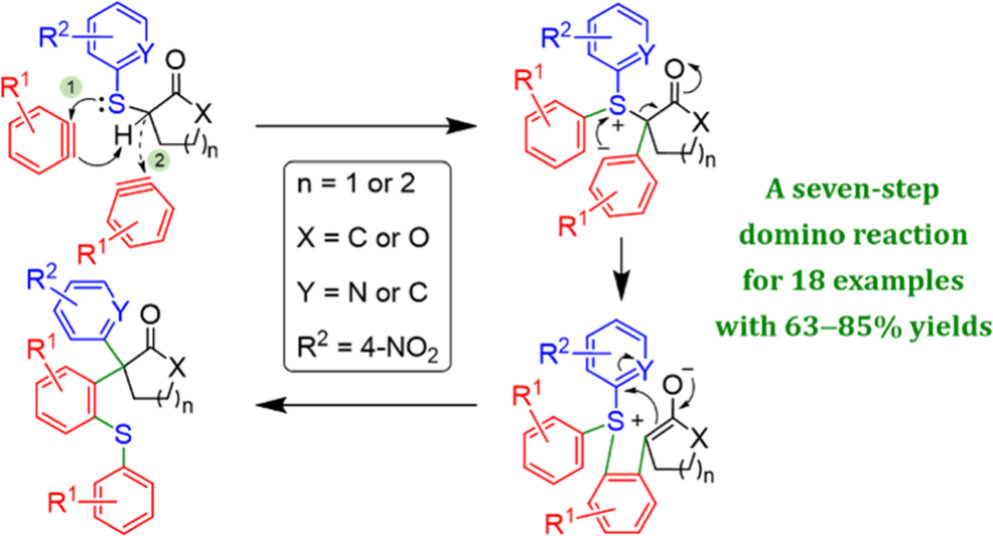

A synthetic method was developed for the generation of a quaternary carbon center in carbonyl compounds. This innovative process involved the reaction of α-thiolate lactones and cycloalkanones with two equivalents of arynes in acetonitrile to give α,α-diarylated products in 63–85% yields at 25 °C. The reaction unfolds through an unconventional domino process, encompassing sequential 1,2-elimination, 1,2-nucleophilic addition, 1,4-proton transfer, the second 1,2-nucleophilic addition, interrupted Pummerer rearrangement, intramolecular spirocyclization, and sulfonium ring-opening. The potential of this “single-flask” reaction was systematically investigated and found well-suited to generate diarylated carbonyl compounds, incorporating naphthalene, pyridine, quinoline, or isoquinoline rings adorned with various substituents.

## Introduction

The formation of a quaternary carbon (4°-C) in molecules poses a challenge in synthetic chemistry. It is due to the difficulties associated with creating steric congestion and the resultant repulsion among all four substituents. Various methods have been developed to address this challenge, including cycloaddition, dearomative alkylation/arylation, Michael addition, polyene cycloaddition, and others.^1,2^ Representative examples include the palladium-catalyzed reaction of an enone with aryl bromide to yield α-aryl-α-alkylcyclopentanones,^3^ the nickel-catalyzed α-arylation of cyclic ketones with aryl halides in the presence of dipyridyldiphosphine ligand to produce a benzophenone,^4,5^ the α-arylation of benzophenones with aryl triflates by use of difluorphos complexes of palladium and nickel,^6^ the arylation of carbonyl compounds with sulfoxides in the presence of trifluoroacetic anhydride,^7^ and so forth. Notably, a 4°-C in lactones can be generated through the Ni/BINAP-catalyzed α-arylation of α-substituted γ-butyrolactones with aryl halides.^8^ Another method involves the CuI-catalyzed coupling reaction between β-keto esters and aryl iodides.^9^ The majority of these methods make use of metal catalysts and find applications in the total syntheses of natural products, including alkaloids and terpenoids.^1,2,10^

The α-arylation of carbonyl compounds^2,11^ holds significant importance, particularly as molecules containing an α-arylated carbonyl moiety with a 4°-C often exhibit noteworthy biological activities.^12^ For instance, Caramiphen serves as a potential antidote against organophosphate poisoning.^13^ Indacrinone is a diuretic drug candidate.^14^ Methadone is well-known for its application in the treatment of neuropathic pain.^15^ Pethidine is widely used for managing labor pain.^16^ Proadifen acts as a cytochrome P450 monoxygenase inhibitor with implications for cancer cell proliferation.^17^ The Sigma-1 receptor agonist PRE-084 is recognized for providing potent neuroprotection.^18^

In the context of synthetic methodology, highly reactive arynes, characterized by distinct electrophilicity, are employed in intramolecular aryne–ene reactions, intermolecular sp^2^ C–H bond arylation, and insertion into a C_sp_–O_sp_^3^σ bond.^19−21^ These arynes also serve as synthetic building blocks for the arylation of β-keto esters^22^ and are utilized in α-arylation reactions with secondary β-keto amides^23^ as well as malonamide esters.^24^ A recent development involves an electrochemical approach for the oxidative generation of benzynes, which react with β-keto esters^25^ in an asymmetric manner to afford products with a 4°-C.

**Scheme 1:**
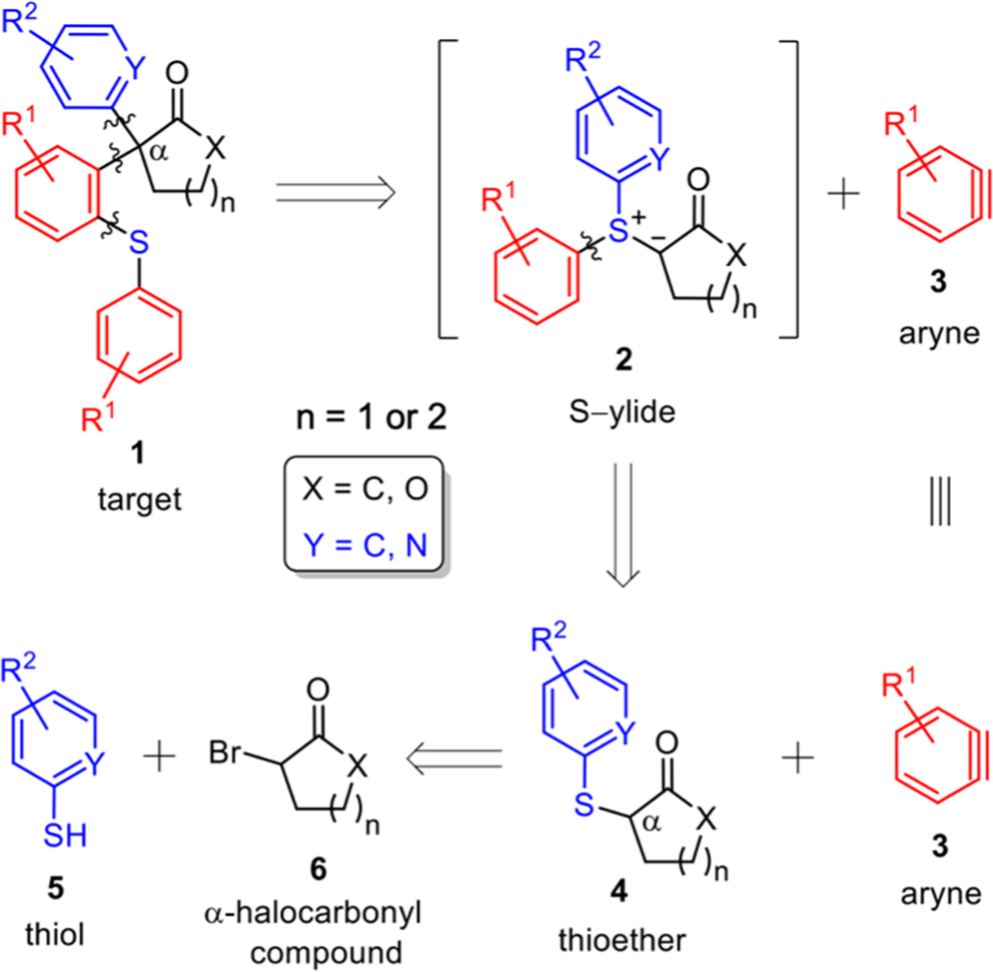
Retrosynthetic Strategy for the Target Carbonyl Compounds **1**, Featuring a Quaternary Carbon Center, by Use of Two Equivalents of Arynes **3**

Our research group has been actively involved in developing domino reactions for chemical syntheses, with a focus on leveraging arynes to access various compound classes.^26^ These encompass alkenes with optical activity, α-amino acids, chroman-2-ones, deoxy- and iminosugars, imidazolidines, phenanthrenes, pyrroles, and pyrrolidines. In pursuit of advancing aryne-induced reactions, we established a method for generating triaryl (including heteroarene) carbonyl compounds **1** featuring a 4°-C. The retrosynthetic analysis of this process is depicted in Scheme 1. The reaction involved the independent reaction of intermediates sulfur ylides **2** and thioethers **4** with arynes **3** in sequence in situ. Thioethers **4** can be readily prepared through the coupling of thiobenzenes or 2-mercaptopyridines **5** (where Y = C or N) with bromocycloalkanones or -lactones **6** (where X = C or O; *n* = 1 or 2).^27^ This innovative (**4** + **3**) + **3** → **1** domino process integrated multiple steps within a single flask and resulted in the impressive generation of 4°-C in carbonyl compounds **1** with high yields. This approach represents a noteworthy contribution to the field of synthetic chemistry.

## Results

**Scheme 2:**
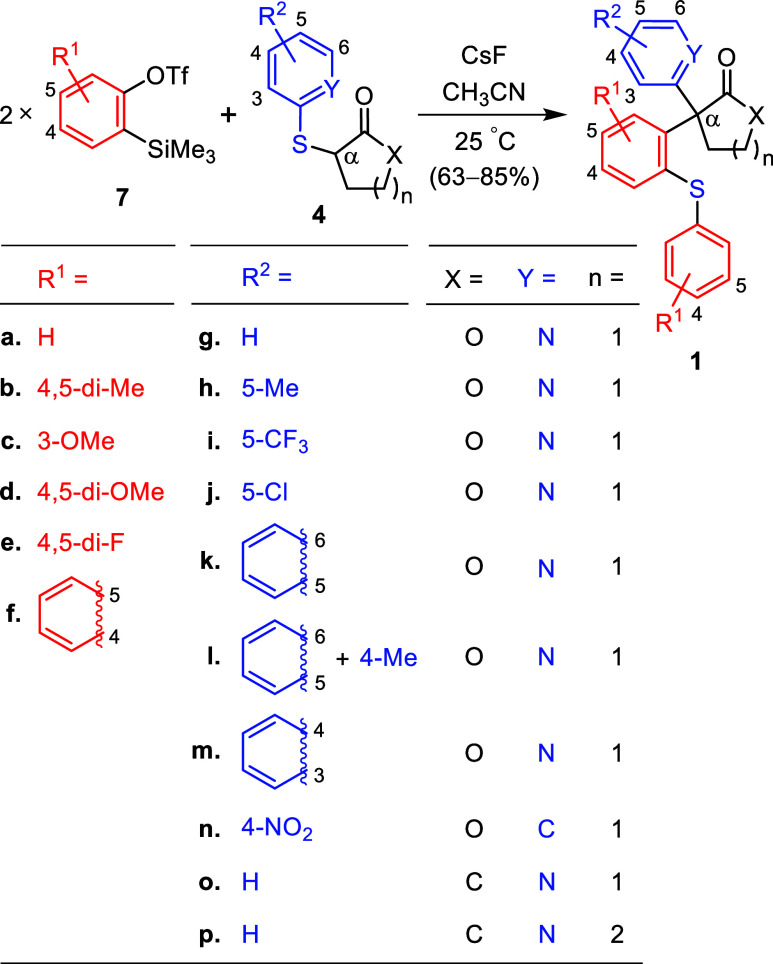
Synthesis of α,α-Diarylated Lactones and Cycloalkanones **1** from Silyl Triflates **7** and Thioethers **4**

In order to implement the reaction outlined in Scheme 2, we treated the aryne precursor, (trimethylsilyl)aryl triflate **7a** (2.0 equiv), with cesium fluoride (4.0 equiv) and α-thiolate lactone **4g** (1.0 equiv) in acetonitrile. After a 16-h reaction period at 25 °C and normal workup, a viscous yellow oil was obtained and then purified through column chromatography to give solids. The IR spectrum of the product exhibited a prominent absorption band at 1768 cm^–1^, indicative of the C=O stretching vibration characteristic of a γ-lactone. Simultaneously, its carbon nuclear magnetic resonance (^13^C{^1^H} NMR) spectrum displayed a weak peak at 60.4 ppm, corresponding to a 4°-C. Nevertheless, this result deviated from our calculated value of 70.5 ppm for a lactone molecule with a quaternary C(COO−)(S–Ar)(C-alkyl)(C-Ph) center. This discrepancy of approximately 10 ppm in the ^13^C{^1^H} NMR spectrum prompted reconsideration of the product’s structure, suggesting a structure that did not arise from the straightforward attachment of aryne **3** at the α position of lactone **4g** bearing a −SAr moiety.

**Figure 1:**
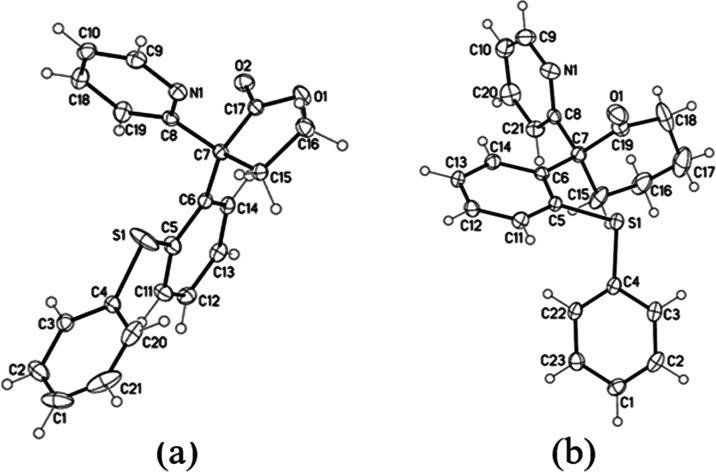
ORTEP diagrams of (a) α,α-diarylated lactone **1ag**, and (b) α,α-diarylated cyclohexanone **1ap**, as determined by X-ray analysis (ellipsoid contour probability 50%).

To elucidate the actual structure of the product, we recrystallized the obtained solids in methanol to give the pure triclinic crystals with mp 138.2–139.6 °C. Single-crystal X-ray diffraction analysis revealed that this lactone belonged to the space group *P*1, with crystal parameters *a* = 7.9281(5) Å, *b* = 9.2566(6) Å, *c* = 11.5548(7) Å, α = 91.7930(10)°, β = 99.0670(10)°, and γ = 98.9810(10)°. Its ORTEP diagram is depicted in Figure 1a.

Further characterization included the measurement of its exact mass as 348.1060 for (M + H)^+^, closely aligned with the theoretical value of 348.1058 for (C_21_H_17_NO_2_S + H)^+^. The proton nuclear magnetic resonance (^1^H NMR) spectrum displayed two multiplets at 4.20–4.13 and 2.80–2.73 ppm for the CH_2_CCO protons, while two additional multiplets occurred at 4.55–4.50 and 4.37–4.31 ppm for the CH_2_OC=O protons on the lactone ring. The carbonyl carbon exhibited a peak at 177.7 ppm in its ^13^C{^1^H} NMR spectrum. Peaks at 66.6 ppm for the CH_2_OCO carbon and 34.5 ppm for the CH_2_CCO carbon were also observed. These findings provided compelling evidence to support the feasibility of our proposed reaction in Scheme 2 and confirmed that the product obtained was indeed the α,α-diarylated lactone **1ag**. Furthermore, a closely related compound, α,α-diarylated cyclohexanone **1ap** (cf. lactone **1ag**), was synthesized through a similar reaction in 78% yield. Monoclinic crystals of this compound belonged to the space group *P*21/*c*, with crystal parameters *a* = 16.4567(4) Å, *b* = 7.9050(2) Å, *c* = 14.9464(3) Å, α = 90°, β = 111.8510(10)°, and γ = 90°. The corresponding ORTEP diagram is depicted in Figure 1b.

For exploration of the reaction scope, various of aryl triflates **7** and thiolate carbonyl compounds **4** as listed in Table 1 were employed. The silylaryl triflates **7** featured benzene nuclei with substituents such as Me, −OMe, and F groups (i.e., b–e). Additionally, the benzene ring was replaced by a naphthalene ring (i.e., f). Thiolate carbonyl compounds **4**(27) included an example with a benzene ring (i.e., Y = C) bearing an electron-withdrawing −NO_2_ group (i.e., n). Furthermore, the aromatic ring in compounds **4** could be varied to include pyridine (i.e., g–j, o, and p with Y = N), quinoline (i.e., k and l with Y = N), or isoquinoline ring (i.e., m with Y = N). These rings were attached with various substituents such as Me, −CF_3_, and Cl groups (i.e., h, i, and j, respectively). The aliphatic ring in **4** could be a cyclopentane (i.e., X = C, *n* = 1), cyclohexane (i.e., X = C, n = 2), and γ-lactone (i.e., X = O, *n* = 1).

**Table 1:**
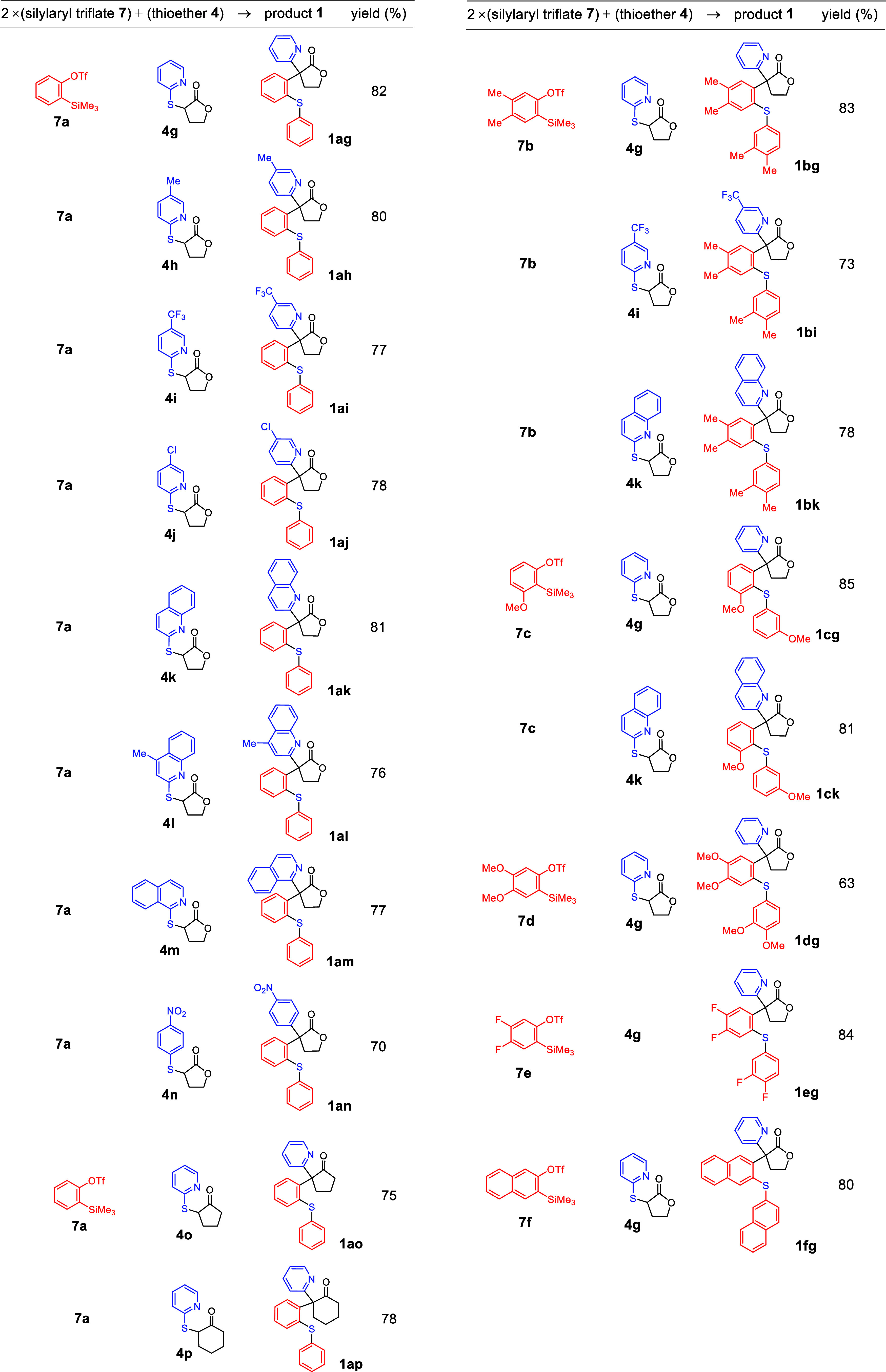
Structures of Reactants **7** and **4**, α,α-Diarylated Carbonyl Products **1**, as well as their Isolated Yields

The application of the reaction conditions outlined above for the reaction shown in Scheme 2 led to the synthesis of 18 new compounds featuring with a 4°-C (see Table 1). The structure identification of all new compounds was on the basis of their high-resolution mass, ^1^H NMR, ^13^C NMR, and infrared spectroscopy (IR) spectroscopy. Detailed data for these compounds are provided in the Supporting Information.

These compounds were obtained in 63–85% yields with purity >98.6% after purification through column chromatography. In a control experiment, a solution containing silyl triflate **7a** (1.0 equiv) and cesium fluoride (2.0 equiv) was treated with only one equivalent of α-thiolate lactone **4g**. Under the identical reaction conditions mentioned previously, compound **1ag** was generated as the exclusive product in 38% yield.

## Discussion

**Scheme 3:**
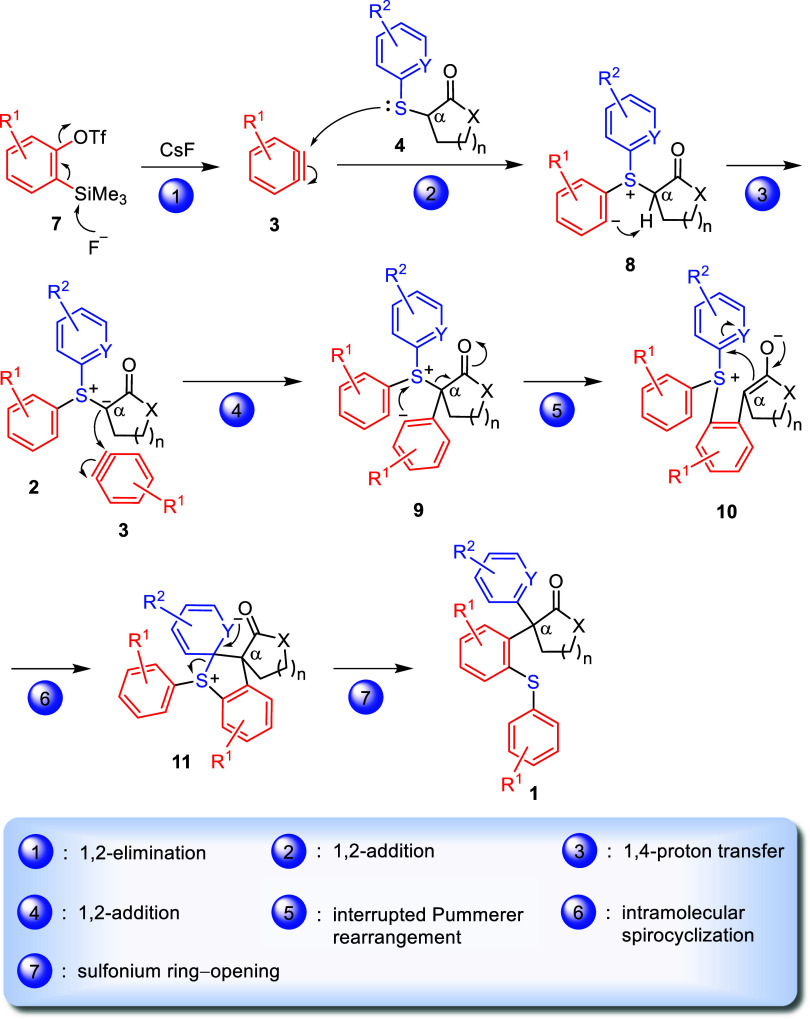
Proposed Mechanism of an Aryne-Induced Domino Process to Produce α,α-Diarylated Carbonyl Compounds Featuring with a Quaternary Carbon

An intriguing feature of Scheme 2 is that the α 4°-C generated in the carbonyl products **1** is no longer directly attached to a sulfur atom, as observed in the starting materials **4**. This outcome can be rationalized through a seven-step mechanism outlined in Scheme 3. After a 1,2-elimination occurs to the silyl triflates **7**, the resultant arynes **3** are attacked by thioethers **4** to generate the sulfonium betaines **8** through a 1,2-nucleophilic addition.^28^ Then an intramolecular 1,4-proton transfer^29^ takes place in betaines **8** through a five-membered ring transition state to give the corresponding ylides **2**. In situ, the second equivalent of arynes **3** arylates ylides **2** to give the adducts **9** through the second 1,2-nucleophilic addition. At this step, the first arylation of the α-carbon of carbonyl compounds **4** is accomplished.

The sp^2^ carbanion in adducts **9** may attack the tricoordinate sulfonium center^30^ to give the betaines **10**, which contain an enolate moiety, through an interrupted Pummerer rearrangement.^31^ Subsequently, an alkylation takes place between the enolate moiety and the electron-withdrawing aromatic ring in **10** to form the intermediates **11** with a five-membered sulfonium ring. This intramolecular spirocyclization step sets the stage for the second arylation of the α-carbon of carbonyl compounds **4**. Finally, a ring-opening occurs in the cyclic sulfonium moiety, concomitant with simultaneous rearomatization to give α,α-diarylates **1** with a 4°-C as the final products.

A crucial determinant for the reaction to be successful was the incorporation of a pyridine ring with the nitrogen atom (i.e., Y) at the ortho position in thioethers **4**. This electron-withdrawing atom played a vital role in facilitating the conversion of betaines **10** → **11** through a spirocyclization and enabling the generation of sulfonium intermediates **11**, as shown in Scheme 3.

When the pyridine moiety was replaced by a benzene ring, the introduction of an electron-withdrawing −NO_2_ group at the para position also proved effective in promoting this process. Consequently, the starting material lactone **4n** underwent arylation to give α,α-diarylated lactone **1an** with success in 70% yield.

During optimization of conditions for the reaction shown in Scheme 2, various combinations of fluoride reagents, solvents, additives, temperatures, and reaction time were applied to the reactants **7a** and **4g**, as detailed in Table S1 in the Supporting Information. For the generation of α,α-diarylated carbonyl compounds **1**, the use of 2.0 equiv (instead of one equivalent) of the silylaryl triflates **7** was essential. Among the fluoride reagents tested for the conversion of silylaryl triflates **7** to the corresponding arynes, CsF proved to be the most effective and gave the reaction yields as high as 85% (for **1cg**). In contrast, substitution of CsF with KF or *n*-Bu_4_NF resulted in significantly lower yields in 27% only or less. Attempts to employ alternative methods for aryne generation through 1,2-elimination,^32,33^ such as use of strong bases or at higher temperatures, were unsuccessful. These methods also included the applications of the mixtures of NaNH_2_ with aryl halides,^32^*n*-BuLi with 1,2-dibromobenzene,^32^ Mg with 1,2-bromofluorobenzene,^33^ and pyrolysis of arenediazonium 2-carboxylates.^32^

Among various solvents investigated, including acetonitrile, 2-MeTHF, THF, and toluene, acetonitrile emerged as the optimal choice for the reaction. The use of acetonitrile obviated the need for the addition of 18-crown-6 as a ligand for the Cs^+^ species. Additionally, the dual arylation processes progressed at 25 °C. Elevation of the reaction temperature was deemed unnecessary and counterproductive, which resulted in lower yields of the desired products **1**.

The domino reaction illustrated in Scheme 2 possesses the following six distinctive characteristics: (1) This innovative method was designed to maximize the incorporation of most elements from the starting materials into the final products. (2) Performance of the reaction at 25 °C under normal pressure ensures energy efficiency throughout the synthetic process. (3) The need for derivatization, involving the use of blocking groups, protection/deprotection, and temporary modification of the arylation process, is eliminated. (4) The developed method does not involve metal catalysts and thus minimizes potential harm to sustainable development and living organisms. This significantly reduces the possibility of environmental and toxicity concerns.^34^ (5) The absence of byproducts and hazardous substances in the environment leads to a substantial reduction in waste generation. (6) The need for precautions related to chemical accidents, including releases, explosions, and fires, is minimized, contributing to a safer operational environment.

## Conclusions

A synthetic method was developed for the generation of 4°-C in cycloalkanones and lactones under mild conditions by use of two equivalents of arynes to react with α-thiolate carbonyl compounds. This process underwent double α-arylations to afford the products in 63–85% yields in a single flask. The entire domino process encompasses seven sequential steps. They are 1,2-elimination, 1,2-nucleophilic addition, 1,4-proton transfer, the second 1,2-nucleophilic addition, interrupted Pummerer rearrangement, intramolecular spirocyclization, and sulfonium ring-opening. Notably, the isolation of intermediates is unnecessary. The application of this method in synthetic chemistry aligns with most requirements of the principles of green chemistry.^35^

## Data Availability

The data underlying this study are available in the published article and its online Supporting Information.

## Supporting Information Available

Materials; experimental details; spectroscopic data of all new compounds; references; and spectra (PDF)
