# Supplementary material for: Generation of Quaternary Carbons in Cycloalkanones and Lactones with Arynes through a Domino Process
Source: J Org Chem. 2024 Nov 30;89(24):18393–9. doi: 10.1021/acs.joc.4c02257 (PMC11667720; doi:10.1021/acs.joc.4c02257)
Supplement: Supplementary file 1 — jo4c02257_si_001.pdf [file jo4c02257_si_001.pdf]

Supporting Information

Generation of Quaternary Carbons in Cycloalkanones and Lactones with Arynes through a Domino Process

Jih Ru Hwu,[a,]* Khagendra Prasad Bohara,[a] Mohit Kapoor,[a] Animesh Roy,[a] Shu-Yu Lin,[b] Chun-Cheng Lin,[a] Kuo-Chu Hwang,[a] Wen-Chieh Huang,[a,b] and Shwu-Chen Tsay[a]

[a]Department of Chemistry & Frontier Research Center on Fundamental and Applied Sciences of Matters, National Tsing Hua University, Hsinchu 300, Taiwan

[b]Institute of Biotechnology and Pharmaceutical Research, National Health Research Institutes, Miaoli County 350401, Taiwan

E-mail: jrhwu@mx.nthu.edu.tw

Contents

General Information S2

Standard Procedure 1 for the Synthesis of Thioethers S3

Standard Procedure 2 for the Synthesis of α,α-Diarylated Cycloalkanones or Lactones S3

Experimental Data of Thioethers S4

Experimental Data of α,α-Diarylated Cycloalkanones or Lactones S10

Optimization of Reaction Conditions S28

References S29

X-ray Crystallographic Details S30

X-ray Crystal Data for Compound 1ag S30

X-ray Crystal Data for Compound 1ap S32

X-ray Crystal Data for Compound 1am S34

^{1}H NMR, ^{13}C{^{1}H} NMR, and IR Spectra of New Compounds S36

General Information

All reactions were carried out in oven-dried glassware (120 °C) under an atmosphere of nitrogen unless as indicated otherwise. Acetonitrile, dichloromethane, ethyl acetate, hexanes, and toluene from Mallinckrodt Chemical Co. were dried and distilled from CaH_2. Tetrahydrofuran (THF) from Mallinckrodt Chemical Co. was dried by distillation from sodium and benzophenone under an atmosphere of nitrogen. 2-Methyltetrahydrofuran (2-MeTHF) was purchased from Sigma-Aldrich. The reagents purchased from Alfa Aesar included α-bromo-γ-butyrolactone, 4-methyl-2-quinolinethiol, 4-nitrothiophenol, and potassium carbonate (K_2CO_3). The reagents purchased from Sigma-Aldrich included 2-bromocyclohexanone, 2-bromocyclopentanone, 5-chloropyridine-2-thiol, 2-mercaptopyridine, 2-quinolinethiol, and 5-(trifluoromethyl)pyridine-2-thiol. The reagents purchased from Combi-Blocks included isoquinoline-1-thiol and 2-mercapto-5-methylpyridine. Cesium fluoride (CsF), 18-crown-6, potassium fluoride (KF), and tetra-*n*-butylammonium fluoride (TBAF) were purchased from Acros. The reagents purchased from Tokyo Chemical Industry Co. included 4,5-dimethoxy-2-(trimethylsilyl)phenyl trifluoromethanesulfonate, 3-methoxy-2-(trimethylsilyl)phenyl trifluoromethanesulfonate, 3-(trimethylsilyl)-2-naphthyl trifluoromethanesulfonate, and 2-(trimethylsilyl)phenyl trifluoromethanesulfonate. 4,5-Difluoro-2-(trimethylsilyl)phenyl trifluoromethanesulfonate[1] and 4,5-dimethyl-2-(trimethylsilyl)phenyl trifluoromethanesulfonate[2] were prepared according to the reported procedures.

Analytical thin-layer chromatography (TLC) was performed on precoated plates (silica gel 60 F-254). Purification by gravity column chromatography was carried out by use of Silicycle ultrapure silica gel (particle size 40–63 μm, 230–400 mesh).

Infrared (IR) spectra were recorded on a Fourier transform infrared (FT-IR) spectrometer. Absorption intensities are recorded by the following abbreviations: s, strong; m, medium; and w, weak. Proton NMR spectra were obtained on a 400 MHz spectrometer by use of

chloroform-*d* ($CDCl_3$) as the solvent. Proton NMR chemical shifts were referenced to the residual protonated solvent (δ 7.24 ppm for chloroform). Carbon-13 NMR spectra were obtained on a 100 MHz spectrometer by use of chloroform-*d* ($CDCl_3$) as the solvent. Carbon-13 chemical shifts were referenced to the center of the $CDCl_3$ triplet (δ 77.0 ppm). Multiplicities are recorded by the following abbreviations: s, singlet; d, doublet; dd, doublet of doublet; t, triplet; q, quartet; m, multiplet; and *J*, coupling constant (hertz). High-resolution mass spectra (HRMS) were measured on an instrument by use of a time-of-flight (TOF) mass analyzer with electrospray ionization (ESI).

Standard Procedure 1 for the Synthesis of Thioethers. To a stirred solution of thiol **5** (1.0 equiv) in THF (10 mL) at room temperature was added K_2CO_3 (1.5 equiv) followed by α-bromocarbonyl compound **6** (1.2 equiv) under a nitrogen atmosphere. After the reaction mixture was refluxed at 70 °C by the use of an oil bath for 3.0–4.0 h, it was cooled down to room temperature. The solvent was evaporated under reduced pressure, the residue was dissolved in water (20 mL) and then extracted with EtOAc (3 × 20 mL). The combined organic layers were dried over $MgSO_4$ (s), filtered, and concentrated under reduced pressure to afford the residue. The residue was then purified by use of column chromatography on silica gel with EtOAc in hexanes as eluent to give the desired thioether **4**.[3]

Standard Procedure 2 for the Synthesis of α,α-Diarylated Cycloalkanones or Lactones. To a stirred solution of 2-silylphenyl triflate **7** (2.0 equiv) and thioether **4** (1.0 equiv) in dry CH_3CN (2.0–2.5 mL) was added CsF (4.0–4.1 equiv) at room temperature under argon atmosphere. After the reaction mixture was stirred at 25 °C for 16–18 h, it was concentrated under reduced pressure and the residue was dissolved in EtOAc (20 mL). The organic layer was washed with water (3 × 20 mL) and brine (20 mL); then dried over $MgSO_4$ (s), filtered, and concentrated under reduced pressure to afford the residue. The residue was then purified

by use of column chromatography on silica gel with EtOAc in hexanes as the eluent to afford the desired α,α-diarylated cycloalkanone or lactone **1**.

Experimental Data of Thioethers

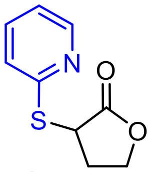

4g

3-(Pyridin-2-ylthio)dihydrofuran-2(3*H*)-one (4g). The standard procedure 1 was followed by use of 2-mercaptopyridine (**5a**, 128 mg, 1.15 mmol, 1.0 equiv), α-bromo-γ-butyrolactone (**6a**, 228 mg, 1.38 mmol, 1.2 equiv), and K_2CO_3 (239 mg, 1.73 mmol, 1.5 equiv) in THF (10 mL). After the reaction mixture was refluxed for 3.0 h and then worked up, the residue was purified by use of column chromatography (20% EtOAc in hexanes as the eluent) to give the desired thioether **4g** (201 mg, 1.03 mmol) in 89% yield as a colorless oil: TLC R_f 0.47 (40% EtOAc in hexanes as the eluent); ^{1}H NMR ($CDCl_3$, 400 MHz) δ 8.36 (d, J = 4.0 Hz, 1 H, ArH), 7.49 (t, J = 7.2 Hz, 1 H, ArH), 7.19 (d, J = 8.0 Hz, 1 H, ArH), 7.00 (t, J = 6.0 Hz, 1 H, ArH), 4.55–4.45 (m, 2 H, OCH_2), 4.37–4.31 (m, 1 H, CHCO), 2.83–2.75 (m, 1 H, ½ × C*H*$_2$CH), 2.49–2.39 (m, 1 H, ½ × C*H*$_2$CH); ^{13}C{^{1}H} NMR ($CDCl_3$, 100 MHz) δ 175.3 (C=O), 155.5, 149.2, 136.3, 122.3, 120.1, 66.5 (OCH_2), 40.4 (*C*HCO), 30.0 (*C*H$_2$CH); IR (neat) 2912 (w, C–H stretch), 1770 (s, C=O), 1578 (m), 1373 (m), 1283 (w), 1162 (m), 1022 (m), 761 (m) cm^{-1}; HRMS (ESI-TOF) m/z: $[M + H]^+$ Calcd for $C_9H_{10}NO_2S$ 196.0432; Found 196.0435.

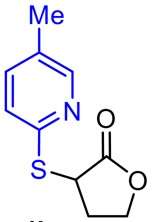

4h

3-[(5-Methylpyridin-2-yl)thio]dihydrofuran-2(3*H*)-one (4h). The standard procedure 1 was followed by use of 2-mercapto-5-methylpyridine (**5b**, 142 mg, 1.13 mmol, 1.0 equiv), α-

bromo-γ-butyrolactone (**6a**, 225 mg, 1.36 mmol, 1.2 equiv), and K_2CO_3 (236 mg, 1.71 mmol, 1.5 equiv) in THF (10 mL). After the reaction mixture was refluxed for 4.0 h and then worked up, the residue was purified by use of column chromatography (25% EtOAc in hexanes as the eluent) to give the desired thioether **4h** (199 mg, 0.951 mmol) in 84% yield as a colorless oil: TLC R_f 0.37 (40% EtOAc in hexanes as the eluent); ^{1}H NMR ($CDCl_3$, 400 MHz) δ 8.21 (s, 1 H, ArH), 7.33 (d, J = 7.6 Hz, 1 H, ArH), 7.12 (d, J = 8.0 Hz, 1 H, ArH), 4.52–4.44 (m, 2 H, OCH_2), 4.37–4.31 (m, 1 H, CHCO), 2.83–2.74 (m, 1 H, ½ × C*H*$_2$CH), 2.48–2.39 (m, 1 H, ½ × C*H*$_2$CH), 2.24 (s, 3 H, CH_3); ^{13}C{^{1}H} NMR ($CDCl_3$, 100 MHz) δ 175.4 (C=O), 152.0, 149.5, 137.3, 129.9, 122.1, 66.5 (OCH_2), 40.5 (*C*HCO), 30.1 (*C*H$_2$CH), 17.8 (CH_3); IR (neat) 2919 (w, C–H stretch), 1768 (s, C=O), 1591 (w), 1460 (m), 1368 (m), 1156 (m), 1022 (m), 818 (w) cm^{-1}; HRMS (ESI-TOF) m/z: $[M + H]^+$ Calcd for $C_{10}H_{12}NO_2S$ 210.0588; Found 210.0585.

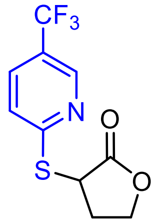

4i

3-([5-(Trifluoromethyl)pyridin-2-yl]thio)dihydrofuran-2(3*H*)-one (4i). The standard procedure 1 was followed by use of 5-(trifluoromethyl)pyridine-2-thiol (**5c**, 102 mg, 0.569 mmol, 1.0 equiv), α-bromo-γ-butyrolactone (**6a**, 113 mg, 0.683 mmol, 1.2 equiv), and K_2CO_3 (118 mg, 0.854 mmol, 1.5 equiv) in THF (10 mL). After the reaction mixture was refluxed for 4.0 h and then worked up, the residue was purified by use of column chromatography (10% EtOAc in hexanes as the eluent) to give the desired thioether **4i** (117 mg, 0.444 mmol) in 78% yield as a colorless liquid: TLC R_f 0.51 (20% EtOAc in hexanes as the eluent); ^{1}H NMR ($CDCl_3$, 400 MHz) δ 8.63 (s, 1 H, ArH), 7.71 (d, J = 8.4 Hz, 1 H, ArH), 7.31 (d, J = 8.4 Hz, 1 H, ArH), 4.59–4.49 (m, 2 H, OCH_2), 4.40–4.34 (m, 1 H, CHCO), 2.87–2.79 (m, 1 H, ½ × C*H*$_2$CH), 2.52–2.42 (m, 1 H, ½ × C*H*$_2$CH); ^{13}C{^{1}H} NMR ($CDCl_3$, 100 MHz) δ 174.5 (C=O),

160.7, 146.1 (q, J = 4.2 Hz), 133.1 (q, J = 3.2 Hz), 123.5 (q, J = 270.2 Hz), 123.1 (q, J = 33.1 Hz), 121.7, 66.5 (OCH_2), 40.6 (*C*HCO), 29.7 (CH_2CH); IR (neat) 2917 (w, C–H stretch), 1771 (s, C=O), 1601 (s), 1473 (w), 1327 (s), 1118 (s), 1010 (m), 833 (w) cm^{-1}; HRMS (ESI-TOF) m/z: $[M + H]^+$ Calcd for $C_{10}H_9F_3NO_2S$ 264.0306; Found 264.0300.

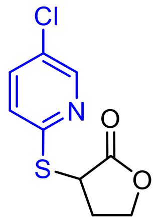

4j

3-[(5-Chloropyridin-2-yl)thio]dihydrofuran-2(3*H*)-one (4j). The standard procedure 1 was followed by use of 5-chloropyridine-2-thiol (**5d**, 166 mg, 1.14 mmol, 1.0 equiv), α-bromo-γ-butyrolactone (**6a**, 226 mg, 1.37 mmol, 1.2 equiv), and K_2CO_3 (237 mg, 1.71 mmol, 1.5 equiv) in THF (10 mL). After the reaction mixture was refluxed for 4.0 h and then worked up, the residue was purified by use of column chromatography (20% EtOAc in hexanes as the eluent) to give the desired thioether **4j** (212 mg, 0.923 mmol) in 81% yield as a colorless liquid: TLC R_f 0.54 (40% EtOAc in hexanes as the eluent); ^1H NMR ($CDCl_3$, 400 MHz) δ 8.33 (s, 1 H, ArH), 7.48 (d, J = 8.0 Hz, 1 H, ArH), 7.15 (d, J = 8.4 Hz, 1 H, ArH), 4.48 (t, J = 9.0 Hz, 2 H, OCH_2), 4.37–4.31 (m, 1 H, CHCO), 2.80–2.78 (m, 1 H, ½ × CH_2CH), 2.48–2.38 (m, 1 H, ½ × CH_2CH); $^{13}C\{^1H\}$ NMR ($CDCl_3$, 100 MHz) δ 174.8 (C=O), 153.9, 147.9, 136.3, 128.7, 122.9, 66.5 (OCH_2), 40.7 (*C*HCO), 29.8 (CH_2CH); IR (neat) 2903 (w, C–H stretch), 1760 (s, C=O), 1567 (m), 1444 (m), 1214 (w), 1117 (m), 950 (w), 827 (m) cm^{-1}; HRMS (ESI-TOF) m/z: $[M + H]^+$ Calcd for $C_9H_9ClNO_2S$ 230.0042; Found 230.0035.

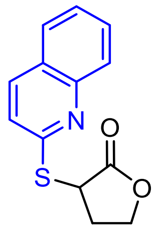

4k

3-(Quinolin-2-ylthio)dihydrofuran-2(3*H*)-one (4k). The standard procedure 1 was followed by use of 2-quinolinethiol (**5e**, 181 mg, 1.12 mmol, 1.0 equiv), α-bromo-γ-butyrolactone (**6a**, 222 mg, 1.34 mmol, 1.2 equiv), and K_2CO_3 (233 mg, 1.68 mmol, 1.5 equiv) in THF (10 mL). After the reaction mixture was refluxed for 3.0 h and then worked up, the residue was purified by use of column chromatography (25% EtOAc in hexanes as the eluent) to give the desired thioether **4k** (228 mg, 0.929 mmol) in 83% yield as a colorless viscous liquid: TLC R_f 0.41 (40% EtOAc in hexanes as the eluent); ^{1}H NMR ($CDCl_3$, 400 MHz) δ 7.92 (d, J = 8.8 Hz, 1 H, ArH), 7.88 (d, J = 8.4 Hz, 1 H, ArH), 7.7 (d, J = 8.0 Hz, 1 H, ArH), 7.63 (t, J = 7.8 Hz, 1 H, ArH), 7.43 (t, J = 7.4 Hz, 1 H, ArH), 7.20 (d, J = 8.4 Hz, 1 H, ArH), 4.66–4.57 (m, 2 H, OCH_2), 4.47–4.41 (m, 1 H, CHCO), 2.93–2.85 (m, 1 H, ½ × C*H*$_2$CH), 2.63–2.53 (m, 1 H, ½ × C*H*$_2$CH); ^{13}C{^{1}H} NMR ($CDCl_3$, 100 MHz) δ 175.3 (C=O), 155.7, 147.8, 136.1, 129.8, 127.7, 127.6, 126.1, 125.7, 120.3, 66.6 (OCH_2), 40.5 (*C*HCO), 29.7 (*C*H_2CH); IR (neat) 2917 (w, C–H stretch), 1759 (s, C=O), 1591 (w), 1374 (w), 1164 (m), 1016 (w), 948 (w), 820 (w) cm^{-1}; HRMS (ESI-TOF) m/z: $[M + H]^+$ Calcd for $C_{13}H_{12}NO_2S$ 246.0588; Found 246.0587.

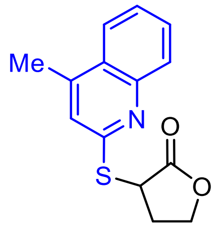

4l

3-[(4-Methylquinolin-2-yl)thio]dihydrofuran-2(3*H*)-one (4l). The standard procedure 1 was followed by use of 4-methyl-2-quinolinethiol (**5f**, 202 mg, 1.15 mmol, 1.0 equiv), α-bromo-γ-butyrolactone (**6a**, 228 mg, 1.38 mmol, 1.2 equiv), and K_2CO_3 (239 mg, 1.73 mmol, 1.5 equiv) in THF (10 mL). After the reaction mixture was refluxed for 3.0 h and then worked up, the residue was purified by use of column chromatography (25% EtOAc in hexanes as the eluent) to give the desired thioether **4l** (242 mg, 0.933 mmol) in 81% yield as a colorless viscous liquid: TLC R_f 0.49 (40% EtOAc in hexanes as the eluent); ^{1}H NMR ($CDCl_3$, 400 MHz) δ 7.88–7.85

(m, 2 H, 2 × ArH), 7.61 (t, *J* = 8.0 Hz, 1 H, ArH), 7.44 (t, *J* = 8.0 Hz, 1 H, ArH), 7.05 (s, 1 H, ArH), 4.64–4.56 (m, 2 H, OCH_2), 4.46–4.39 (m, 1 H, CHCO), 2.91–2.83 (m, 1 H, ½ × C*H*2CH), 2.58–2.53 (m, 4 H, ½ × C*H*2CH + CH_3); $^{13}C\{^1H\}$ NMR ($CDCl_3$, 100 MHz) *δ* 175.5 (C=O), 155.3, 147.6, 144.4, 129.5, 128.1, 126.3, 125.4, 123.8, 120.4, 66.5 (OCH_2), 40.3 (*C*HCO), 29.8 (*C*H2CH), 18.5 (CH_3); IR (neat) 2912 (w, C–H stretch), 1770 (s, C=O), 1595 (m), 1447 (w), 1296 (w), 1147 (m), 1023 (w), 759 (m) cm^{-1}; HRMS (ESI-TOF) m/z: $[M + H]^+$ Calcd for $C_{14}H_{14}NO_2S$ 260.0745; Found 260.0742.

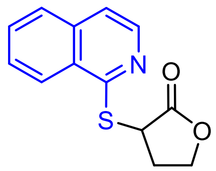

4m

3-(Isoquinolin-1-ylthio)dihydrofuran-2(3*H*)-one (4m). The standard procedure 1 was followed by use of isoquinoline-1-thiol (**5g**, 187 mg, 1.16 mmol, 1.0 equiv), α-bromo-γ-butyrolactone (**6a**, 231 mg, 1.39 mmol, 1.2 equiv), and K_2CO_3 (241 mg, 1.74 mmol, 1.5 equiv) in THF (10 mL). After the reaction mixture was refluxed for 3.0 h and then worked up, the residue was purified by use of column chromatography (20% EtOAc in hexanes as the eluent) to give the desired thioether **4m** (217 mg, 0.884 mmol) in 76% yield as a colorless viscous liquid: TLC R_f 0.46 (40% EtOAc in hexanes as the eluent); ^1H NMR ($CDCl_3$, 400 MHz) *δ* 8.24 (d, *J* = 5.6 Hz, 1 H, ArH), 8.07 (d, *J* = 8.4 Hz, 1 H, ArH), 7.75 (d, *J* = 8.0 Hz, 1 H, ArH), 7.66 (t, *J* = 7.4 Hz, 1 H, ArH), 7.55 (t, *J* = 7.6 Hz, 1 H, ArH), 7.36 (d, *J* = 6.0 Hz, 1 H, ArH), 4.79–4.74 (m, 1 H, ½ × OCH_2), 4.59–4.53 (m, 1 H, ½ × OCH_2), 4.44–4.38 (m, 1 H, CHCO), 2.92–2.84 (m, 1 H, ½ × C*H*2CH), 2.57–2.47 (m, 1 H, ½ × C*H*2CH); $^{13}C\{^1H\}$ NMR ($CDCl_3$, 100 MHz) *δ* 175.4 (C=O), 156.1, 141.3, 135.6, 130.6, 127.3, 127.1, 126.6, 124.1, 118.0, 66.7 (OCH_2), 40.3 (*C*HCO), 30.1 (*C*H2CH); IR (neat) 2911 (w, C–H stretch), 1770 (s, C=O), 1552 (m), 1450 (w), 1306 (m), 1171 (m), 949 (w), 820 (m) cm^{-1}; HRMS (ESI-TOF) m/z: $[M + H]^+$ Calcd for $C_{13}H_{12}NO_2S$ 246.0588; Found 246.0590.

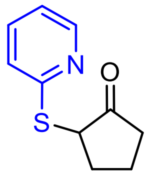

4o

2-(Pyridin-2-ylthio)cyclopentan-1-one (4o). The standard procedure 1 was followed by use of 2-mercaptopyridine (**5a**, 132 mg, 1.18 mmol, 1.0 equiv), 2-bromocyclopentanone (**6b**, 232 mg, 1.42 mmol, 1.2 equiv), and K_2CO_3 (246 mg, 1.77 mmol, 1.5 equiv) in THF (10 mL). After the reaction mixture was refluxed for 4.0 h and then worked up, the residue was purified by use of column chromatography (25% EtOAc in hexanes as the eluent) to give the desired thioether **4o** (172 mg, 0.889 mmol) in 75% yield as a colorless liquid: TLC R_f 0.48 (40% EtOAc in hexanes as the eluent); ^{1}H NMR ($CDCl_3$, 400 MHz) δ 8.24 (d, J = 4.4 Hz, 1 H, ArH), 7.39 (t, J = 7.8 Hz, 1 H, ArH), 7.12 (d, J = 8.0 Hz, 1 H, ArH), 6.90 (t, J = 6.0 Hz, 1 H, ArH), 3.97–3.93 (m, 1 H, SCHCO), 2.49–2.29 (m, 3 H, CH_2CO + ½ × CH_2CHS), 2.15–2.05 (m, 2 H, ½ × CH_2CHS + ½ × $CH_2CH_2CH_2$), 1.93–1.84 (m, 1 H, ½ × $CH_2CH_2CH_2$); $^{13}C\{^1H\}$ NMR ($CDCl_3$, 100 MHz) δ 214.5 (C=O), 156.4, 148.6, 135.9, 122.0, 119.6, 48.9 (S*C*HCO), 36.8 (*C*H_2CO), 30.2 (*C*H_2CHS), 20.6 (CH_2*C*H_2CH_2); IR (neat) 2965 (s, C–H stretch), 1740 (s, C=O), 1578 (s), 1415 (s), 1281 (m), 1125 (s), 987 (w), 760 (s) cm^{-1}; HRMS (ESI-TOF) m/z: [M + H]$^+$ Calcd for $C_{10}H_{12}NOS$ 194.0639; Found 194.0639.

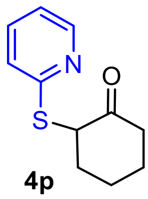

4p

2-(Pyridin-2-ylthio)cyclohexan-1-one (4p). The standard procedure 1 was followed by use of 2-mercaptopyridine (**5a**, 129 mg, 1.16 mmol, 1.0 equiv), 2-bromocyclohexanone (**6c**, 248 mg, 1.39 mmol, 1.2 equiv), and K_2CO_3 (241 mg, 1.74 mmol, 1.5 equiv) in THF (10 mL). After the reaction mixture was refluxed for 4.0 h and then worked up, the residue was purified by use of column chromatography (20% EtOAc in hexanes

as the eluent) to give the desired thioether **4p** (183 mg, 0.883 mmol) in 76% yield as a colorless liquid: TLC R_f 0.47 (40% EtOAc in hexanes as the eluent); ^{1}H NMR ($CDCl_3$, 400 MHz) δ 8.27 (d, J = 4.4 Hz, 1 H, ArH), 7.38 (t, J = 7.6 Hz, 1 H, ArH), 7.12 (d, J = 7.6 Hz, 1 H, ArH), 6.88 (t, J = 6.0 Hz, 1 H, ArH), 4.66–4.62 (m, 1 H, SCHCO), 2.63–2.58 (m, 1 H, ½ × CH_2CO), 2.42–2.37 (m, 2 H, ½ × CH_2CO + ½ × CH_2CHS), 1.97–1.95 (m, 1 H, ½ × CH_2CHS), 1.85–1.74 (m, 4 H, CH_2CH_2CO + CH_2CH_2CH); $^{13}C\{^1H\}$ NMR ($CDCl_3$, 100 MHz) δ 206.6 (C=O), 157.0, 149.0, 135.8, 122.5, 119.5, 53.0 (S*C*HCO), 41.0 (*C*H_2CO), 34.6 (*C*H_2CHS), 27.3 (*C*H_2CH_2CO), 24.5 (*C*H_2CH_2CHS); IR (neat) 2940 (s, C–H stretch), 1713 (s, C=O), 1578 (s), 1415 (s), 1293 (m), 1123 (s), 986 (w), 761 (s) cm^{-1}; HRMS (ESI-TOF) m/z: $[M + H]^+$ Calcd for $C_{11}H_{14}NOS$ 208.0796; Found 208.0798.

Experimental Data of α,α-Diarylated Cycloalkanones or Lactones

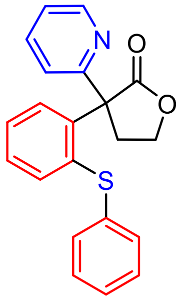

1ag

3-[2-(Phenylthio)phenyl]-3-(pyridin-2-yl)dihydrofuran-2(3*H*)-one (1ag). The standard procedure 2 was followed by use of 2-silylphenyl triflate **7a** (77.1 mg, 0.258 mmol, 2.0 equiv), thioether **4g** (25.2 mg, 0.129 mmol, 1.0 equiv), and CsF (78.4 mg, 0.516 mmol, 4.0 equiv) in CH_3CN (2.0 mL). After the reaction mixture was stirred for 16 h and then worked up, the residue was purified by use of column chromatography (12% EtOAc in hexanes as the eluent) to give the desired thioether lactone **1ag** (36.9 mg, 0.106 mmol) in 82% yield as white solids: mp (recrystallized from MeOH) 138.2–139.6 °C; TLC R_f 0.36 (20% EtOAc in hexanes as the eluent); ^{1}H NMR ($CDCl_3$, 400 MHz) δ 8.58 (d, J = 4.0 Hz, 1 H, ArH), 7.57 (t, J = 7.0 Hz, 1 H,

ArH), 7.29–7.16 (m, 9 H, 9 × ArH), 7.07 (d, J = 6.8 Hz, 2 H, 2 × ArH), 4.55–4.50 (m, 1 H, ½ × OCH_2), 4.37–4.31 (m, 1 H, ½ × OCH_2), 4.20–4.13 (m, 1 H, ½ × CH_2CCO), 2.80–2.73 (m, 1 H, ½ × CH_2CCO); $^{13}C\{^1H\}$ NMR ($CDCl_3$, 100 MHz) δ 177.7 (C=O), 158.0, 148.4, 142.0, 136.7, 136.3, 135.5, 135.2, 130.1, 129.2, 129.0, 128.5, 127.5, 126.8, 124.6, 122.5, 66.6 (OCH_2), 60.4 (C), 34.5 (C*C*H_2); IR (neat) 3056 (w), 1768 (s, C=O), 1584 (m), 1466 (s), 1161 (s), 1026 (m), 959 (w), 750 (s) cm^{-1}; HRMS (ESI-TOF) m/z: $[M + H]^+$ Calcd for $C_{21}H_{18}NO_2S$ 348.1058; Found 348.1060.

Gram-Scale Synthesis of 1ag.

The standard procedure 2 was followed by use of 2-silylphenyl triflate **7a** (3.22 g, 10.8 mmol, 2.0 equiv), thioether **4g** (1.05 g, 5.37 mmol, 1.0 equiv), and CsF (3.27 g, 21.5 mmol, 4.0 equiv) in CH_3CN (80 mL). After the reaction mixture was stirred for 18 h at 25 °C and then worked up, the residue was purified by use of column chromatography (12% EtOAc in hexanes as the eluent) to give the desired thioether lactone **1ag** (1.56 g, 4.49 mmol) in 83% isolated yield.

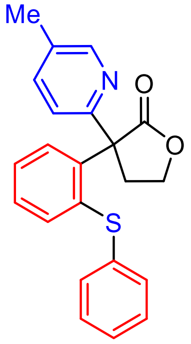

1ah

3-(5-Methylpyridin-2-yl)-3-[2-(Phenylthio)phenyl]dihydrofuran-2(3*H*)-one (1ah). The standard procedure 2 was followed by use of 2-silylphenyl triflate **7a** (76.4 mg, 0.256 mmol, 2.0 equiv), thioether **4h** (26.8 mg, 0.128 mmol, 1.0 equiv), and CsF (79.8 mg, 0.525 mmol, 4.1 equiv) in CH_3CN (2.1 mL). After the reaction mixture was stirred for 16 h and then worked up, the residue was purified by use of column chromatography (10% EtOAc in hexanes as the

eluent) to give the desired thioether lactone **1ah** (37.2 mg, 0.103 mmol) in 80% yield as a colorless liquid: TLC R_f 0.38 (20% EtOAc in hexanes as the eluent); ^{1}H NMR ($CDCl_3$, 400 MHz) δ 8.40 (s, 1 H, ArH), 7.37 (d, J = 8.0 Hz, 1 H, ArH), 7.31–7.15 (m, 7 H, 7 × ArH), 7.08 (t, J = 7.6 Hz, 3 H, 3 × ArH), 4.54–4.46 (m, 1 H, ½ × OCH_2), 4.36–4.30 (m, 1 H, ½ × OCH_2), 4.16–4.09 (m, 1 H, ½ × CH_2CCO), 2.80–2.73 (m, 1 H, ½ × CH_2CCO), 2.29 (s, 3 H, CH_3); $^{13}C\{^1H\}$ NMR ($CDCl_3$, 100 MHz) δ 177.9 (C=O), 155.0, 148.9, 142.3, 137.4, 136.5, 135.7, 135.0, 132.1, 130.0, 129.3, 129.0, 128.5, 127.5, 126.7, 124.0, 66.6 (OCH_2), 60.1 (C), 34.5 ($C\mathit{C}H_2$), 18.1 (CH_3); IR (neat) 3000 (w), 1764 (s, C=O), 1581 (w), 1477 (s), 1159 (s), 1028 (s), 961 (w), 751 (m) cm^{-1}; HRMS (ESI-TOF) m/z: $[M + H]^+$ Calcd for $C_{22}H_{20}NO_2S$ 362.1214; Found 362.1207.

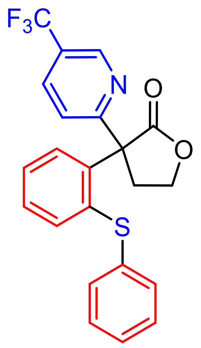

1ai

3-[2-(Phenylthio)phenyl]-3-[5-(trifluoromethyl)pyridin-2-yl]dihydrofuran-2(3*H*)-one (1ai). The standard procedure 2 was followed by use of 2-silylphenyl triflate **7a** (72.2 mg, 0.242 mmol, 2.0 equiv), thioether **4i** (31.9 mg, 0.121 mmol, 1.0 equiv), and CsF (73.6 mg, 0.484 mmol, 4.0 equiv) in CH_3CN (2.0 mL). After the reaction mixture was stirred for 18 h and then worked up, the residue was purified by use of column chromatography (12% EtOAc in hexanes as the eluent) to give the desired thioether lactone **1ai** (38.8 mg, 93.3 μmol) in 77% yield as a pale yellow colorless liquid: TLC R_f 0.47 (20% EtOAc in hexanes as the eluent); ^{1}H NMR ($CDCl_3$, 400 MHz) δ 8.80 (s, 1 H, ArH), 7.67 (d, J = 7.6 Hz, 1 H, ArH), 7.44 (d, J = 7.2 Hz, 1 H, ArH), 7.34–7.16 (m, 7 H, 7 × ArH), 6.97 (d, J = 6.8 Hz, 2 H, 2 × ArH), 4.60–4.55 (m,

1 H, ½ × OCH_2), 4.35–4.29 (m, 1 H, ½ × OCH_2), 4.26–4.19 (m, 1 H, ½ × CH_2CCO), 2.79–2.73 (m, 1 H, ½ × CH_2CCO); $^{13}C\{^1H\}$ NMR ($CDCl_3$, 100 MHz) δ 176.8 (C=O), 162.1, 145.3 (q, J = 4.0 Hz), 141.3, 136.3, 135.6, 134.4, 133.6 (q, J = 3.3 Hz), 129.5, 129.2, 129.1, 129.0, 128.0, 126.9, 125.2 (q, J = 32.9 Hz), 124.2, 123.4 (q, J = 270.7 Hz), 66.6 (OCH_2), 60.9 (C), 34.6 (CCH_2); IR (neat) 3061 (w), 1770 (s, C=O), 1606 (m), 1329 (s), 1133 (s), 1026 (m), 965 (w), 754 (m) cm^{-1}; HRMS (ESI-TOF) m/z: $[M + H]^+$ Calcd for $C_{22}H_{17}F_3NO_2S$ 416.0932; Found 416.0931.

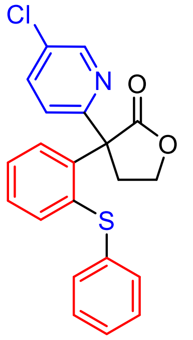

1aj

3-(5-Chloropyridin-2-yl)-3-[2-(Phenylthio)phenyl]dihydrofuran-2(3*H*)-one (1aj). The standard procedure 2 was followed by use of 2-silylphenyl triflate **7a** (78.2 mg, 0.262 mmol, 2.0 equiv), thioether **4j** (30.1 mg, 0.131 mmol, 1.0 equiv), and CsF (81.6 mg, 0.537 mmol, 4.1 equiv) in CH_3CN (2.2 mL). After the reaction mixture was stirred for 16 h and then worked up, the residue was purified by use of column chromatography (10% EtOAc in hexanes as the eluent) to give the desired thioether lactone **1aj** (39.1 mg, 0.102 mmol) in 78% yield as a colorless liquid: TLC R_f 0.38 (15% EtOAc in hexanes as the eluent); ^1H NMR ($CDCl_3$, 400 MHz) δ 8.50 (s, 1 H, ArH), 7.47 (d, J = 7.2 Hz, 1 H, ArH), 7.35–7.18 (m, 7 H, 7 × ArH), 7.09–7.02 (m, 3 H, 3 × ArH), 4.55–4.49 (m, 1 H, ½ × OCH_2), 4.34–4.28 (m, 1 H, ½ × OCH_2), 4.16–4.09 (m, 1 H, ½ × CH_2CCO), 2.78–2.71 (m, 1 H, ½ × CH_2CCO); $^{13}C\{^1H\}$ NMR ($CDCl_3$, 100 MHz) δ 177.3 (C=O), 156.3, 147.2, 141.5, 136.3, 135.8, 134.9, 131.0, 129.9, 129.4, 129.2, 129.1, 128.8, 127.7, 126.9, 125.3, 66.6 (OCH_2), 60.2 (C), 34.5 (CCH_2); IR (neat) 3058 (w),

1764 (s, C=O), 1580 (w), 1464 (s), 1160 (s), 1026 (m), 960 (w), 756 (m) cm^{-1}; HRMS (ESI-TOF) m/z: $[M + H]^+$ Calcd for $C_{21}H_{17}ClNO_2S$ 382.0668; Found 382.0660.

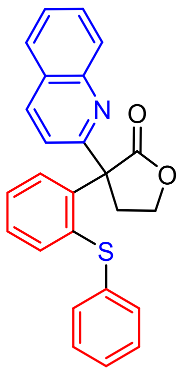

1ak

3-[2-(Phenylthio)phenyl]-3-(quinolin-2-yl)dihydrofuran-2(3*H*)-one (1ak). The standard procedure 2 was followed by use of 2-silylphenyl triflate **7a** (78.2 mg, 0.262 mmol, 2.0 equiv), thioether **4k** (32.1 mg, 0.131 mmol, 1.0 equiv), and CsF (79.6 mg, 0.524 mmol, 4.0 equiv) in CH_3CN (2.2 mL). After the reaction mixture was stirred for 16 h and then worked up, the residue was purified by use of column chromatography (10% EtOAc in hexanes as the eluent) to give the desired thioether lactone **1ak** (42.2 mg, 0.106 mmol) in 81% yield as a colorless liquid: TLC R_f 0.39 (20% EtOAc in hexanes as the eluent); ^1H NMR ($CDCl_3$, 400 MHz) δ 8.09 (d, J = 8.4 Hz, 1 H, ArH), 8.03 (d, J = 8.8 Hz, 1 H, ArH), 7.77 (d, J = 8.0 Hz, 1 H, ArH), 7.70 (t, J = 7.4 Hz, 1 H, ArH), 7.53 (t, J = 7.4 Hz, 1 H, ArH), 7.38–7.13 (m, 8 H, 8 × ArH), 7.05 (d, J = 7.6 Hz, 2 H, 2 × ArH), 4.66–4.59 (m, 1 H, ½ × OCH_2), 4.44–4.38 (m, 2 H, ½ × OCH_2 + ½ × CH_2CCO), 2.83–2.76 (m, 1 H, ½ × CH_2CCO); $^{13}C\{^1H\}$ NMR ($CDCl_3$, 100 MHz) δ 177.6 (C=O), 157.5, 147.1, 142.1, 136.6, 136.3, 135.6, 135.2, 130.1, 129.6, 129.5, 129.4, 129.0, 128.6, 127.6, 127.5, 127.3, 126.8, 126.7, 121.9, 66.9 (OCH_2), 60.9 (C), 34.4 (CCH_2); IR (neat) 3058 (w), 1760 (s, C=O), 1597 (w), 1476 (m), 1158 (s), 1025 (m), 959 (w), 755 (s) cm^{-1}; HRMS (ESI-TOF) m/z: $[M + H]^+$ Calcd for $C_{25}H_{20}NO_2S$ 398.1214; Found 398.1214.

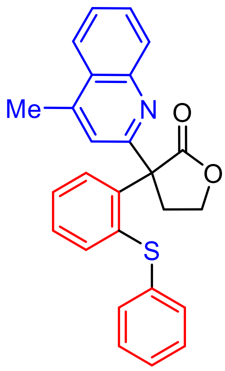

1al

3-(4-Methylquinolin-2-yl)-3-[2-(Phenylthio)phenyl]dihydrofuran-2(3*H*)-one (1al). The standard procedure 2 was followed by use of 2-silylphenyl triflate **7a** (72.8 mg, 0.244 mmol, 2.0 equiv), thioether **4l** (31.7 mg, 0.122 mmol, 1.0 equiv), and CsF (74.2 mg, 0.488 mmol, 4.0 equiv) in CH_3CN (2.1 mL). After the reaction mixture was stirred for 18 h and then worked up, the residue was purified by use of column chromatography (15% EtOAc in hexanes as the eluent) to give the desired thioether lactone **1al** (38.2 mg, 92.8 μmol) in 76% yield as a colorless liquid: TLC R_f 0.46 (20% EtOAc in hexanes as the eluent); ^{1}H NMR ($CDCl_3$, 400 MHz) δ 8.08 (d, J = 8.4 Hz, 1 H, ArH), 7.92 (d, J = 8.0 Hz, 1 H, ArH), 7.69 (t, J = 7.6 Hz, 1 H, ArH), 7.54 (t, J = 7.6 Hz, 1 H, ArH), 7.36 (t, J = 4.2 Hz, 1 H, ArH), 7.25–7.11 (m, 7 H, 7 × ArH), 7.03 (d, J = 6.8 Hz, 2 H, 2 × ArH), 4.63–4.57 (m, 1 H, ½ × OCH_2), 4.45–4.36 (m, 2 H, ½ × OCH_2 + ½ × CH_2CCO), 2.80–2.73 (m, 1 H, ½ × CH_2CCO), 2.55 (s, 3 H, CH_3); $^{13}C\{^1H\}$ NMR ($CDCl_3$, 100 MHz) δ 177.7 (C=O), 157.0, 147.0, 144.8, 142.7, 136.6, 136.2, 134.8, 130.0, 129.7, 129.6, 129.0, 128.9, 128.5, 127.7, 127.4, 126.5, 126.4, 123.6, 122.3, 66.9 (OCH_2), 60.8 (C), 34.5 (C*C*H_2), 18.7 (CH_3); IR (neat) 3058 (w), 1763 (s, C=O), 1598 (m), 1476 (m), 1158 (s), 1025 (m), 961 (w), 760 (s) cm^{-1}; HRMS (ESI-TOF) m/z: $[M + H]^+$ Calcd for $C_{26}H_{22}NO_2S$ 412.1371; Found 412.1371.

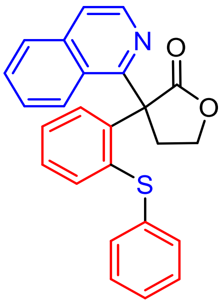

1am

3-(Isoquinolin-1-yl)-3-[2-(phenylthio)phenyl]dihydrofuran-2(3*H*)-one (1am). The standard procedure 2 was followed by use of 2-silylphenyl triflate **7a** (78.8 mg, 0.264 mmol, 2.0 equiv), thioether **4m** (32.4 mg, 0.132 mmol, 1.0 equiv), and CsF (80.3 mg, 0.528 mmol, 4.0 equiv) in CH_3CN (2.2 mL). After the reaction mixture was stirred for 18 h and then worked up, the residue was purified by use of column chromatography (10% EtOAc in hexanes as the eluent) to give the desired thioether lactone **1am** (40.6 mg, 0.102 mmol) in 77% yield as white solids: mp (recrystallized from MeOH) 189.8–191.7 °C: TLC R_f 0.42 (20% EtOAc in hexanes as the eluent); ^{1}H NMR ($CDCl_3$, 400 MHz) δ 8.48 (d, J = 5.6 Hz, 1 H, ArH), 7.97 (d, J = 8.4 Hz, 1 H, ArH), 7.69 (d, J = 8.0 Hz, 1 H, ArH), 7.56–7.49 (m, 3 H, 3 × ArH), 7.26 (d, J = 7.6 Hz, 2 H, 2 × ArH), 7.18 (d, J = 6.4 Hz, 2 H, 2 × ArH), 7.04 (s, 3 H, 3 × ArH), 6.69 (s, 2 H, 2 × ArH), 4.70 (d, J = 5.6 Hz, 1 H, ½ × OCH_2), 4.48–4.43 (m, 1 H, ½ × OCH_2), 4.23 (br s, 1 H, ½ × CH_2CCO), 2.70 (br s, 1 H, ½ × CH_2CCO); $^{13}C\{^1H\}$ NMR ($CDCl_3$, 100 MHz) δ 176.5 (C=O), 156.1, 143.3, 140.7, 137.6, 135.7, 133.9, 133.0, 131.0, 129.8, 129.4, 128.8, 128.5, 128.3, 127.9, 127.2, 127.0, 126.4, 126.2, 121.6, 67.1 (OCH_2), 61.5 (C), 37.5 (CCH_2); IR (neat) 3054 (w), 1764 (s, C=O), 1583 (m), 1475 (m), 1154 (s), 1031 (m), 827 (w), 748 (s) cm^{-1}; HRMS (ESI-TOF) m/z: $[M + H]^+$ Calcd for $C_{25}H_{20}NO_2S$ 398.1214; Found 398.1208.

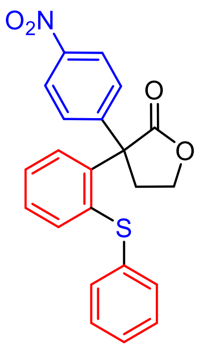

1an

3-(4-Nitrophenyl)-3-[2-(phenylthio)phenyl]dihydrofuran-2(3*H*)-one (1an). The standard procedure 2 was followed by use of 2-silylphenyl triflate **7a** (76.4 mg, 0.256 mmol, 2.0 equiv), thioether **4n**[4] (30.7 mg, 0.128 mmol, 1.0 equiv), and CsF (77.8 mg, 0.512 mmol, 4.0 equiv) in CH_3CN (2.0 mL). After the reaction mixture was stirred for 18 h and then worked up, the residue was purified by use of column chromatography (20% EtOAc in hexanes as the eluent) to give the desired thioether lactone **1an** (35.1 mg, 89.6 μmol) in 70% yield as a colorless liquid: TLC R_f 0.45 (40% EtOAc in hexanes as the eluent); ^{1}H NMR ($CDCl_3$, 400 MHz) δ 8.08 (d, J = 8.8 Hz, 2 H, 2 × ArH), 7.51 (d, J = 9.2 Hz, 2 H, 2 × ArH), 7.40–7.38 (m, 1 H, ArH), 7.33–7.28 (m, 3 H, 3 × ArH), 7.17–7.13 (m, 3 H, 3 × ArH), 6.93–6.90 (m, 2 H, 2 × ArH), 4.50–4.45 (m, 1 H, ½ × OCH_2), 4.30–4.24 (m, 1 H, ½ × OCH_2), 3.52–3.45 (m, 1 H, ½ × CH_2CCO), 3.15–3.09 (m, 1 H, ½ × CH_2CCO); $^{13}C\{^1H\}$ NMR ($CDCl_3$, 100 MHz) δ 176.4 (C=O), 146.9, 145.9, 141.0, 136.6, 135.5, 134.4, 130.1, 129.6, 129.2, 129.1, 129.0, 127.9, 127.0, 123.3, 65.6 (OCH_2), 58.1 (C), 34.9 (CCH_2); IR (neat) 3065 (w), 1770 (s, C=O), 1596 (w), 1519 (s), 1347 (s), 1157 (s), 1025 (m), 752 (m) cm^{-1}; HRMS (ESI-TOF) m/z: $[M + Na]^+$ Calcd for $C_{22}H_{17}NO_4SNa$ 414.0776; Found 414.0779.

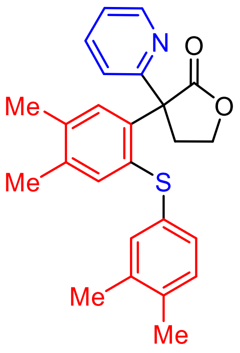

1bg

3-(2-[(3,4-Dimethylphenyl)thio]-4,5-dimethylphenyl)-3-(pyridin-2-yl)dihydrofuran-2(3*H*)-one (1bg). The standard procedure 2 was followed by use of 4,5-dimethyl-2-(trimethylsilyl)phenyl trifluoromethanesulfonate[2] (**7b**, 79.6 mg, 0.244 mmol, 2.0 equiv), thioether **4g** (23.9 mg, 0.122 mmol, 1.0 equiv), and CsF (74.1 mg, 0.488 mmol, 4.0 equiv) in CH_3CN (2.2 mL). After the reaction mixture was stirred for 16 h and then worked up, the residue was purified by use of column chromatography (10% EtOAc in hexanes as the eluent) to give the desired thioether lactone **1bg** (40.8 mg, 0.101 mmol) in 83% yield as a colorless liquid: TLC R_f 0.41 (15% EtOAc in hexanes as the eluent); ^{1}H NMR ($CDCl_3$, 400 MHz) δ 8.57 (d, J = 4.0 Hz, 1 H, ArH), 7.55 (t, J = 7.2 Hz, 1 H, ArH), 7.16 (d, J = 7.6 Hz, 2 H, 2 × ArH), 7.07 (s, 2 H, 2 × ArH), 6.94 (d, J = 7.6 Hz, 1 H, ArH), 6.84 (s, 1 H, ArH), 6.76 (d, J = 7.6 Hz, 1 H, ArH), 4.53–4.48 (m, 1 H, ½ × OCH_2), 4.33–4.27 (m, 1 H, ½ × OCH_2), 4.16–4.09 (m, 1 H, ½ × CH_2CCO), 2.79–2.73 (m, 1 H, ½ × CH_2CCO), 2.20 (s, 3 H, CH_3), 2.17 (s, 3 H, CH_3), 2.14 (s, 3 H, CH_3), 2.13 (s, 3 H, CH_3); $^{13}C\{^1H\}$ NMR ($CDCl_3$, 100 MHz) δ 178.0 (C=O), 158.6, 148.3, 139.1, 137.3, 137.1, 136.6, 136.5, 136.1, 135.2, 133.3, 131.6, 131.0, 130.3, 130.2, 127.3, 124.5, 122.2, 66.6 (OCH_2), 60.2 (C), 34.7 ($C\mathit{C}H_2$), 19.6 (CH_3), 19.5 (CH_3), 19.3 (CH_3), 19.1 (CH_3); IR (neat) 2919 (m), 1769 (s, C=O), 1586 (m), 1429 (m), 1372 (m), 1159 (s), 1029 (m), 881 (w) cm^{-1}; HRMS (ESI-TOF) m/z: $[M + H]^+$ Calcd for $C_{25}H_{26}NO_2S$ 404.1684; Found 404.1685.

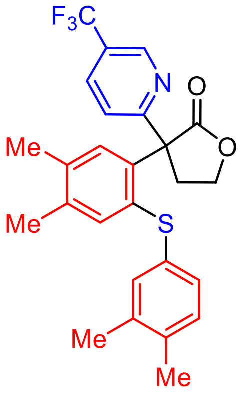

1bi

3-(2-[(3,4-Dimethylphenyl)thio]-4,5-dimethylphenyl)-3-[5-(trifluoromethyl)pyridin-2-yl]dihydrofuran-2(3*H*)-one (1bi). The standard procedure 2 was followed by use of 4,5-dimethyl-2-(trimethylsilyl)phenyl trifluoromethanesulfonate[2] (**7b**, 72.5 mg, 0.222 mmol, 2.0 equiv), thioether **4i** (29.3 mg, 0.111 mmol, 1.0 equiv), and CsF (67.5 mg, 0.444 mmol, 4.0 equiv) in CH_3CN (2.3 mL). After the reaction mixture was stirred for 18 h and then worked up, the residue was purified by use of column chromatography (10% EtOAc in hexanes as the eluent) to give the desired thioether lactone **1bi** (38.3 mg, 81.2 μmol) in 73% yield as a colorless liquid: TLC R_f 0.47 (20% EtOAc in hexanes as the eluent); ^{1}H NMR ($CDCl_3$, 400 MHz) δ 8.76 (s, 1 H, ArH), 7.60 (d, J = 8.4 Hz, 1 H, ArH), 7.21 (s, 1 H, ArH), 7.15–7.12 (m, 2 H, 2 × ArH), 6.90 (d, J = 8.0 Hz, 1 H, ArH), 6.69 (s, 1 H, ArH), 6.61 (d, J = 7.6 Hz, 1 H, ArH), 4.58–4.53 (m, 1 H, ½ × OCH_2), 4.31–4.15 (m, 2 H, ½ × OCH_2 + ½ × CH_2CCO), 2.77–2.69 (m, 1 H, ½ × CH_2CCO), 2.26 (s, 3 H, CH_3), 2.17 (s, 3 H, CH_3), 2.15 (s, 3 H, CH_3), 2.11 (s, 3 H, CH_3); $^{13}C\{^1H\}$ NMR ($CDCl_3$, 100 MHz) δ 177.2 (C=O), 162.6, 145.0 (q, J = 4.1 Hz), 138.7, 137.8, 137.7, 137.3, 136.9, 135.2, 133.3 (q, J = 3.6 Hz), 132.7, 130.5, 130.3, 130.2, 130.1, 126.3, 124.9 (q, J = 32.7 Hz), 124.3, 123.5 (q, J = 270.6 Hz), 66.6 (OCH_2), 60.6 (C), 34.9 (CCH_2), 19.7 (CH_3), 19.6 (CH_3), 19.2 (CH_3), 19.1 (CH_3); IR (neat) 2922 (m), 1767 (s, C=O), 1605 (m), 1449 (m), 1328 (s), 1133 (s), 1017 (m), 962 (w) cm^{-1}; HRMS (ESI-TOF) m/z: $[M + H]^+$ Calcd for $C_{26}H_{25}F_3NO_2S$ 472.1558; Found 472.1557.

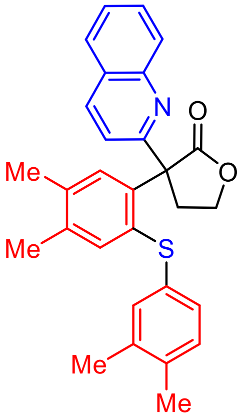

1bk

3-(2-[(3,4-Dimethylphenyl)thio]-4,5-dimethylphenyl)-3-(quinolin-2-yl)dihydrofuran-2(3*H*)-one (1bk). The standard procedure 2 was followed by use of 4,5-dimethyl-2-(trimethylsilyl)phenyl trifluoromethanesulfonate[2] (**7b**, 80.3 mg, 0.246 mmol, 2.0 equiv), thioether **4k** (30.2 mg, 0.123 mmol, 1.0 equiv), and CsF (74.8 mg, 0.492 mmol, 4.0 equiv) in CH_3CN (2.2 mL). After the reaction mixture was stirred for 16 h and then worked up, the residue was purified by use of column chromatography (10% EtOAc in hexanes as the eluent) to give the desired thioether lactone **1bk** (43.6 mg, 96.1 μmol) in 78% yield as a colorless liquid: TLC R_f 0.52 (20% EtOAc in hexanes as the eluent); ^{1}H NMR ($CDCl_3$, 400 MHz) δ 8.08 (d, J = 8.4 Hz, 1 H, ArH), 7.97 (d, J = 8.4 Hz, 1 H, ArH), 7.75 (d, J = 7.6 Hz, 1 H, ArH), 7.68 (t, J = 7.8 Hz, 1 H, ArH), 7.51 (t, J = 7.6 Hz, 1 H, ArH), 7.32 (d, J = 8.4 Hz, 1 H, ArH), 7.11 (s, 1 H, ArH), 7.07 (s, 1 H, ArH), 6.90 (d, J = 7.6 Hz, 1 H, ArH), 6.77 (s, 1 H, ArH), 6.73 (d, J = 8.0 Hz, 1 H, ArH), 4.65–4.59 (m, 1 H, ½ × OCH_2), 4.44–4.36 (m, 2 H, ½ × OCH_2 + ½ × CH_2CCO), 2.81–2.77 (m, 1 H, ½ × CH_2CCO), 2.20 (s, 3 H, CH_3), 2.15 (s, 6 H, 2 × CH_3), 2.07 (s, 3 H, CH_3); $^{13}C\{^1H\}$ NMR ($CDCl_3$, 100 MHz) δ 177.8 (C=O), 158.1, 147.1, 139.4, 137.3, 137.2, 136.9, 136.4, 136.3, 135.0, 133.3, 131.5, 130.8, 130.5, 130.1, 129.5, 129.2, 127.4, 127.2, 127.1, 126.5, 122.0, 66.8 (OCH_2), 60.7 (C), 34.6 ($C\mathit{C}H_2$), 19.6 (CH_3), 19.5 (CH_3), 19.2 (CH_3), 19.1 (CH_3); IR (neat) 2919 (m), 1760 (s, C=O), 1597 (m), 1372 (m), 1160 (s), 1028 (m), 880 (w), 736 (m) cm^{-1}; HRMS (ESI-TOF) m/z: $[M + H]^+$ Calcd for $C_{29}H_{28}NO_2S$ 454.1840; Found 454.1840.

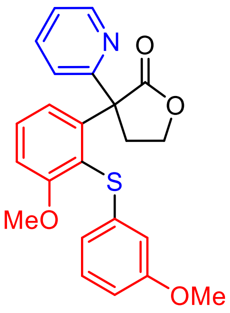

1cg

3-(3-Methoxy-2-[(3-methoxyphenyl)thio]phenyl)-3-(pyridin-2-yl)dihydrofuran-2(3*H*)-one (1cg). The standard procedure 2 was followed by use of 3-methoxy-2-(trimethylsilyl)phenyl trifluoromethanesulfonate **7c** (81.5 mg, 0.248 mmol, 2.0 equiv), thioether **4g** (24.2 mg, 0.124 mmol, 1.0 equiv), and CsF (77.2 mg, 0.508 mmol, 4.1 equiv) in CH_3CN (2.1 mL). After the reaction mixture was stirred for 16 h and then worked up, the residue was purified by use of column chromatography (20% EtOAc in hexanes as the eluent) to give the desired thioether lactone **1cg** (42.8 mg, 0.105 mmol) in 85% yield as a colorless liquid: TLC R_f 0.30 (30% EtOAc in hexanes as the eluent); ^{1}H NMR ($CDCl_3$, 400 MHz) δ 8.53 (d, J = 4.0 Hz, 1 H, ArH), 7.44 (t, J = 7.2 Hz, 1 H, ArH), 7.37 (t, J = 8.0 Hz, 1 H, ArH), 7.13 (d, J = 8.0 Hz, 1 H, ArH), 7.09 (t, J = 6.0 Hz, 1 H, ArH), 7.01–6.92 (m, 3 H, 3 × ArH), 6.55 (d, J = 7.6 Hz, 1 H, ArH), 6.34 (d, J = 1.6 Hz, 2 H, 2 × ArH), 4.48–4.43 (m, 1 H, ½ × OCH_2), 4.32–4.26 (m, 1 H, ½ × OCH_2), 4.11–4.05 (m, 1 H, ½ × CH_2CCO), 3.70 (s, 3 H, OCH_3), 3.67 (s, 3 H, OCH_3), 2.65–2.58 (m, 1 H, ½ × CH_2CCO); ^{13}C{^{1}H} NMR ($CDCl_3$, 100 MHz) δ 177.8 (C=O), 161.7, 159.5, 158.4, 148.3, 147.2, 138.5, 136.5, 130.7, 129.1, 124.3, 122.2, 121.9, 119.0, 117.7, 111.3, 111.0, 110.3, 66.6 (OCH_2), 61.0 (C), 56.2 (OCH_3), 55.1 (OCH_3), 35.1 (C*C*H_2); IR (neat) 2938 (w), 1769 (s, C=O), 1589 (s), 1469 (s), 1280 (m), 1161 (s), 1030 (s), 778 (m) cm^{-1}; HRMS (ESI-TOF) m/z: $[M + H]^+$ Calcd for $C_{23}H_{22}NO_4S$ 408.1269; Found 408.1269.

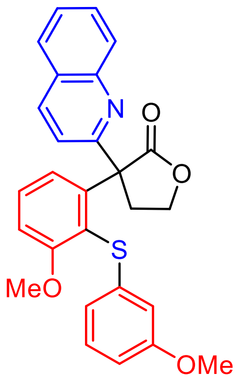

1ck

3-(3-Methoxy-2-[(3-methoxyphenyl)thio]phenyl)-3-(quinolin-2-yl)dihydrofuran-2(3*H*)-one (1ck). The standard procedure 2 was followed by use of 3-methoxy-2-(trimethylsilyl)phenyl trifluoromethanesulfonate **7c** (75.8 mg, 0.231 mmol, 2.0 equiv), thioether **4k** (28.3 mg, 0.115 mmol, 1.0 equiv), and CsF (70.2 mg, 0.462 mmol, 4.0 equiv) in CH_3CN (2.1 mL). After the reaction mixture was stirred for 16 h and then worked up, the residue was purified by use of column chromatography (20% EtOAc in hexanes as the eluent) to give the desired thioether lactone **1ck** (42.7 mg, 93.3 μmol) in 81% yield as a colorless liquid: TLC R_f 0.46 (30% EtOAc in hexanes as the eluent); ^{1}H NMR ($CDCl_3$, 400 MHz) δ 8.06 (d, J = 8.0 Hz, 1 H, ArH), 7.89 (d, J = 8.8 Hz, 1 H, ArH), 7.72–7.67 (m, 2 H, ArH), 7.50 (t, J = 7.4 Hz, 1 H, ArH), 7.36 (t, J = 8.2 Hz, 1 H, ArH), 7.31 (d, J = 8.4 Hz, 1 H, ArH), 6.98–6.92 (m, 2 H, 2 × ArH), 6.87 (d, J = 7.2 Hz, 1 H, ArH), 6.52 (d, J = 8.0 Hz, 1 H, ArH), 6.32 (d, J = 8.0 Hz, 1 H, ArH), 6.29 (s, 1 H, ArH), 4.60–4.54 (m, 1 H, ½ × OCH_2), 4.41–4.32 (m, 2 H, ½ × OCH_2 + ½ × CH_2CCO), 3.72 (s, 3 H, OCH_3), 3.58 (s, 3 H, OCH_3), 2.70–2.63 (m, 1 H, ½ × CH_2CCO); $^{13}C\{^1H\}$ NMR ($CDCl_3$, 100 MHz) δ 177.7 (C=O), 161.7, 159.4, 157.8, 147.3, 147.0, 138.4, 136.3, 130.7, 129.4, 129.2, 129.1, 127.4, 127.2, 126.5, 122.1, 121.9, 119.1, 117.7, 111.3, 110.9, 110.5, 66.8 (OCH_2), 61.4 (C), 56.2 (OCH_3), 54.9 (OCH_3), 34.9 (CCH_2); IR (neat) 2939 (m), 1760 (s, C=O), 1590 (s), 1470 (s), 1280 (s), 1161 (s), 1030 (s), 735 (m) cm^{-1}; HRMS (ESI-TOF) m/z: $[M + H]^+$ Calcd for $C_{27}H_{24}NO_4S$ 458.1426; Found 458.1426.

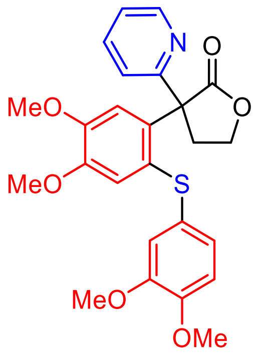

1dg

3-(2-[(3,4-Dimethoxyphenyl)thio]-4,5-dimethoxyphenyl)-3-(pyridin-2-yl)dihydrofuran-2(3*H*)-one (1dg). The standard procedure 2 was followed by use of 4,5-dimethoxy-2-(trimethylsilyl)phenyl trifluoromethanesulfonate (**7d**, 81.8 mg, 0.228 mmol, 2.0 equiv), thioether **4g** (22.3 mg, 0.114 mmol, 1.0 equiv), and CsF (71.1 mg, 0.467 mmol, 4.1 equiv) in CH_3CN (2.2 mL). After the reaction mixture was stirred for 18 h and then worked up, the residue was purified by use of column chromatography (40% EtOAc in hexanes as the eluent) to give the desired thioether lactone **1dg** (33.7 mg, 72.1 μmol) in 63% yield as a colorless liquid: TLC R_f 0.21 (60% EtOAc in hexanes as the eluent); ^{1}H NMR ($CDCl_3$, 400 MHz) δ 8.58 (d, J = 4.0 Hz, 1 H, ArH), 7.56 (t, J = 7.6 Hz, 1 H, ArH), 7.20–7.16 (m, 2 H, 2 × ArH), 6.86 (s, 1 H, ArH), 6.80 (s, 1 H, ArH), 6.70 (d, J = 8.8 Hz, 1 H, ArH), 6.60 (d, J = 5.2 Hz, 2 H, 2 × ArH), 4.58–4.52 (m, 1 H, ½ × OCH_2), 4.39–4.34 (m, 1 H, ½ × OCH_2), 4.17–4.10 (m, 1 H, ½ × CH_2CCO), 3.82 (s, 3 H, OCH_3), 3.78 (s, 3 H, OCH_3), 3.75 (s, 3 H, OCH_3), 3.68 (s, 3 H, OCH_3), 2.77–2.70 (m, 1 H, ½ × CH_2CCO); $^{13}C\{^{1}H\}$ NMR ($CDCl_3$, 100 MHz) δ 178.0 (C=O), 158.6, 149.2, 148.5, 148.4, 148.3, 148.2, 136.6, 135.2, 127.9, 126.0, 124.5, 122.5, 122.4, 118.2, 113.2, 112.3, 111.6, 66.8 (OCH_2), 59.9 (C), 55.9 (4 × OCH_3), 35.2 (CCH_2); IR (neat) 2926 (m), 1760 (m, C=O), 1585 (w), 1503 (s), 1325 (m), 1254 (m), 1160 (s), 1025 (s) cm^{-1}; HRMS (ESI-TOF) m/z: $[M + H]^+$ Calcd for $C_{25}H_{26}NO_6S$ 468.1480; Found 468.1468.

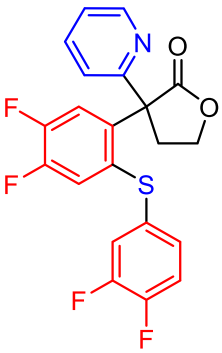

1eg

3-(2-[(3,4-Difluorophenyl)thio]-4,5-difluorophenyl)-3-(pyridin-2-yl)dihydrofuran-2(3*H*)-one (1eg). The standard procedure 2 was followed by use of 4,5-difluoro-2-(trimethylsilyl)phenyl trifluoromethanesulfonate[1] (**7e**, 74.9 mg, 0.224 mmol, 2.0 equiv), thioether **4g** (21.9 mg, 0.112 mmol, 1.0 equiv), and CsF (68.2 mg, 0.448 mmol, 4.0 equiv) in CH_3CN (2.0 mL). After the reaction mixture was stirred for 16 h and then worked up, the residue was purified by use of column chromatography (10% EtOAc in hexanes as the eluent) to give the desired thioether lactone **1eg** (39.5 mg, 94.1 μmol) in 84% yield as a colorless liquid: TLC R_f 0.36 (15% EtOAc in hexanes as the eluent); ^{1}H NMR ($CDCl_3$, 400 MHz) δ 8.59 (d, J = 4.0 Hz, 1 H, ArH), 7.63 (t, J = 7.2 Hz, 1 H, ArH), 7.28–7.22 (m, 2 H, 2 × ArH), 7.13–7.02 (m, 3 H, 3 × ArH), 6.93–6.87 (m, 2 H, 2 × ArH), 4.52–4.41 (m, 2 H, OCH_2), 4.14–4.08 (m, 1 H, ½ × CH_2CCO), 2.69–2.61 (m, 1 H, ½ × CH_2CCO); $^{13}C\{^1H\}$ NMR ($CDCl_3$, 100 MHz) δ 176.9 (C=O), 156.7, 150.4 (dd, J = 250.8, 13.2 Hz), 150.0 (dd, J = 249.2, 12.6 Hz), 149.5 (dd, J = 250.9, 11.7 Hz), 149.2 (dd, J = 246.6, 9.0 Hz), 148.9, 139.7 (m), 137.1, 131.5 (m), 131.0 (m), 126.8 (m), 124.3, 123.8 (d, J = 18.2 Hz), 123.1, 119.5 (d, J = 18.5 Hz), 119.0 (d, J = 19.8 Hz), 118.1 (d, J = 17.7 Hz), 66.7 (OCH_2), 59.4 (C), 34.9 ($C\mathit{C}H_2$); IR (neat) 3056 (w), 1761 (s, C=O), 1599 (s), 1503 (s), 1383 (m), 1154 (s), 1029 (m), 773 (m) cm^{-1}; HRMS (ESI-TOF) m/z: $[M + H]^+$ Calcd for $C_{21}H_{14}F_4NO_2S$ 420.0681; Found 420.0685.

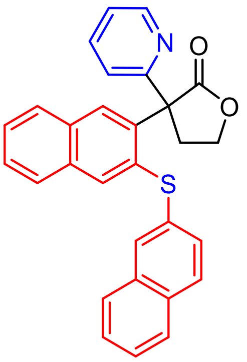

1fg

3-[3-(Naphthalen-2-ylthio)naphthalen-2-yl]-3-(pyridin-2-yl)dihydrofuran-2(3*H*)-one (1fg). The standard procedure 2 was followed by use of 3-(trimethylsilyl)-2-naphthyl trifluoromethanesulfonate (**7f**, 83.2 mg, 0.238 mmol, 2.0 equiv), thioether **4g** (23.3 mg, 0.119 mmol, 1.0 equiv), and CsF (72.4 mg, 0.476 mmol, 4.0 equiv) in CH_3CN (2.1 mL). After the reaction mixture was stirred for 16 h and then worked up, the residue was purified by use of column chromatography (10% EtOAc in hexanes as the eluent) to give the desired thioether lactone **1fg** (42.6 mg, 95.2 μmol) in 80% yield as a brown liquid: TLC R_f 0.44 (15% EtOAc in hexanes as the eluent); ^{1}H NMR ($CDCl_3$, 400 MHz) δ 8.60 (d, J = 4.4 Hz, 1 H, ArH), 7.94 (s, 1 H, ArH), 7.82–7.74 (m, 3 H, 3 × ArH), 7.66–7.56 (m, 4 H, 4 × ArH), 7.52–7.42 (m, 5 H, 5 × ArH), 7.19–7.11 (m, 3 H, 3 × ArH), 4.64–4.58 (m, 1 H, ½ × OCH_2), 4.38–4.27 (m, 2 H, ½ × OCH_2 + ½ × CH_2CCO), 2.92–2.85 (m, 1 H, ½ × CH_2CCO); $^{13}C\{^1H\}$ NMR ($CDCl_3$, 100 MHz) δ 177.7 (C=O), 158.2, 148.5, 138.5, 136.7, 135.5, 133.7, 133.6, 132.8, 132.4, 132.1, 132.0, 128.9, 128.8, 128.7, 128.1, 127.6, 127.5, 127.2, 127.1, 127.0, 126.7, 126.6, 126.1, 124.6, 122.4, 66.6 (OCH_2), 60.6 (C), 34.5 (CCH_2); IR (neat) 3053 (m), 1766 (s, C=O), 1585 (m), 1429 (m), 1160 (s), 1029 (m), 958 (w), 747 (s) cm^{-1}; HRMS (ESI-TOF) m/z: [M + H]$^+$ Calcd for $C_{29}H_{22}NO_2S$ 448.1371; Found 448.1375.

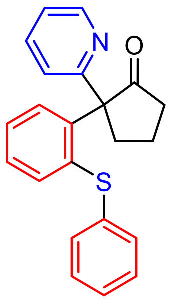

1ao

2-[2-(Phenylthio)phenyl]-2-(pyridin-2-yl)cyclopentan-1-one (1ao). The standard procedure 2 was followed by use of 2-silylphenyl triflate **7a** (89.1 mg, 0.298 mmol, 2.0 equiv), thioether **4o** (28.8 mg, 0.149 mmol, 1.0 equiv), and CsF (90.6 mg, 0.596 mmol, 4.0 equiv) in CH_3CN (2.0 mL). After the reaction mixture was stirred for 18 h and then worked up, the residue was purified by use of column chromatography (10% EtOAc in hexanes as the eluent) to give the desired thioether cycloketone **1ao** (38.7 mg, 0.112 mmol) in 75% yield as a colorless liquid: TLC R_f 0.38 (20% EtOAc in hexanes as the eluent); ^{1}H NMR ($CDCl_3$, 400 MHz) δ 8.60 (d, J = 4.0 Hz, 1 H, ArH), 7.55 (t, J = 7.4 Hz, 1 H, ArH), 7.31–7.08 (m, 10 H, 10 × ArH), 6.80 (d, J = 6.8 Hz, 1 H, ArH), 3.69–3.62 (m, 1 H, ½ × CH_2CO), 2.76–2.58 (m, 2 H, ½ × CH_2CO + ½ × CH_2CCO), 2.44–2.36 (m, 1 H, ½ × CH_2CCO), 2.09–1.95 (m, 2 H, CH_2CH_2CO); ^{13}C{^{1}H} NMR ($CDCl_3$, 100 MHz) δ 218.1 (C=O), 158.9, 148.6, 145.6, 137.3, 136.2, 135.9, 134.7, 129.7, 129.6, 128.9, 127.8, 127.2, 126.4, 125.1, 121.8, 66.7 (C), 38.5, 35.9, 19.7; IR (neat) 2951 (s), 1714 (s, C=O), 1584 (s), 1434 (s), 1152 (m), 1024 (w), 748 (s), 691 (m) cm^{-1}; HRMS (ESI-TOF) m/z: $[M + H]^+$ Calcd for $C_{22}H_{20}NOS$ 346.1265; Found 346.1263.

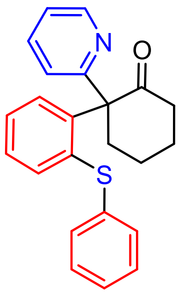

1ap

2-[2-(Phenylthio)phenyl]-2-(pyridin-2-yl)cyclohexan-1-one (1ap). The standard procedure 2 was followed by use of 2-silylphenyl triflate **7a** (99.2 mg, 0.332 mmol, 2.0 equiv), thioether **4p** (34.5 mg, 0.166 mmol, 1.0 equiv), and CsF (101 mg, 0.664 mmol, 4.0 equiv) in CH_3CN (2.5 mL). After the reaction mixture was stirred for 16 h and then worked up, the residue was purified by use of column chromatography (10% EtOAc in hexanes as the eluent) to give the desired thioether cycloketone **1ap** (46.5 mg, 0.129 mmol) in 78% yield as a white solids: mp (recrystallized from MeOH) 165.6–167.3 °C; TLC R_f 0.32 (20% EtOAc in hexanes as the eluent); ^{1}H NMR ($CDCl_3$, 400 MHz) δ 8.73 (d, J = 4.4 Hz, 1 H, ArH), 7.64 (t, J = 7.6 Hz, 1 H, ArH), 7.40–7.34 (m, 2 H, 2 × ArH), 7.25–7.08 (m, 7 H, 7 × ArH), 7.01 (t, J = 7.6 Hz, 1 H, ArH), 6.24 (d, J = 8.0 Hz, 1 H, ArH), 3.21–3.17 (m, 1 H, ½ × CH_2CO), 2.70–2.58 (m, 2 H, ½ × CH_2CO + ½ × CH_2CCO), 2.30–2.08 (m, 2 H, ½ × CH_2CCO + ½ × $C\mathit{H_2}CH_2CO$), 1.88–1.74 (m, 3 H, ½ × $C\mathit{H_2}CH_2CO$ + $C\mathit{H_2}CH_2CH_2CO$); ^{13}C{^{1}H} NMR ($CDCl_3$, 100 MHz) δ 210.3 (C=O), 158.7, 149.2, 148.5, 138.5, 136.6, 136.2, 134.1, 129.5, 128.9, 128.9, 127.6, 127.5, 126.1, 124.8, 122.1, 66.7 (C), 41.1, 36.3, 25.2, 22.8; IR (neat) 2942 (s), 1698 (s, C=O), 1583 (s), 1428 (s), 1233 (w), 1128 (m), 995 (w), 748 (s) cm^{-1}; HRMS (ESI-TOF) m/z: $[M + H]^+$ Calcd for $C_{23}H_{22}NOS$ 360.1422; Found 360.1424.

Table S1. Optimization of Conditions for the Reaction 7a + 4g → 1ag

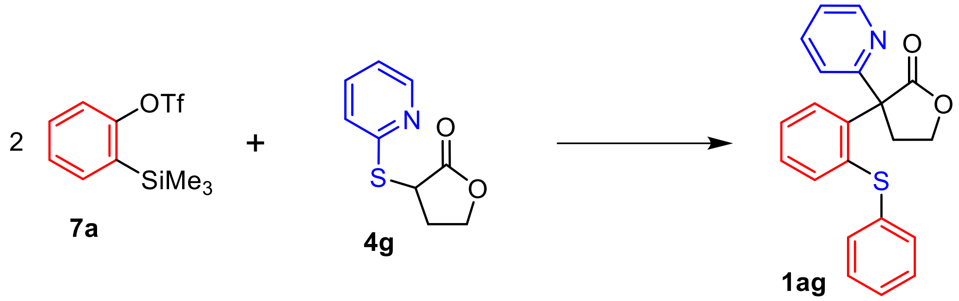

entry	F^- source (4.0 equiv)	solvent	additive (4.0 equiv)	temperature (°C)	time (h)	yield (%)
1	CsF	CH_3CN	-	25	8.0	53
2	CsF	CH_3CN	-	25	16	82
3	CsF	CH_3CN	-	40	16	48
4	TBAF	CH_3CN	-	25	16	27
5	CsF	THF	-	25	24	0
6	CsF	THF	-	60	24	7.0
7	KF	THF	18-crown-6	25	16	16
8	KF	THF	18-crown-6	60	16	11
9	TBAF	THF	-	25	16	13
10	KF	2-MeTHF	18-crown-6	25	16	19
11	KF	toluene	18-crown-6	25	18	5.0
12	KF	toluene	18-crown-6	70	18	12

References

(1) Peña, D.; Pérez, D.; Guitián, E.; Castedo, L. Synthesis of Hexabenzotriphenylene and Other Strained Polycyclic Aromatic Hydrocarbons by Palladium-Catalyzed Cyclotrimerization of Arynes. *J. Am. Chem. Soc.* **1999**, *121*, 5827–5828.

(2) Biju, A. T.; Glorius, F. Intermolecular *N*-Heterocyclic Carbene Catalyzed Hydroacylation of Arynes. *Angew. Chem. Int. Ed.* **2010**, *49*, 9761–9764; *Angew. Chem.* **2010**, *122*, 9955–9958.

(3) Zhou, T.; Peters, B.; Maldonado, M. F.; Govender, T.; Andersson, P. G. Enantioselective Synthesis of Chiral Sulfones by Ir-Catalyzed Asymmetric Hydrogenation: A Facile Approach to the Preparation of Chiral Allylic and Homoallylic Compounds. *J. Am. Chem. Soc.* **2012**, *134*, 13592–13595.

(4) Zhang, X.; Wu, J.; Liu, Y.; Xie, Y.; Liu, C.; Wang, J.; Zhao, G. Facile synthetic approaches to 1-thiocyclopropanecarboxylates. *Phosphorus Sulfur Silicon Relat. Elem.* **2017**, *192*, 799–811.

X-ray Crystallographic Details

Single crystals of compounds **1ag**, **1ap**, and **1am** were obtained by slow evaporation with ethanol as the solvent at room temperature. These crystals were selected under a microscope and mounted on the tip of a glass fiber attached to a copper pin. X-ray diffraction data of **1ag**, **1ap**, and **1am** were collected on a Bruker APEX II CCD diffractometer by employment of graphite-monochromated Mo Kα radiation (λ = 0.7107 Å) at 100 K in ω scan mode. Their space groups were determined on the basis of systematic absences and intensity statistics, and the structures were solved by direct methods and refined with SHELXL-2014/6. Empirical absorption corrections were applied by use of multiscans. All non-hydrogen atoms were refined with anisotropic displacement factors, while hydrogen atoms were placed in ideal positions and fixed with relative isotropic displacement parameters. Detailed crystallographic data are provided in the CIF files.

X-ray Crystal Data for Compound 1ag

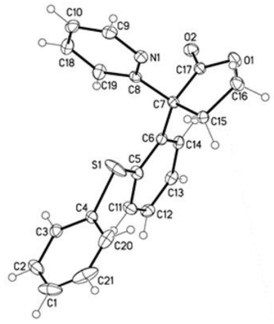

Figure S1. ORTEP diagram of α,α-diarylated lactone **1ag**, as determined by X-ray analysis (ellipsoid contour probability 50%).

Table S2. Crystal data and structure refinement for **1ag**.

Identification code	mo_160319lt_0m
Empirical formula	C21 H17 N O2 S

Formula weight	347.42	
Temperature	100(2) K	
Wavelength	0.71073 Å	
Crystal system	Triclinic	
Space group	P -1	
Unit cell dimensions	a = 7.9281(5) Å	α = 91.7930(10)°.
	b = 9.2566(6) Å	β = 99.0670(10)°.
	c = 11.5548(7) Å	γ = 98.9810(10)°.
Volume	825.75(9) Å ^{3}	
Z	2	
Density (calculated)	1.397 Mg/m^3	
Absorption coefficient	0.210 mm^{-1}	
F(000)	364	
Crystal size	0.17 x 0.13 x 0.10 mm^3	
Theta range for data collection	1.788 to 26.442°.	
Index ranges	-9<=h<=9, -11<=k<=11, -14<=l<=14	
Reflections collected	12141	
Independent reflections	3318 [R(int) = 0.0191]	
Completeness to theta = 25.242°	98.1 %	
Absorption correction	Semi-empirical from equivalents	
Max. and min. transmission	0.9485 and 0.8900	
Refinement method	Full-matrix least-squares on F^2	
Data / restraints / parameters	3318 / 0 / 226	
Goodness-of-fit on F^2	1.040	
Final R indices [I>2sigma(I)]	R1 = 0.0323, wR2 = 0.0752	

R indices (all data)	R1 = 0.0377, wR2 = 0.0785
Extinction coefficient	n/a
Largest diff. peak and hole	0.319 and -0.398 e.Å $^{-3}$

X-ray Crystal Data for Compound 1ap

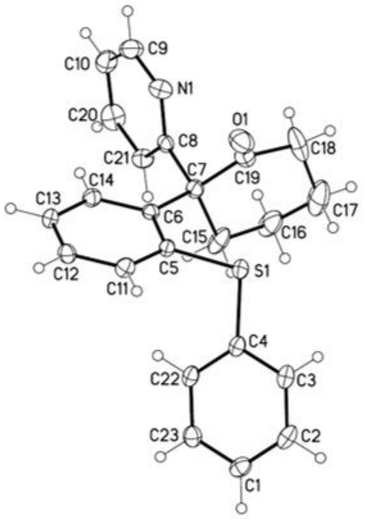

Figure S2. ORTEP diagram of α,α-diarylated cyclohexanone **1ap**, as determined by X-ray analysis (ellipsoid contour probability 50%).

Table S3. Crystal data and structure refinement for **1ap**.

Identification code	160643LT	
Empirical formula	C23 H21 N O S	
Formula weight	359.47	
Temperature	100(2) K	
Wavelength	0.71073 Å	
Crystal system	Monoclinic	
Space group	P 21/c	
Unit cell dimensions	a = 16.4567(4) Å	α = 90°.
	b = 7.9050(2) Å	β = 111.8510(10)°.

	c = 14.9464(3) Å	γ = 90°.
Volume	1804.69(7) $Å^3$	
Z	4	
Density (calculated)	1.323 Mg/m^3	
Absorption coefficient	0.191 mm^{-1}	
F(000)	760	
Crystal size	0.12 x 0.08 x 0.03 mm^3	
Theta range for data collection	1.333 to 26.365°.	
Index ranges	-20<=h<=20, -9<=k<=9, -18<=l<=18	
Reflections collected	15406	
Independent reflections	3679 [R(int) = 0.0294]	
Completeness to theta = 25.242°	100.0 %	
Absorption correction	Semi-empirical from equivalents	
Max. and min. transmission	0.9485 and 0.9003	
Refinement method	Full-matrix least-squares on F^2	
Data / restraints / parameters	3679 / 0 / 235	
Goodness-of-fit on F^2	1.037	
Final R indices [I>2sigma(I)]	R1 = 0.0520, wR2 = 0.1341	
R indices (all data)	R1 = 0.0629, wR2 = 0.1428	
Extinction coefficient	n/a	
Largest diff. peak and hole	1.741 and -0.402 e.$Å^{-3}$	

X-ray Crystal Data for Compound 1am

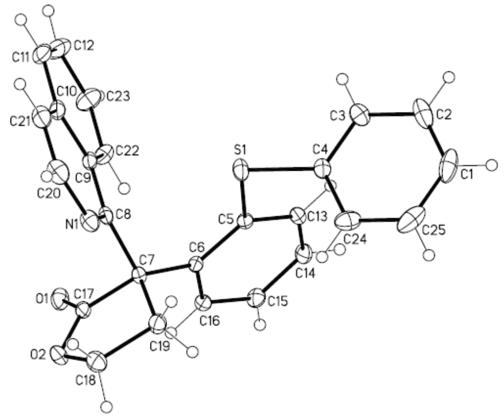

Figure S3. ORTEP diagram of α,α-diarylated lactone **1am**, as determined by X-ray analysis (ellipsoid contour probability 50%).

Table S4. Crystal data and structure refinement for **1am**.

Identification code	170244LT	
Empirical formula	C25 H19 N O2 S	
Formula weight	397.47	
Temperature	100(2) K	
Wavelength	0.71073 Å	
Crystal system	Triclinic	
Space group	P -1	
Unit cell dimensions	a = 9.3348(9) Å	α = 95.481(2)°.
	b = 9.3427(9) Å	β = 104.304(2)°.
	c = 11.5909(11) Å	γ = 102.138(2)°.
Volume	945.96(16) Å ^{3}	
Z	2	
Density (calculated)	1.395 Mg/m^3	
Absorption coefficient	0.194 mm^{-1}	

F(000)	416
Crystal size	0.22 x 0.20 x 0.20 mm^3
Theta range for data collection	1.836 to 28.349°.
Index ranges	-12<=h<=11, -12<=k<=11, -15<=l<=15
Reflections collected	16597
Independent reflections	4720 [R(int) = 0.0204]
Completeness to theta = 25.242°	99.7 %
Absorption correction	Semi-empirical from equivalents
Max. and min. transmission	0.9485 and 0.9199
Refinement method	Full-matrix least-squares on F^2
Data / restraints / parameters	4720 / 0 / 262
Goodness-of-fit on F^2	1.047
Final R indices [I>2sigma(I)]	R1 = 0.0332, wR2 = 0.0841
R indices (all data)	R1 = 0.0374, wR2 = 0.0874
Extinction coefficient	n/a
Largest diff. peak and hole	0.372 and -0.294 e.$Å^{-3}$

^{1}H NMR, ^{13}C{^{1}H} NMR, and IR Spectra of New Compounds

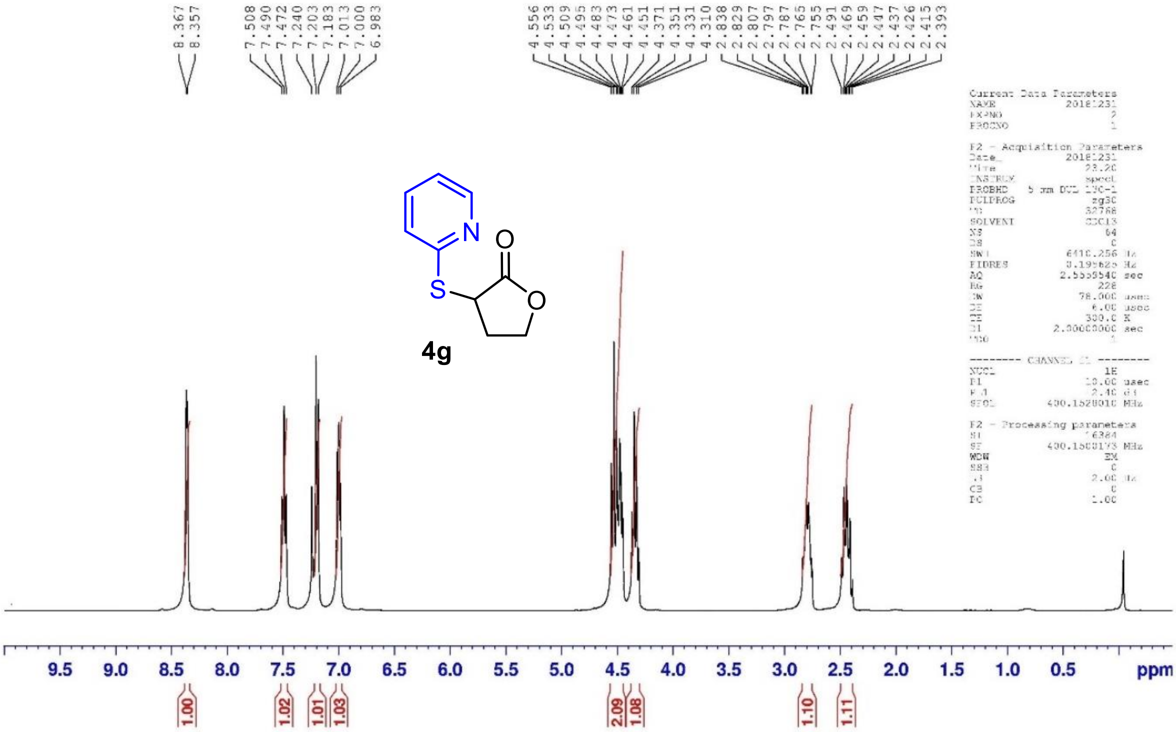

^{1}H NMR (400 MHz, $CDCl_3$) spectrum of compound **4g**

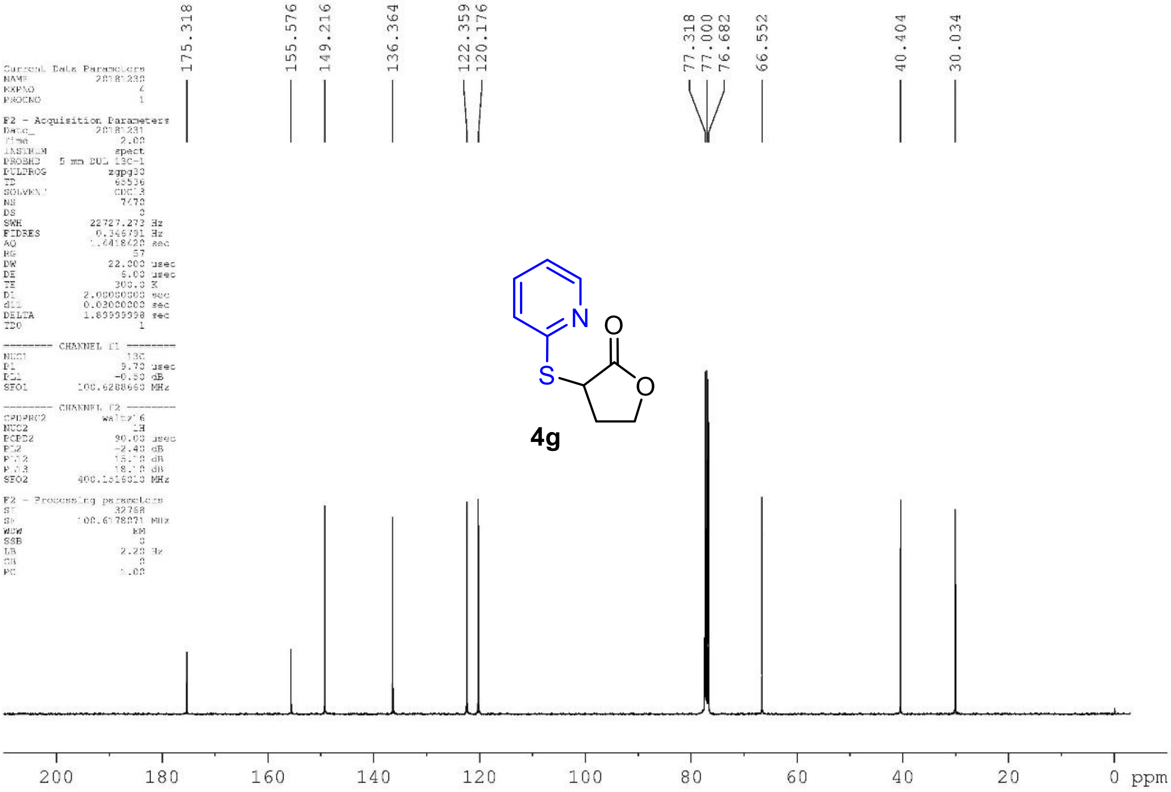

^{13}C{^{1}H} NMR (100 MHz, $CDCl_3$) spectrum of compound **4g**

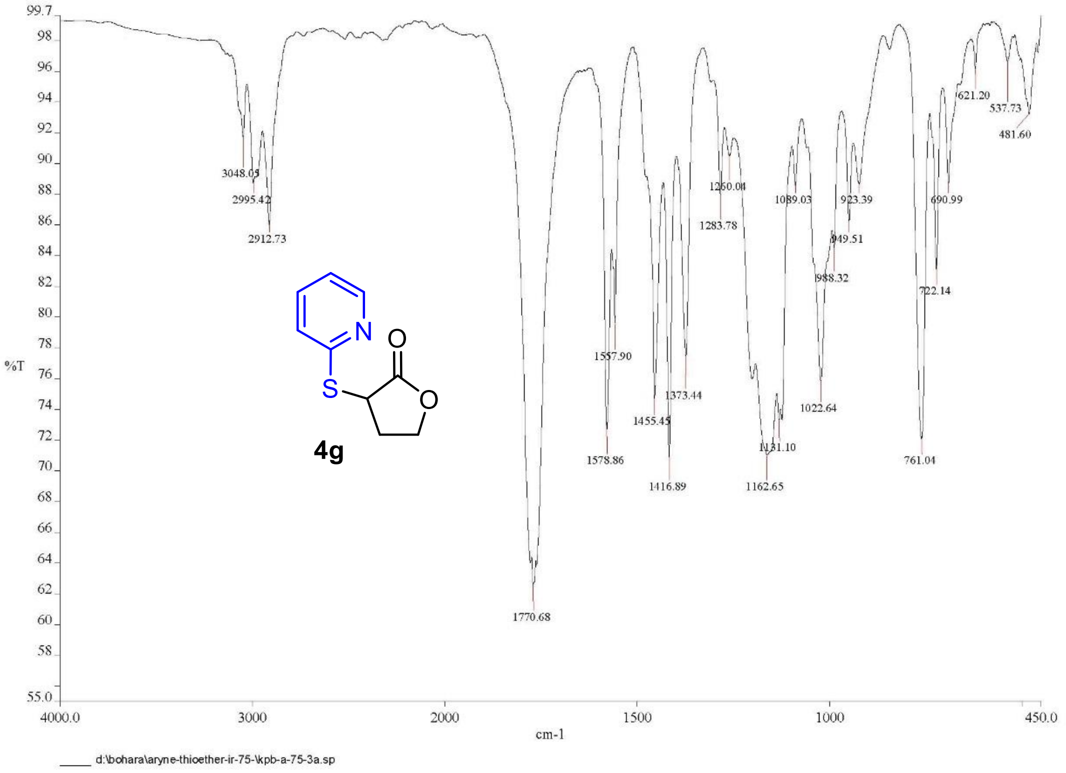

IR spectrum of compound **4g**

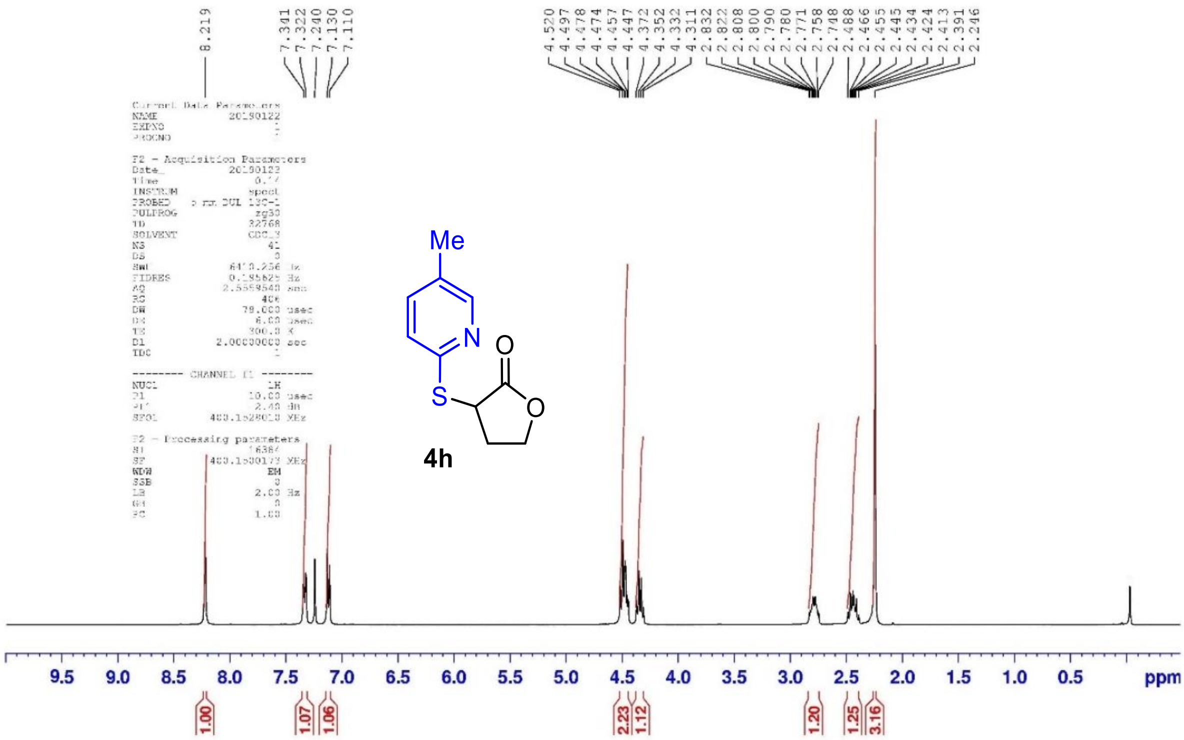

^{1}H NMR (400 MHz, $CDCl_3$) spectrum of compound **4h**

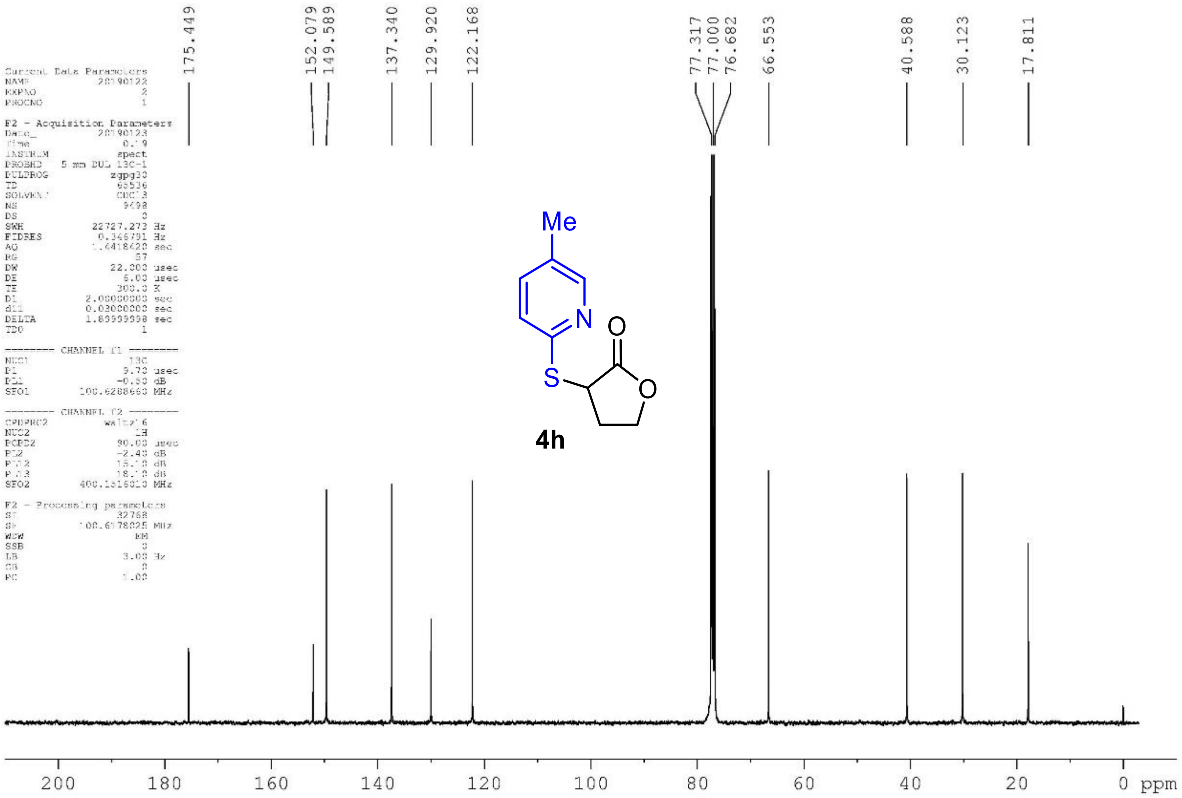

$^{13}C\{^{1}H\}$ NMR (100 MHz, $CDCl_3$) spectrum of compound **4h**

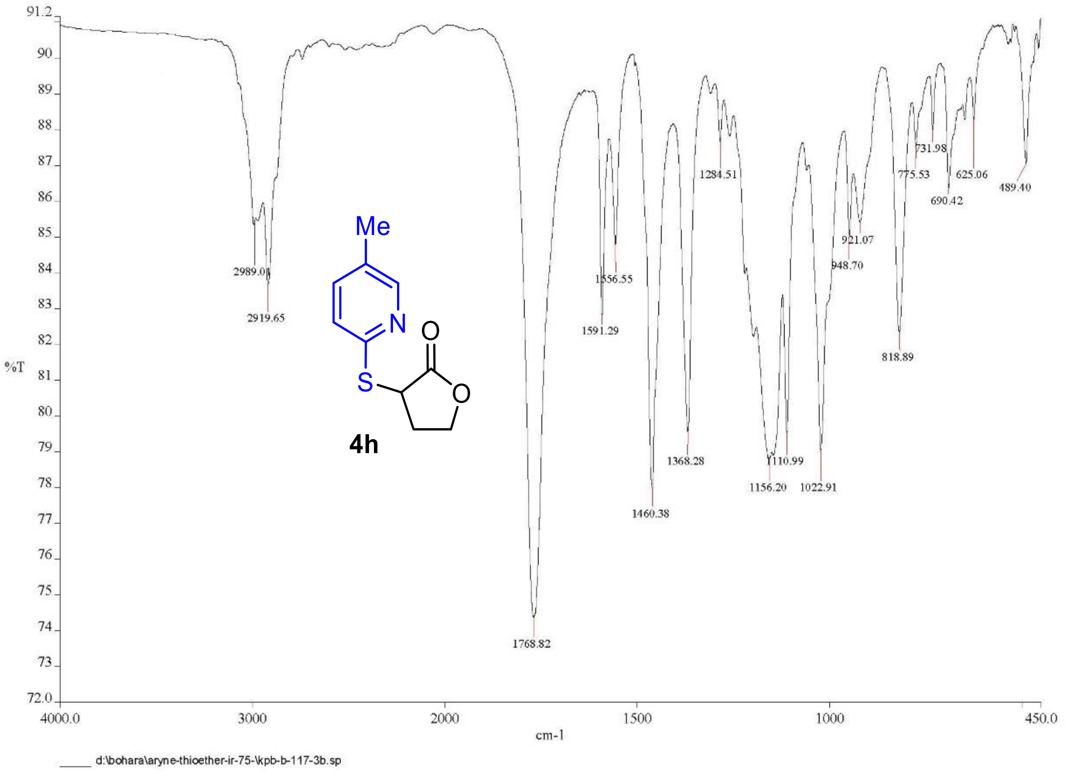

IR spectrum of compound **4h**

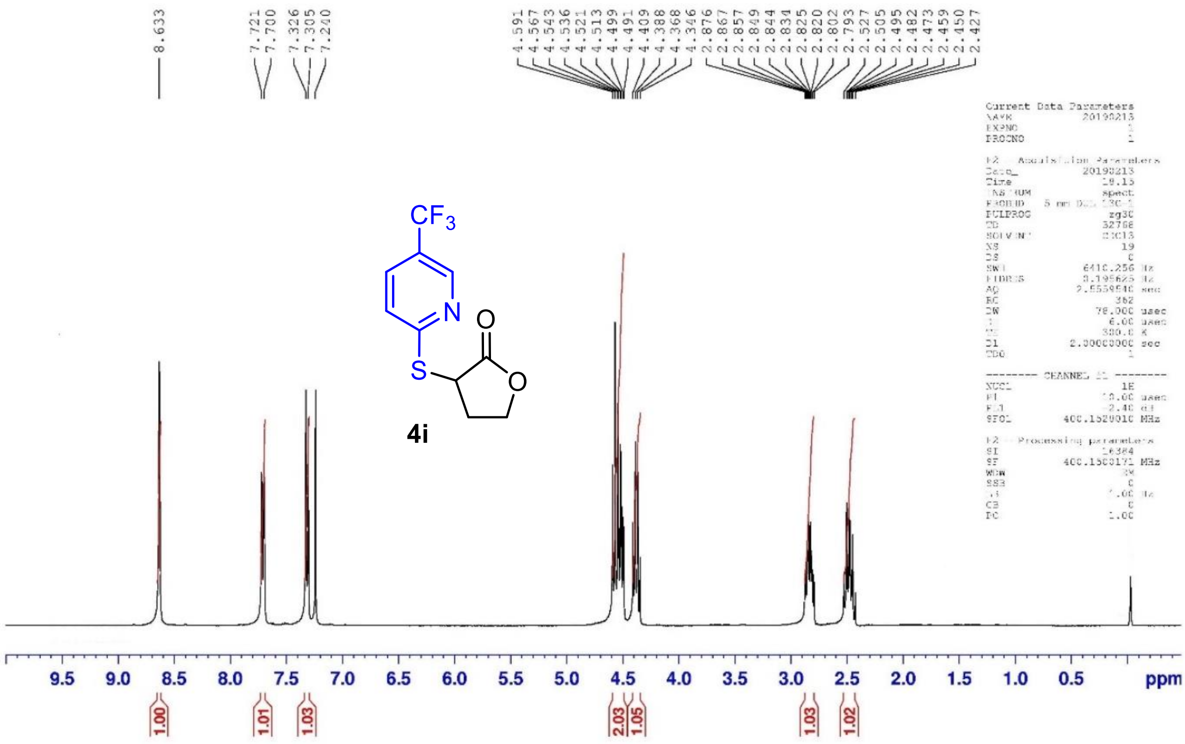

^{1}H NMR (400 MHz, $CDCl_3$) spectrum of compound **4i**

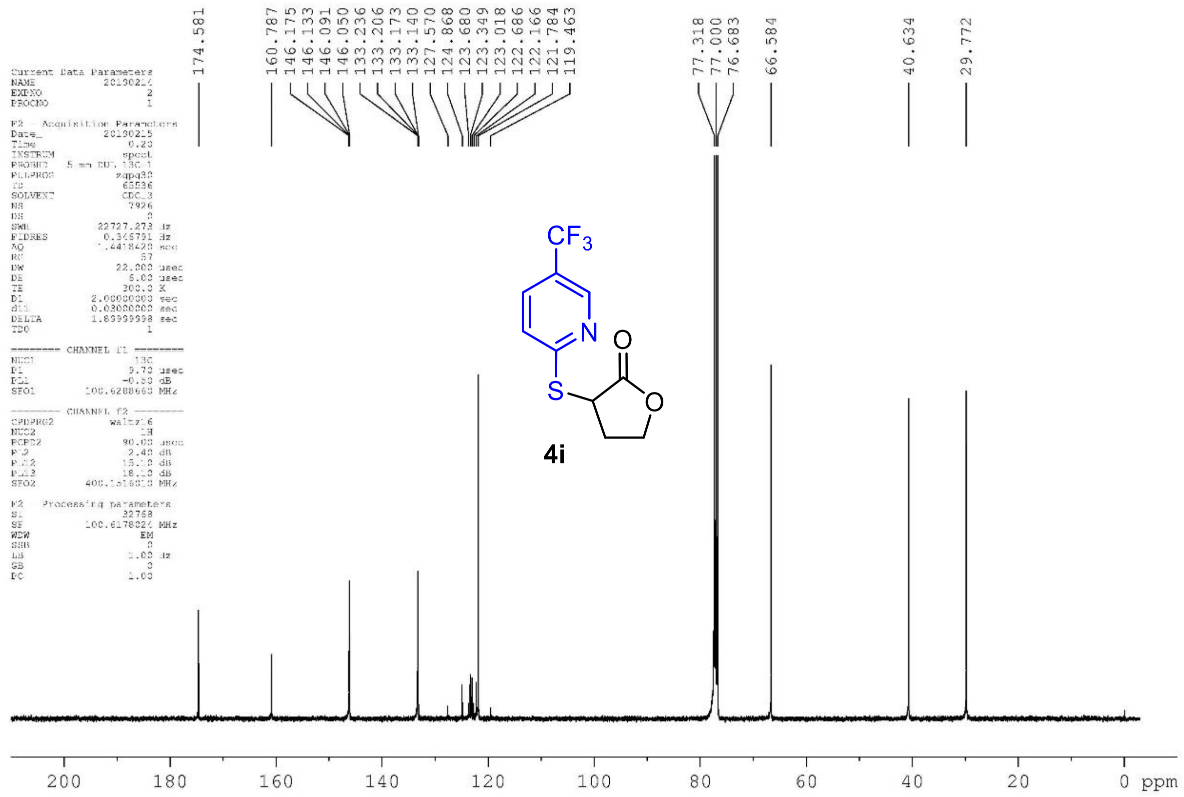

$^{13}C\{^{1}H\}$ NMR (100 MHz, $CDCl_3$) spectrum of compound **4i**

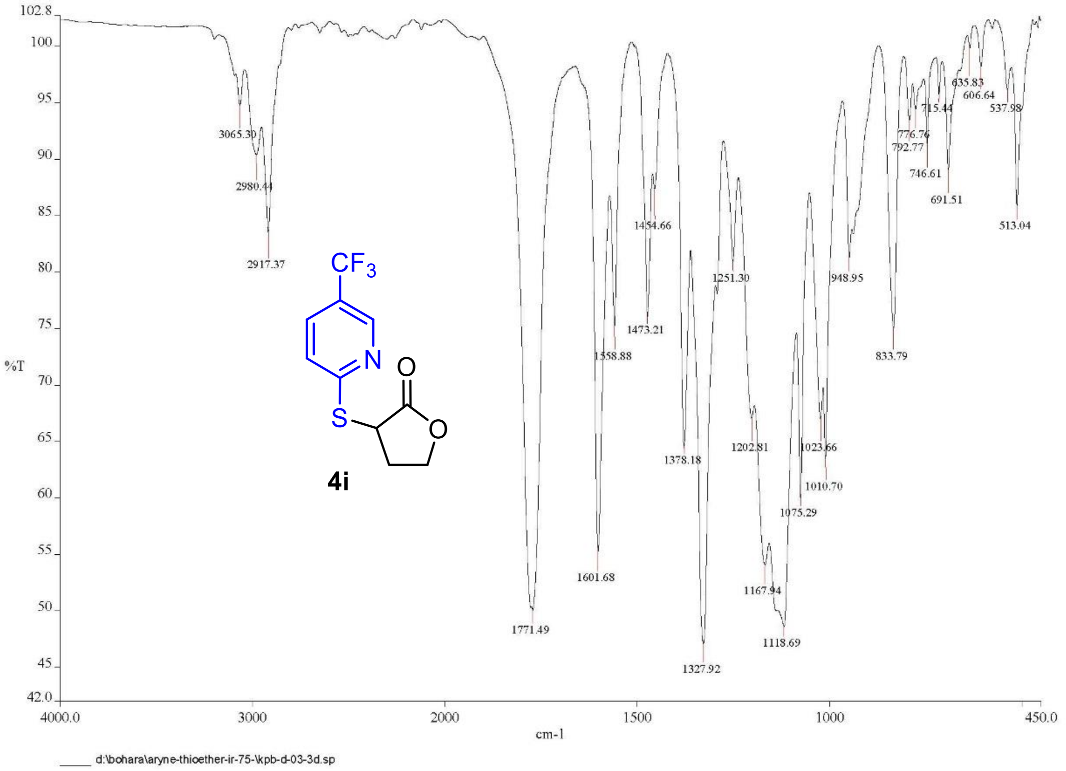

IR spectrum of compound **4i**

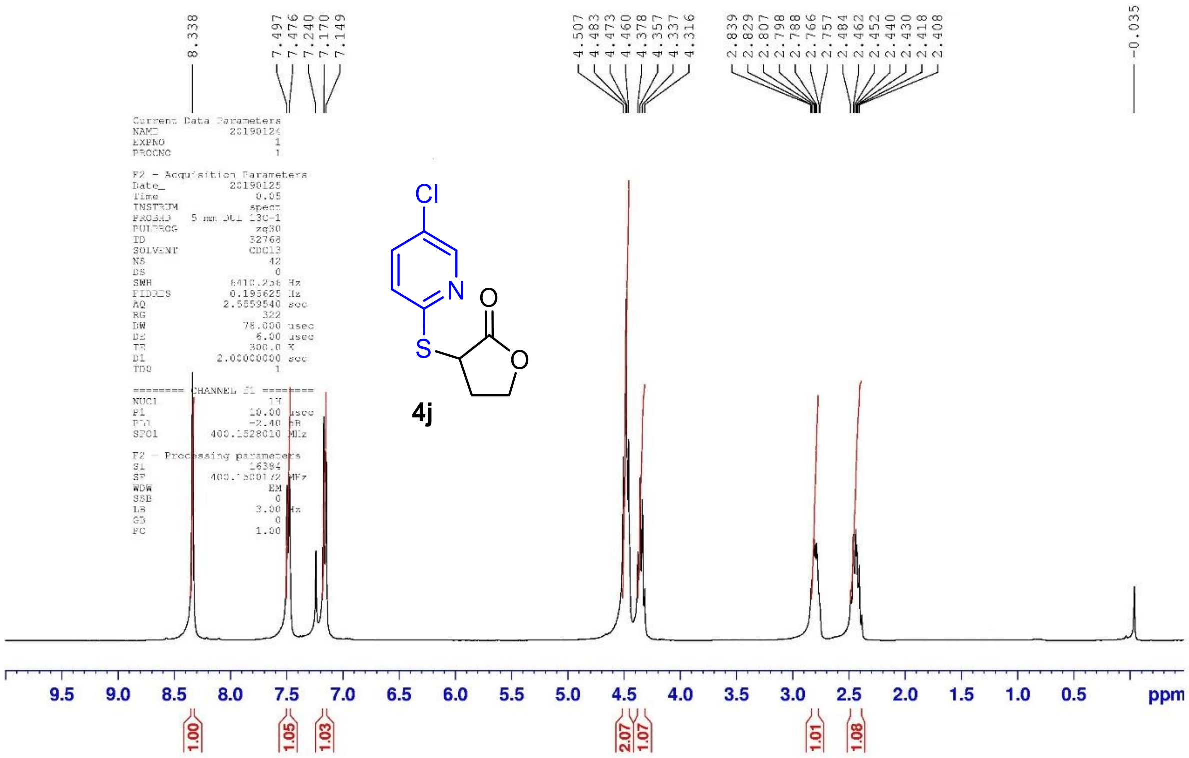

^{1}H NMR (400 MHz, $CDCl_3$) spectrum of compound **4j**

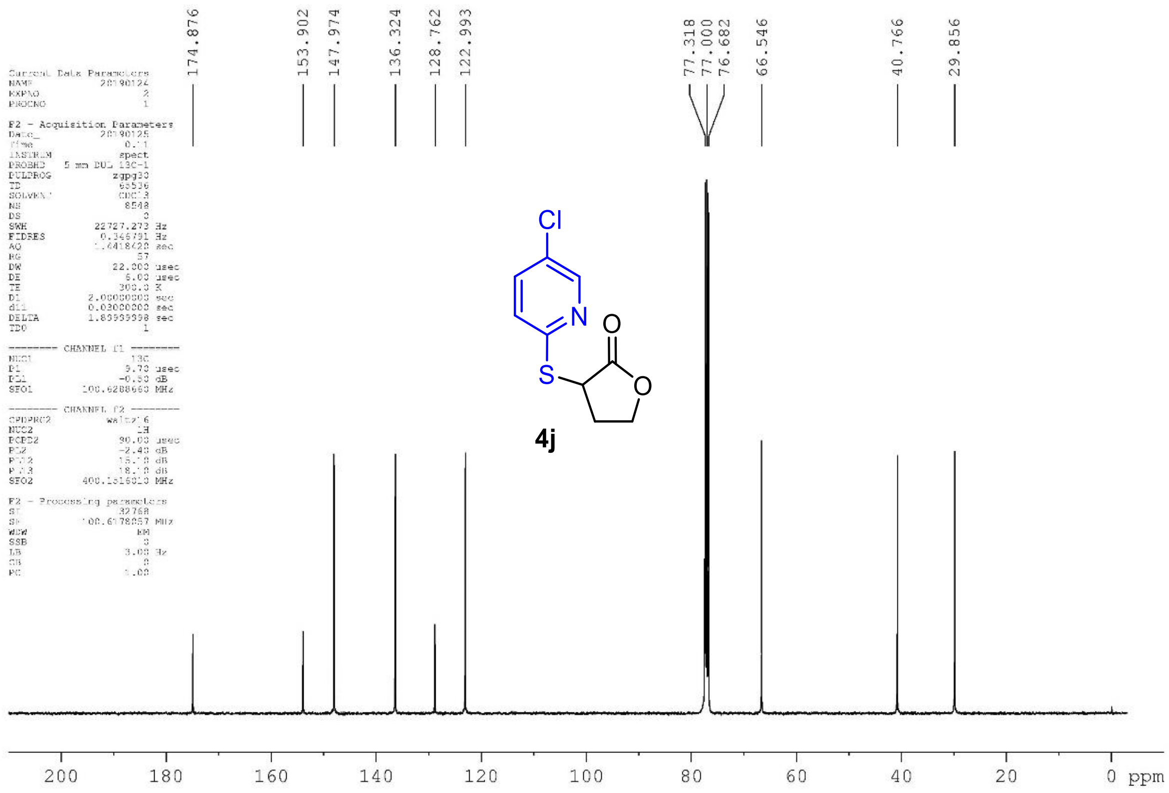

$^{13}C\{^{1}H\}$ NMR (100 MHz, $CDCl_3$) spectrum of compound **4j**

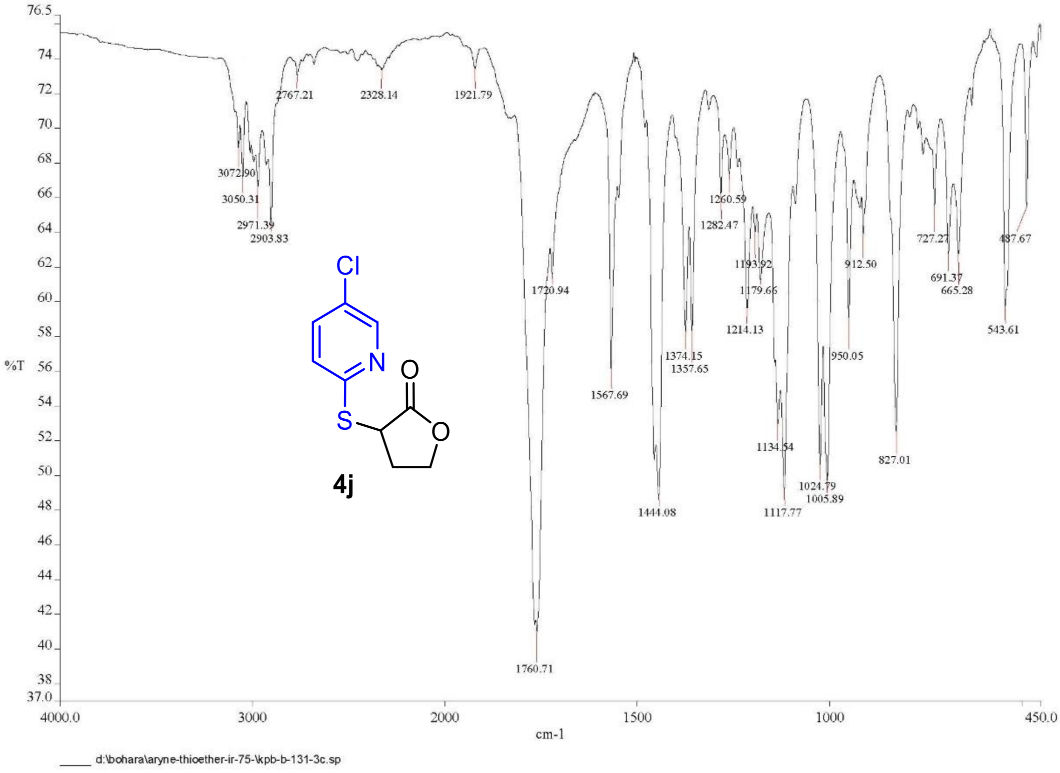

IR spectrum of compound **4j**

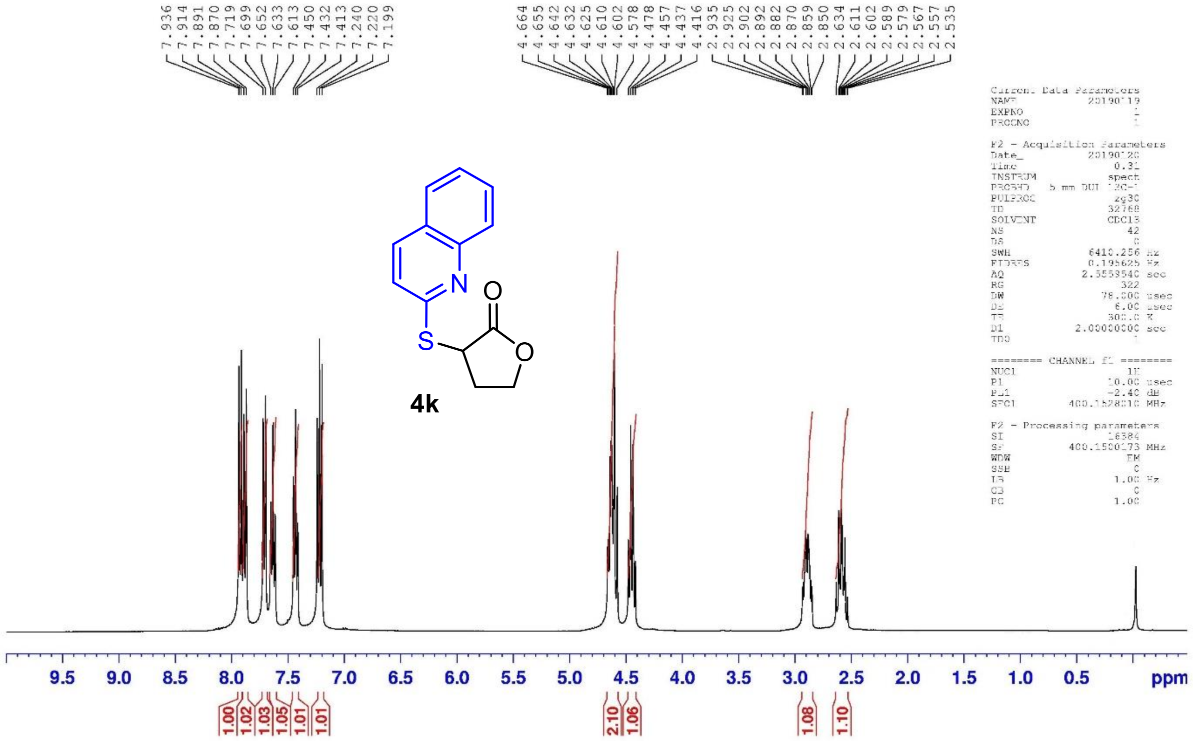

^{1}H NMR (400 MHz, $CDCl_3$) spectrum of compound **4k**

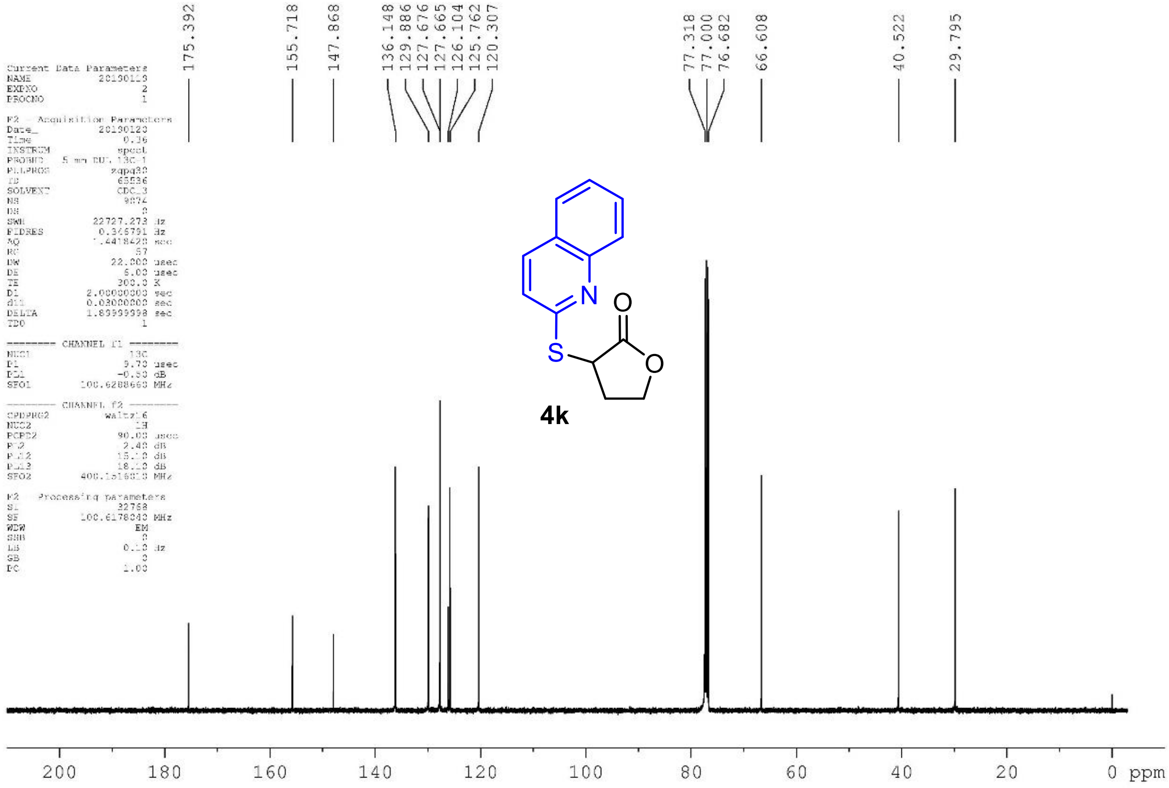

^{13}C{^{1}H} NMR (100 MHz, $CDCl_3$) spectrum of compound **4k**

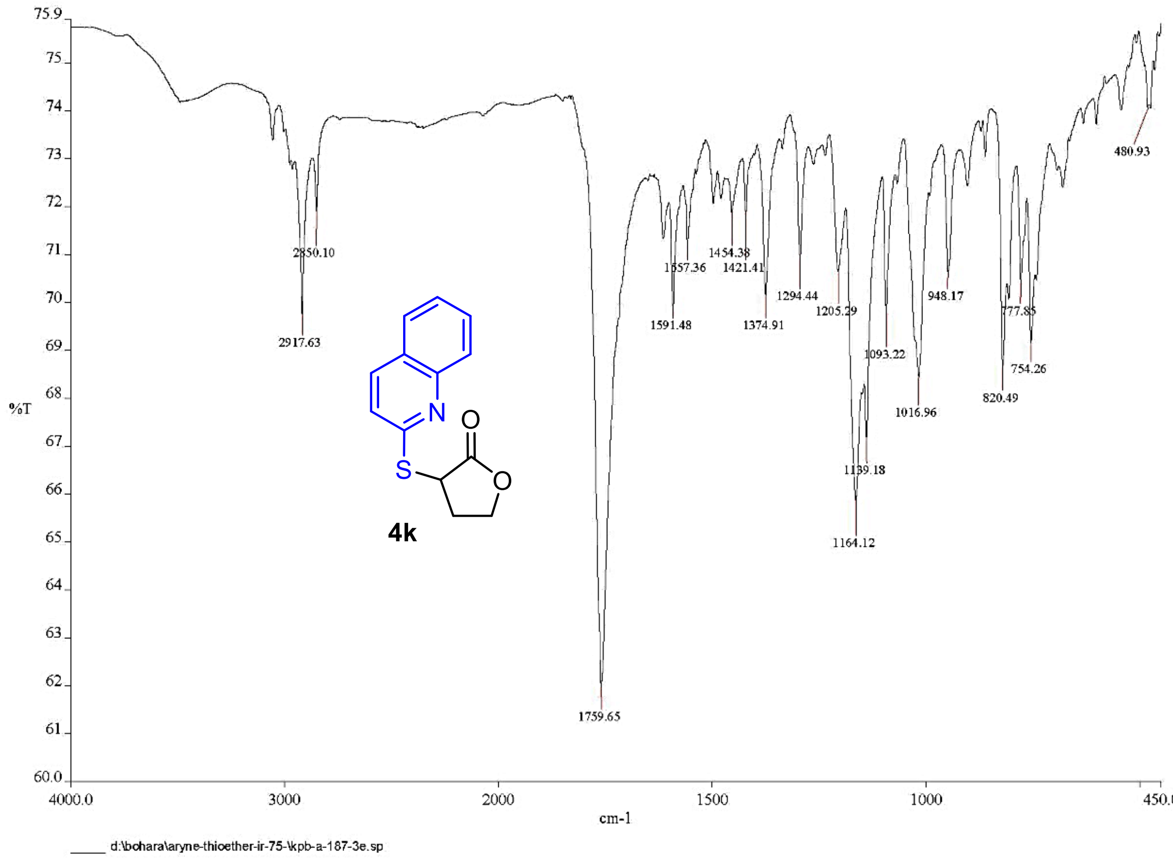

IR spectrum of compound **4k**

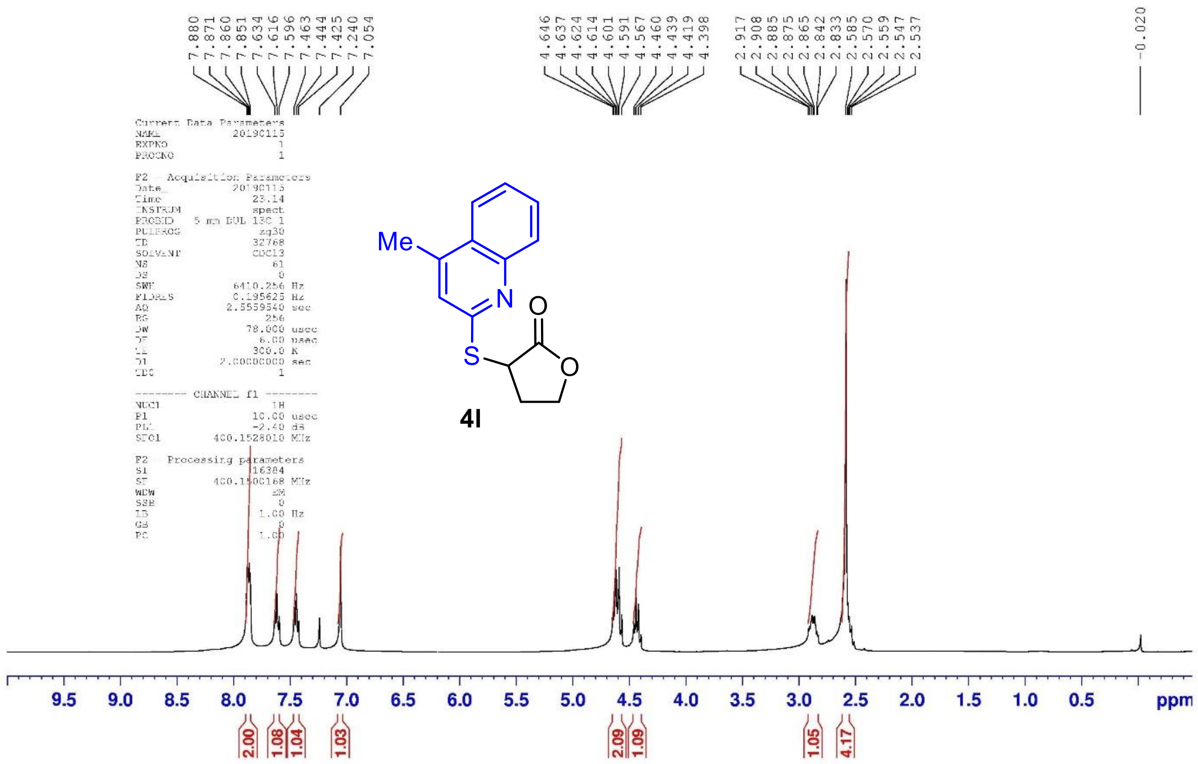

^{1}H NMR (400 MHz, $CDCl_3$) spectrum of compound **4l**

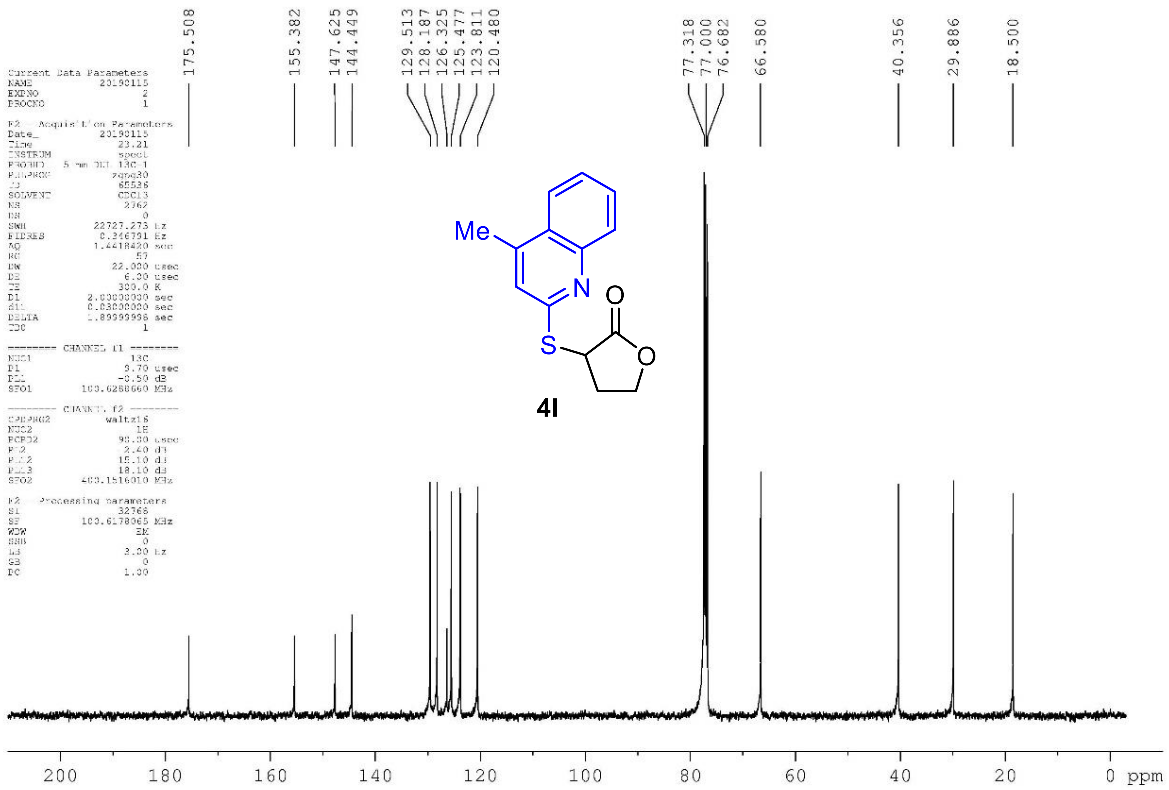

$^{13}C\{^1H\}$ NMR (100 MHz, $CDCl_3$) spectrum of compound **4l**

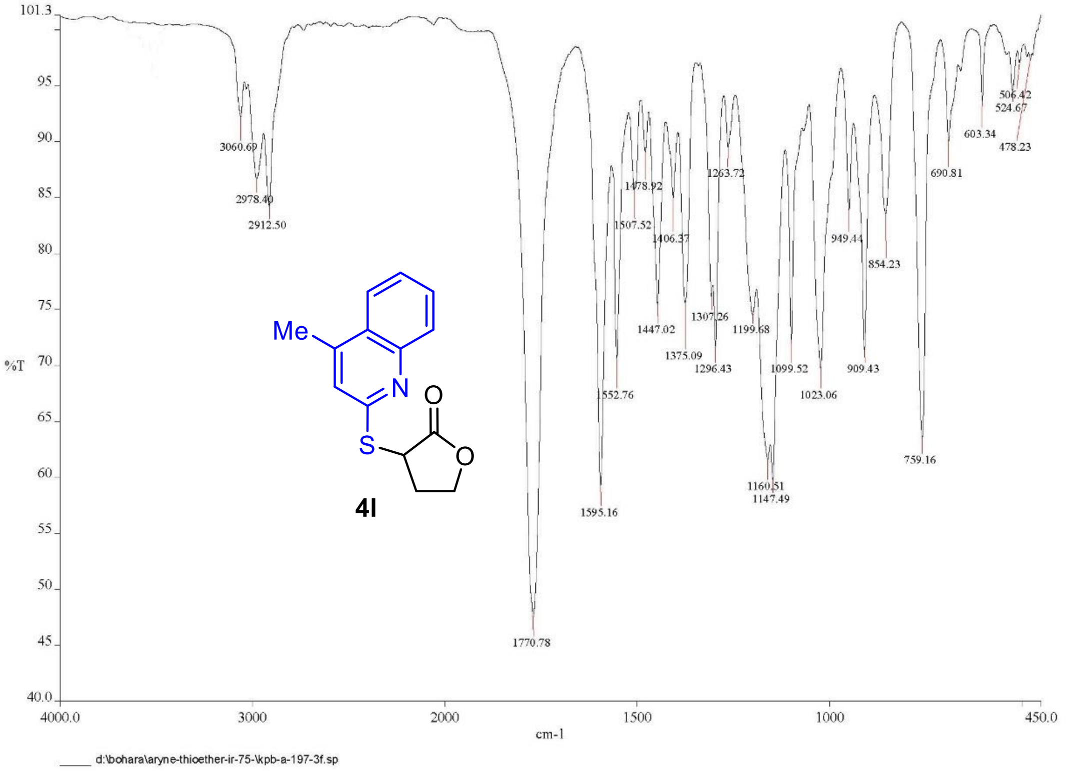

IR spectrum of compound **4l**

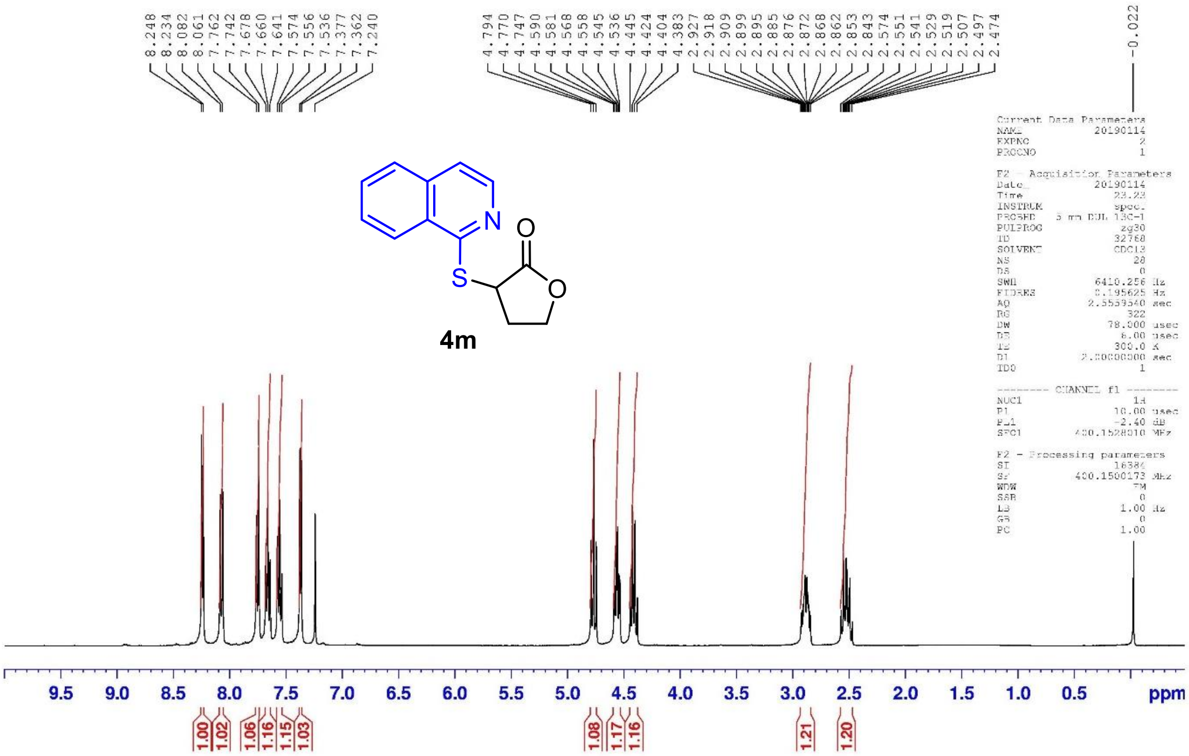

^{1}H NMR (400 MHz, CDCl$_3$) spectrum of compound **4m**

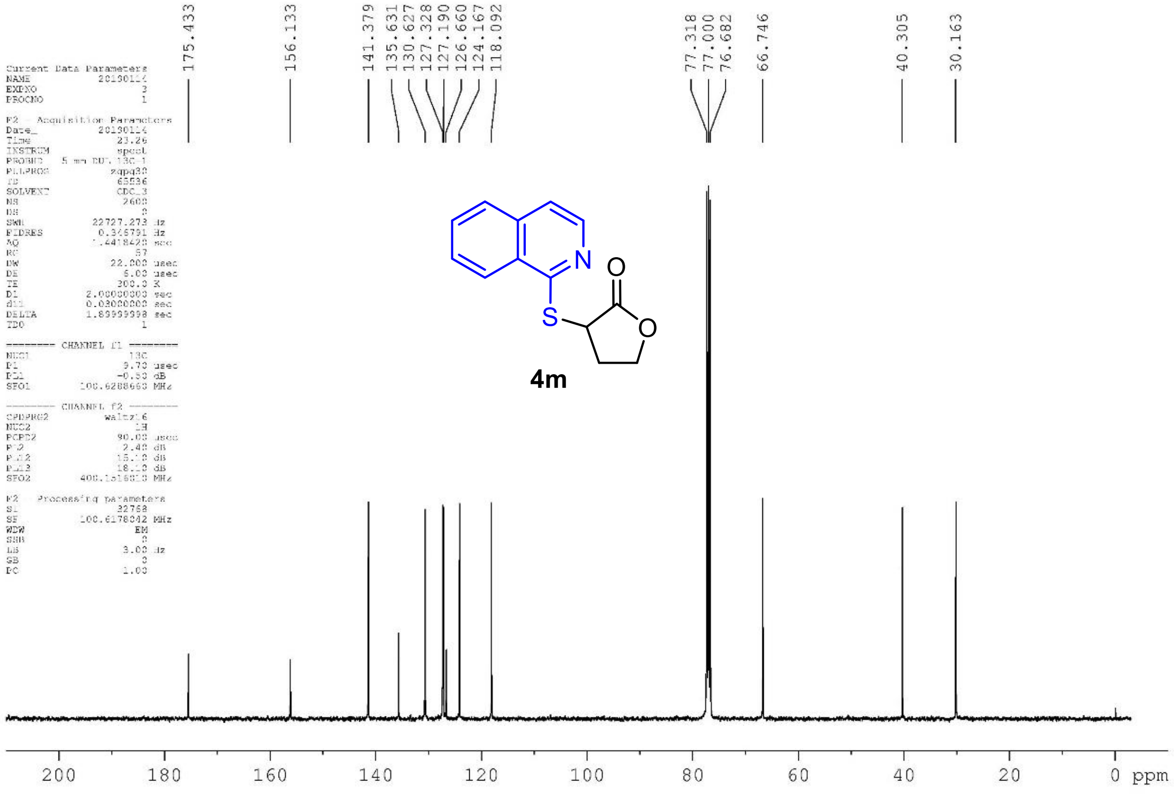

^{13}C{^{1}H} NMR (100 MHz, CDCl$_3$) spectrum of compound **4m**

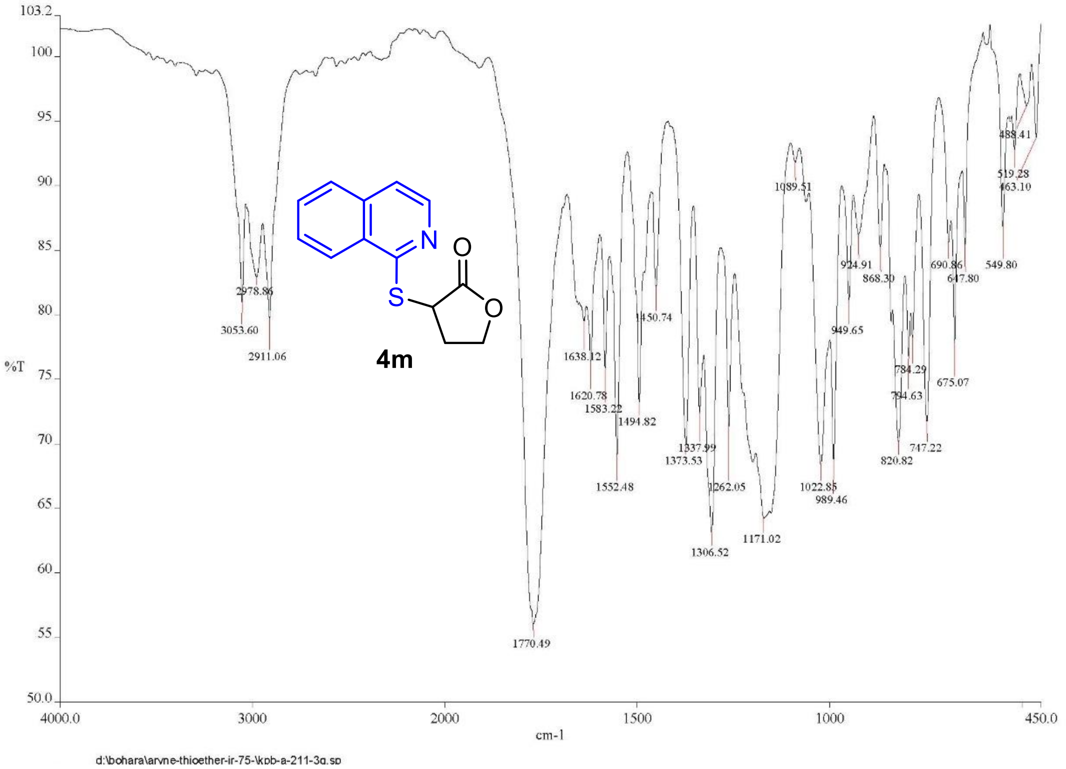

IR spectrum of compound **4m**

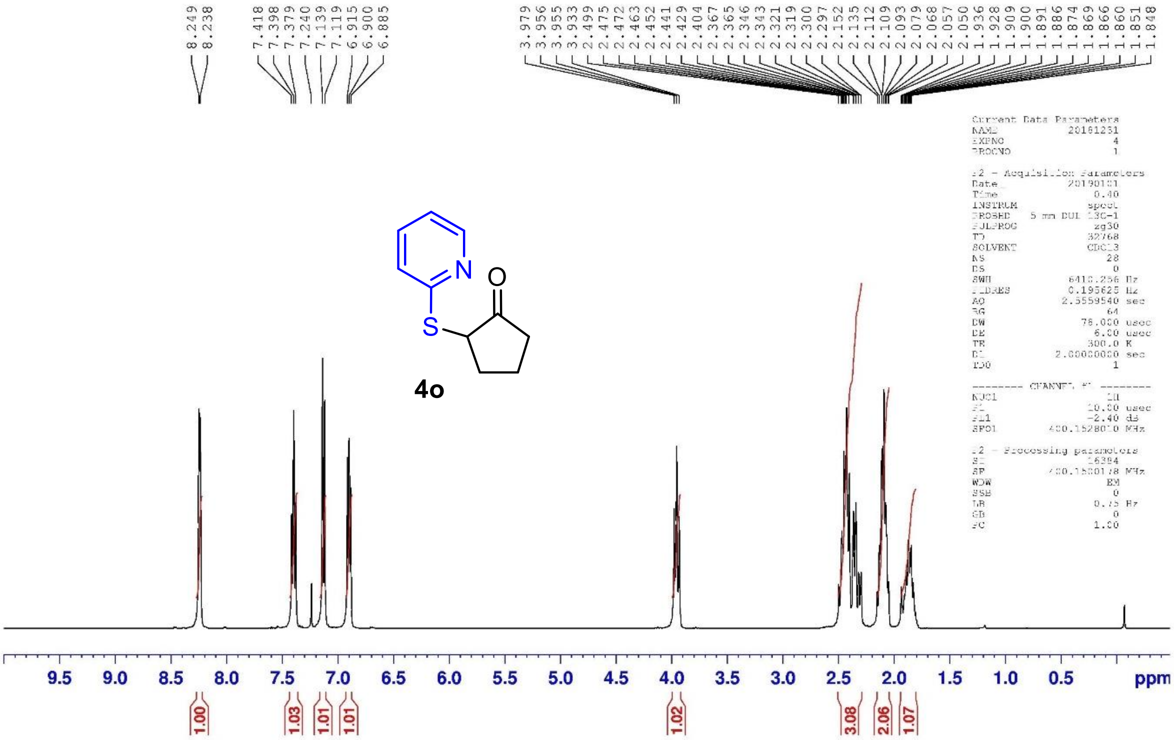

^{1}H NMR (400 MHz, $CDCl_3$) spectrum of compound **4o**

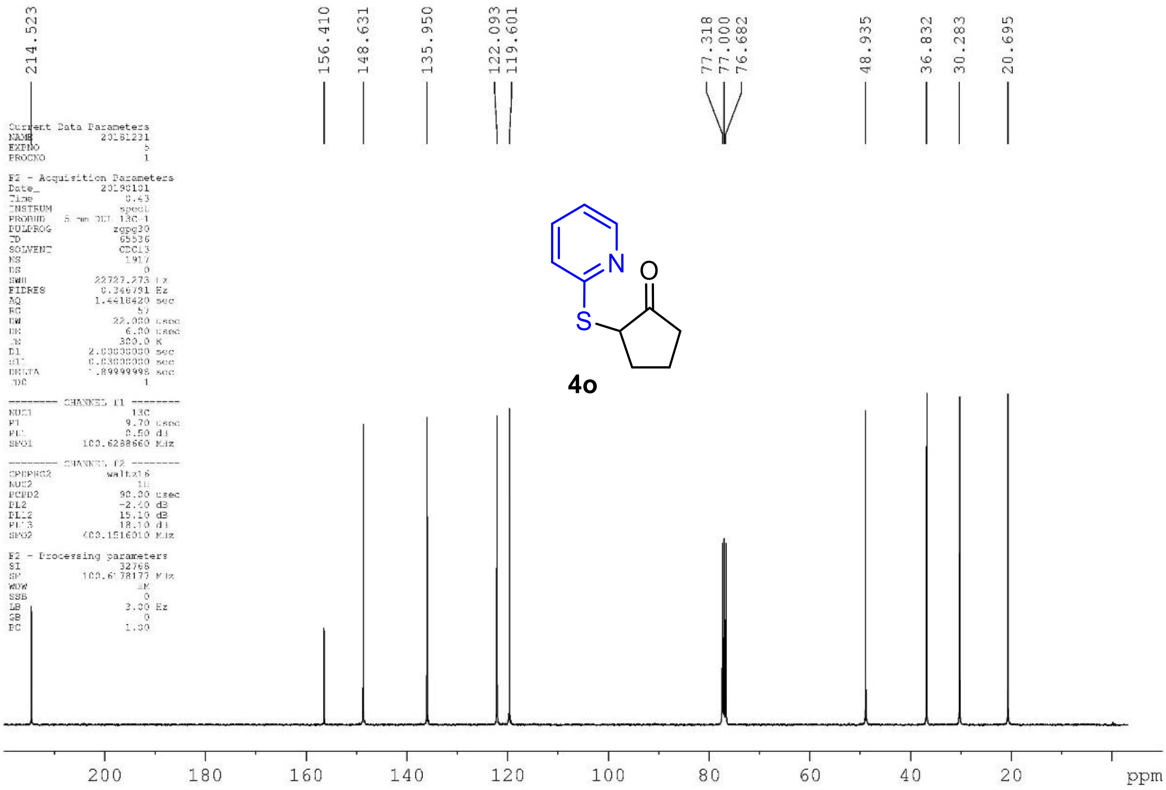

$^{13}C\{^{1}H\}$ NMR (100 MHz, $CDCl_3$) spectrum of compound **4o**

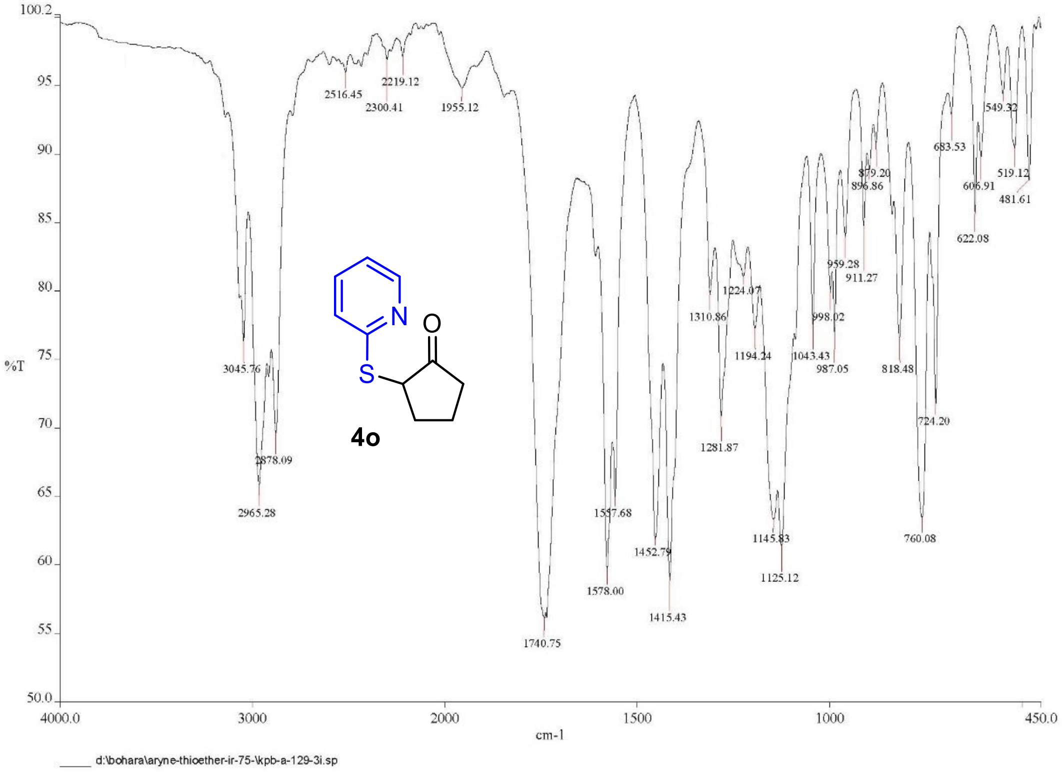

IR spectrum of compound **4o**

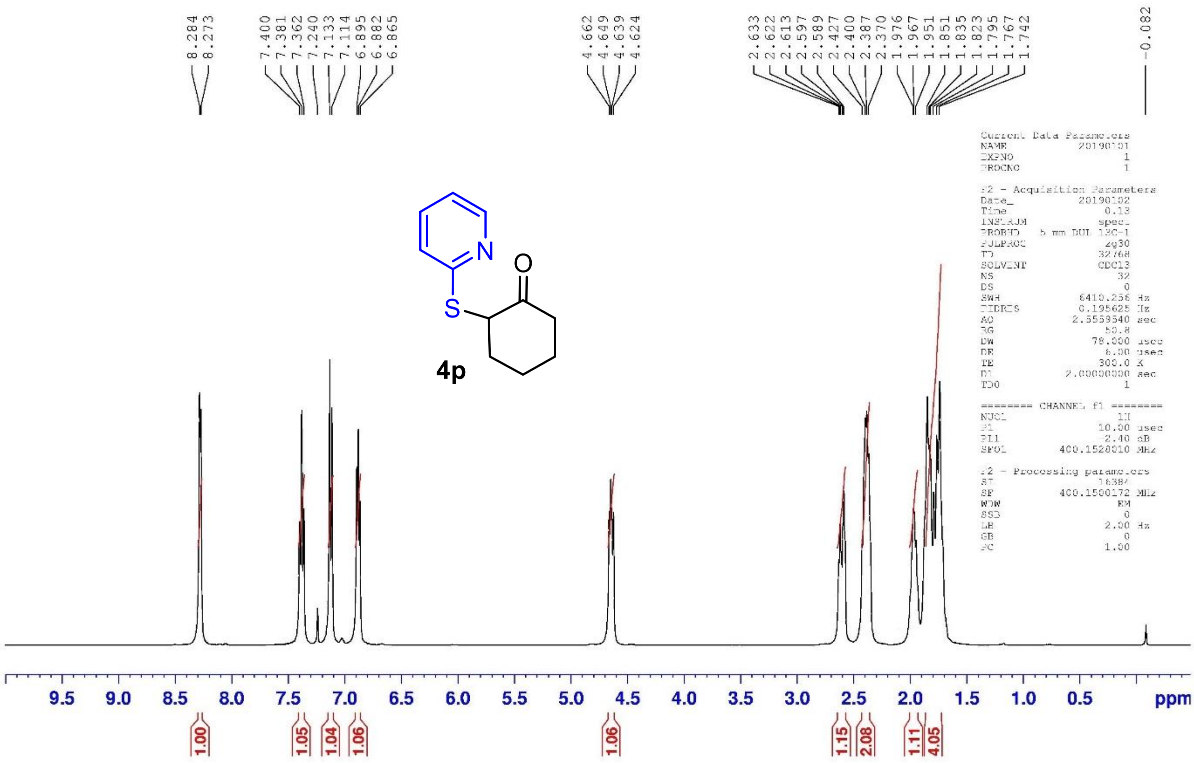

^{1}H NMR (400 MHz, $CDCl_3$) spectrum of compound **4p**

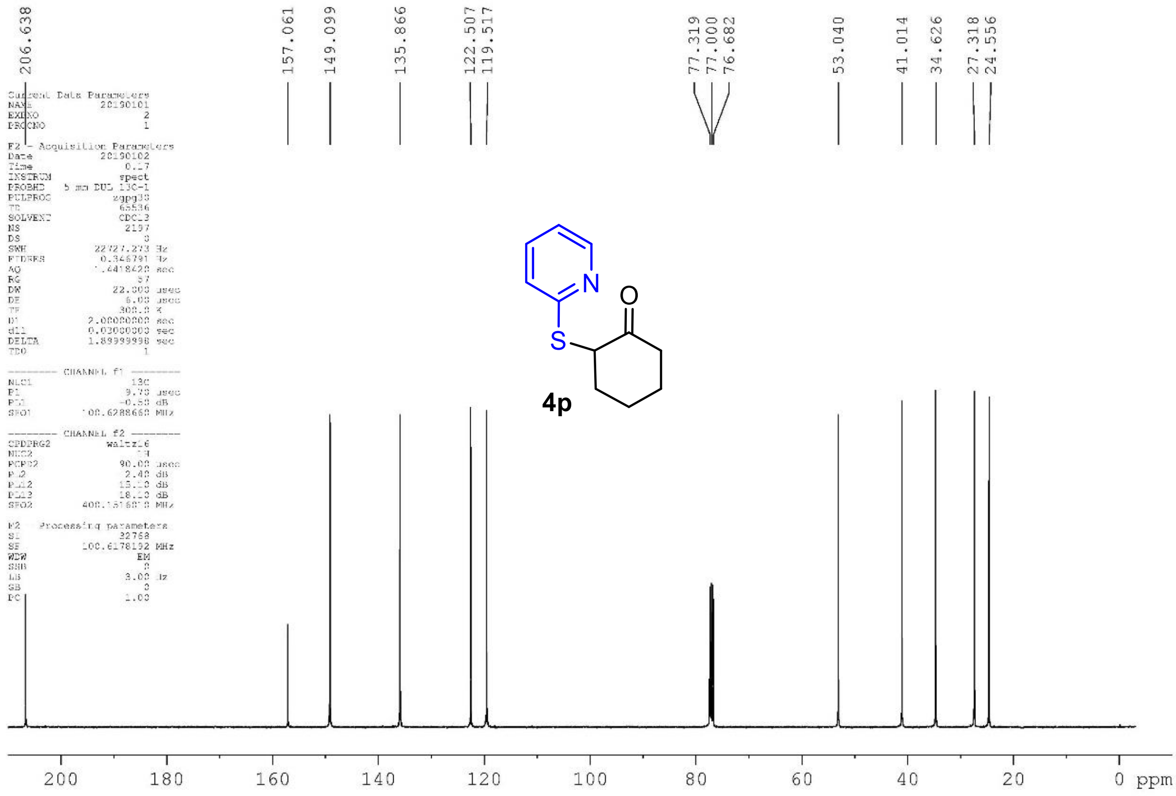

^{13}C{^{1}H} NMR (100 MHz, $CDCl_3$) spectrum of compound **4p**

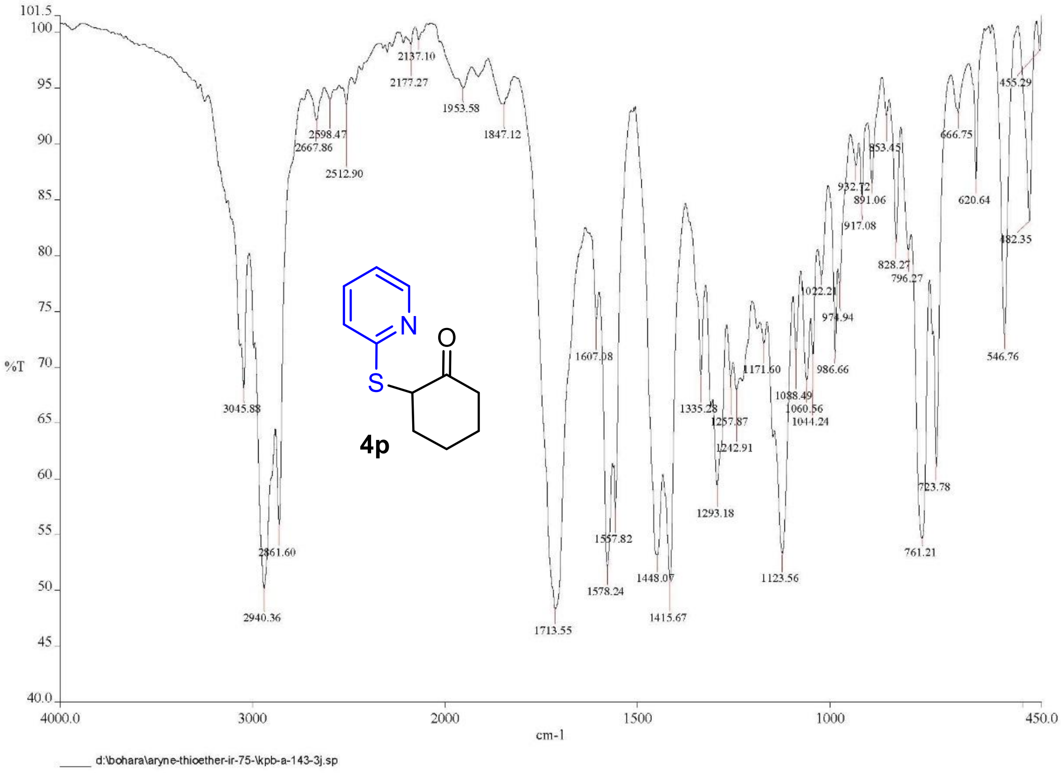

IR spectrum of compound **4p**

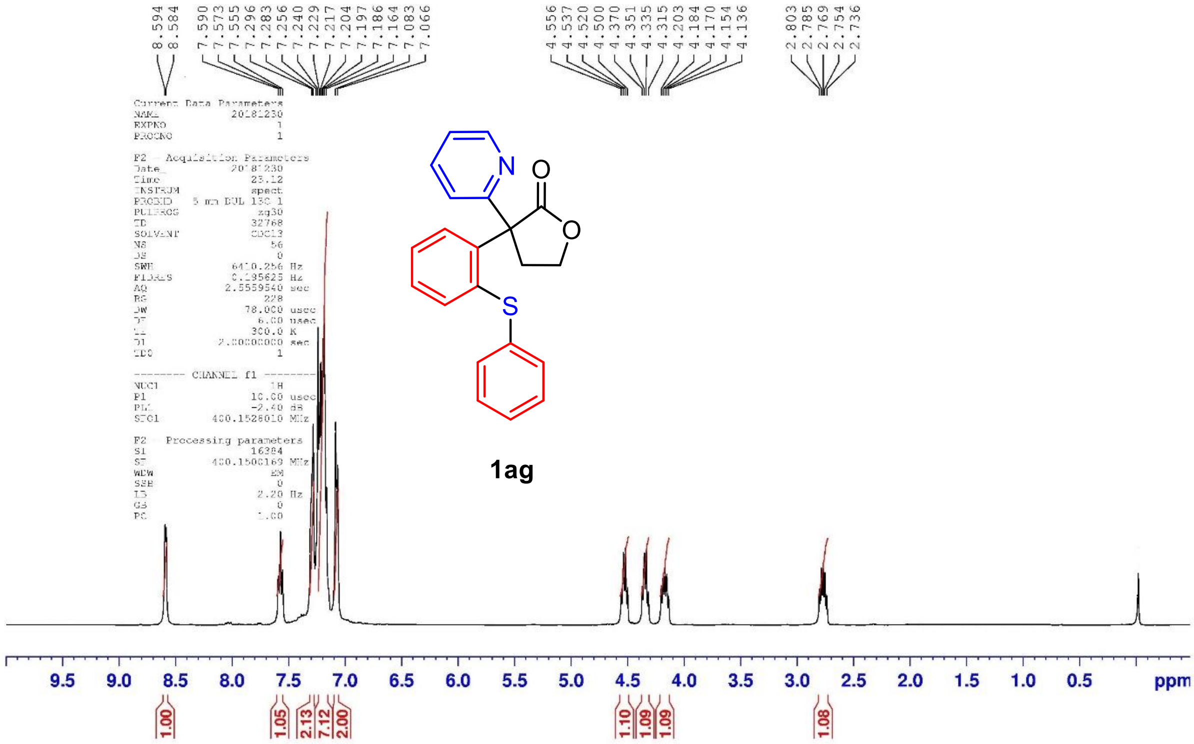

^{1}H NMR (400 MHz, $CDCl_3$) spectrum of compound **1ag**

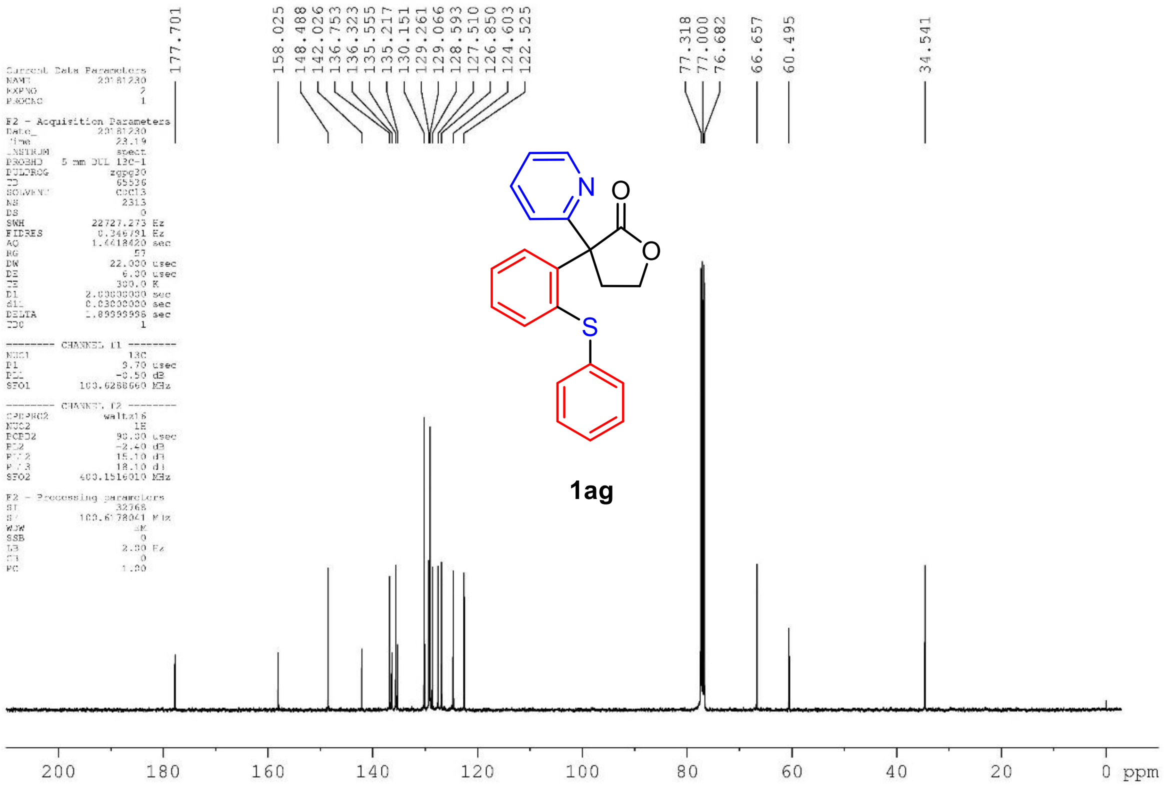

$^{13}C\{^{1}H\}$ NMR (100 MHz, $CDCl_3$) spectrum of compound **1ag**

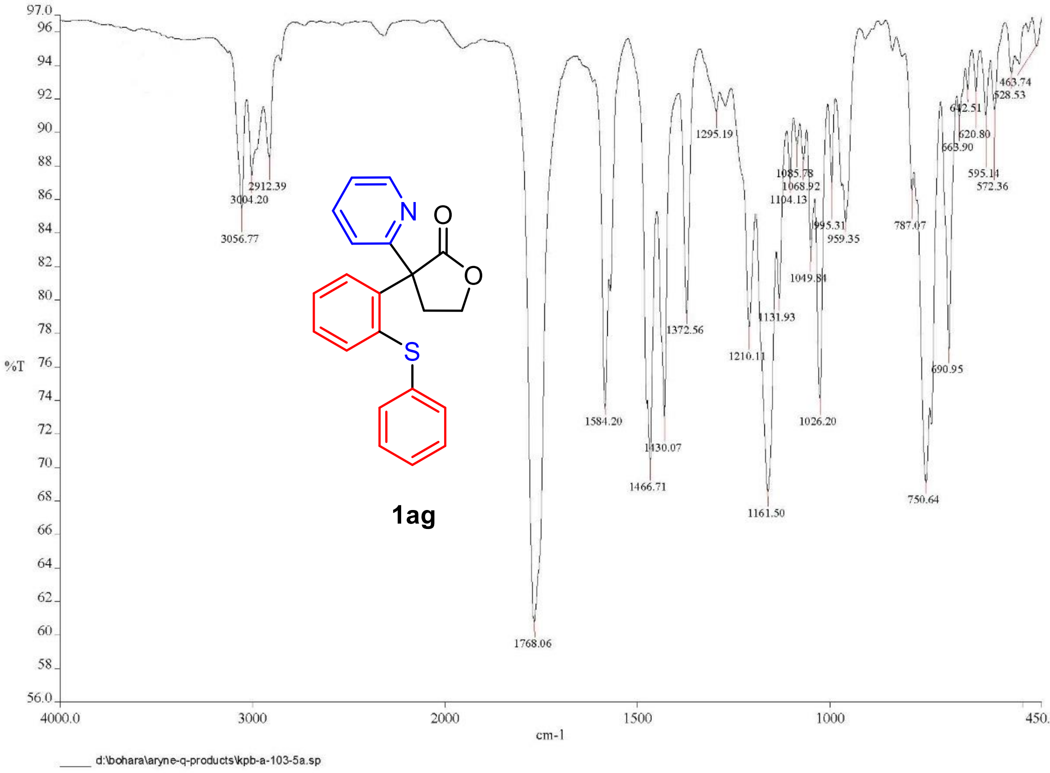

IR spectrum of compound **1ag**

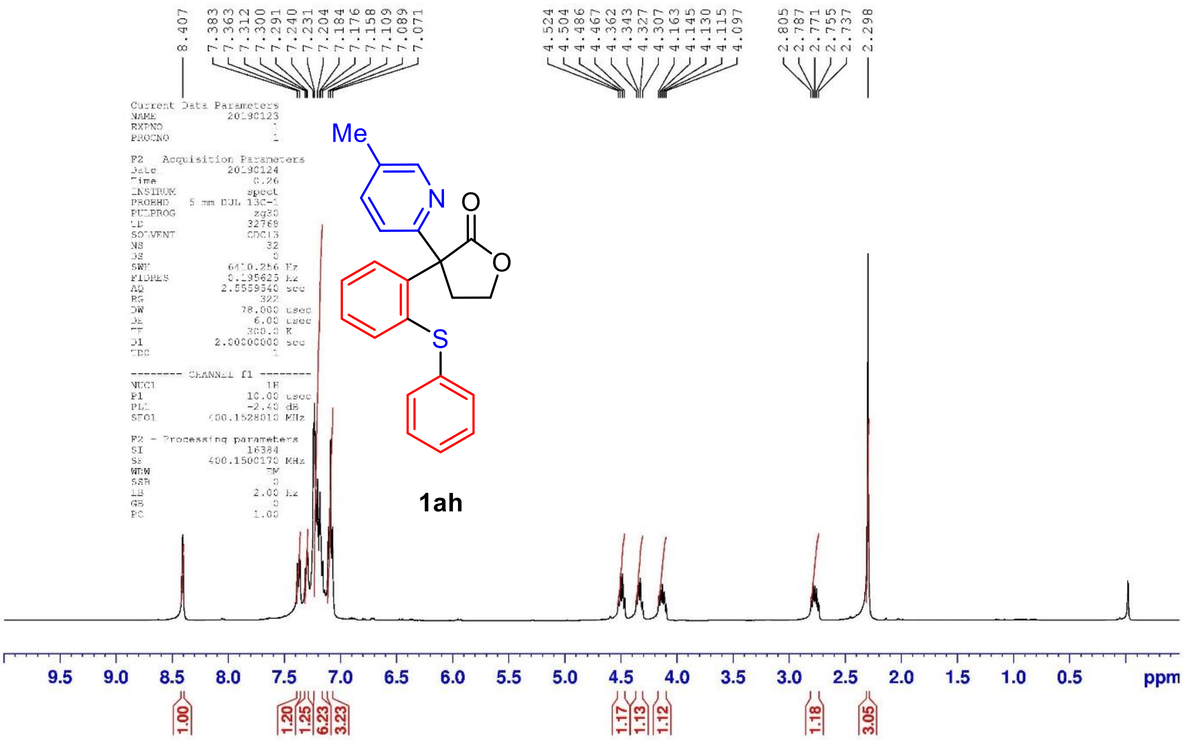

^{1}H NMR (400 MHz, $CDCl_3$) spectrum of compound **1ah**

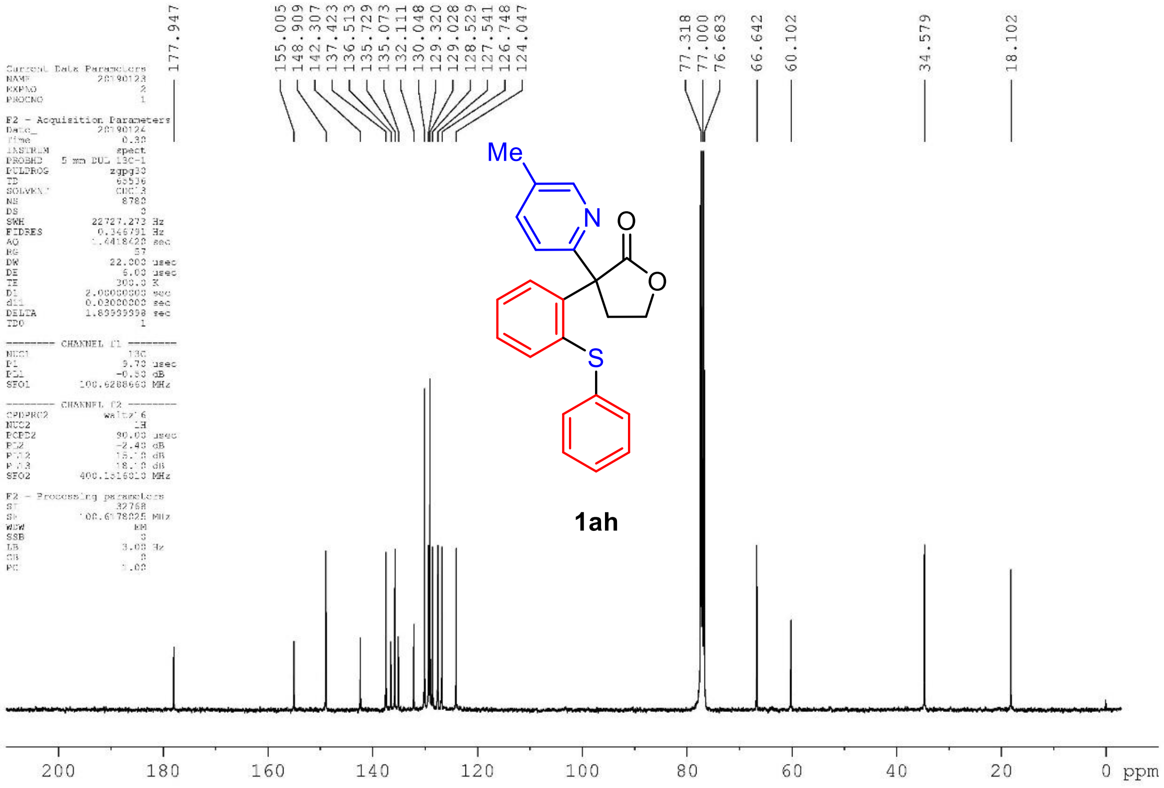

^{13}C{^{1}H} NMR (100 MHz, $CDCl_3$) spectrum of compound **1ah**

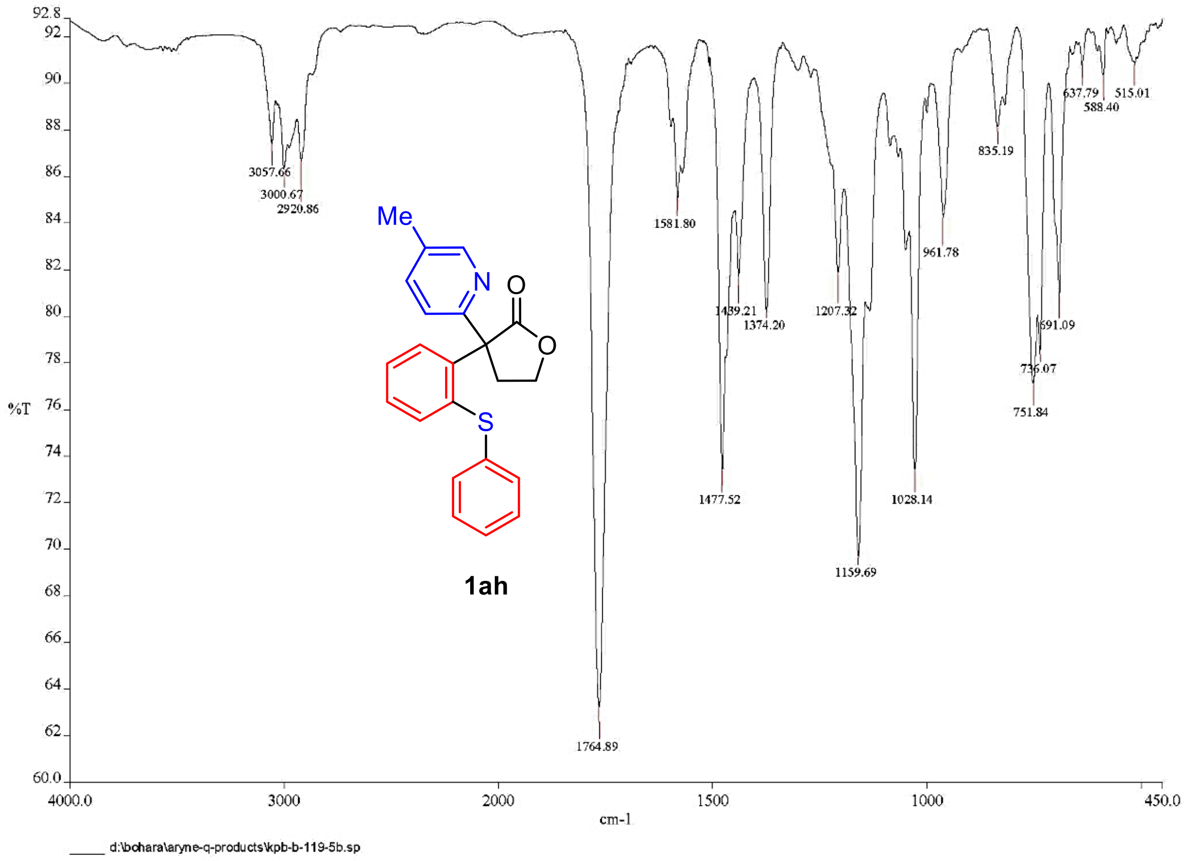

IR spectrum of compound **1ah**

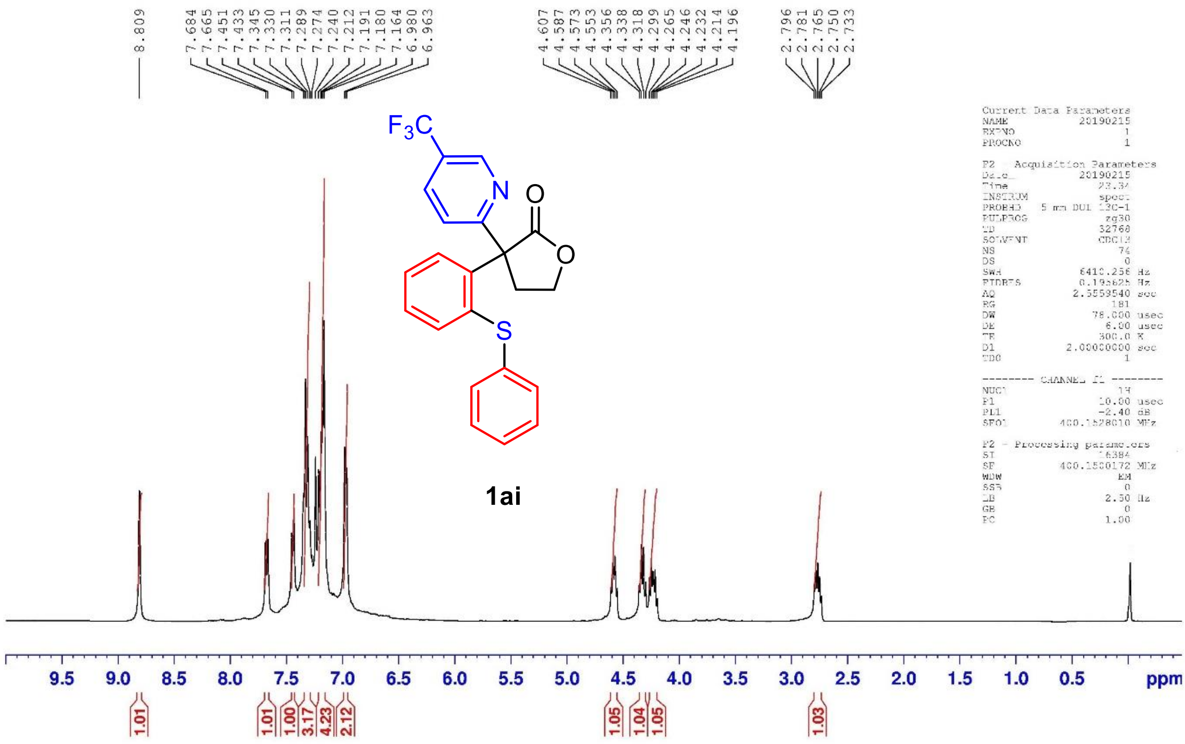

^{1}H NMR (400 MHz, $CDCl_3$) spectrum of compound **1ai**

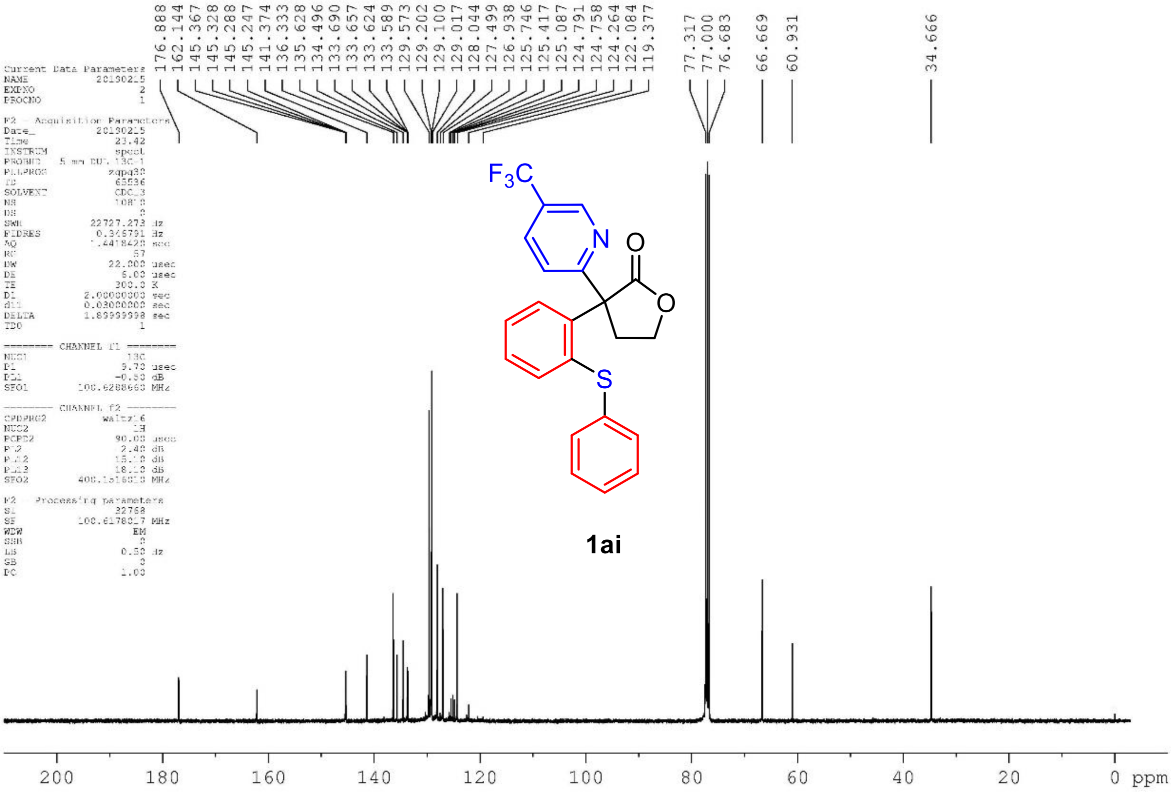

$^{13}C\{^1H\}$ NMR (100 MHz, $CDCl_3$) spectrum of compound **1ai**

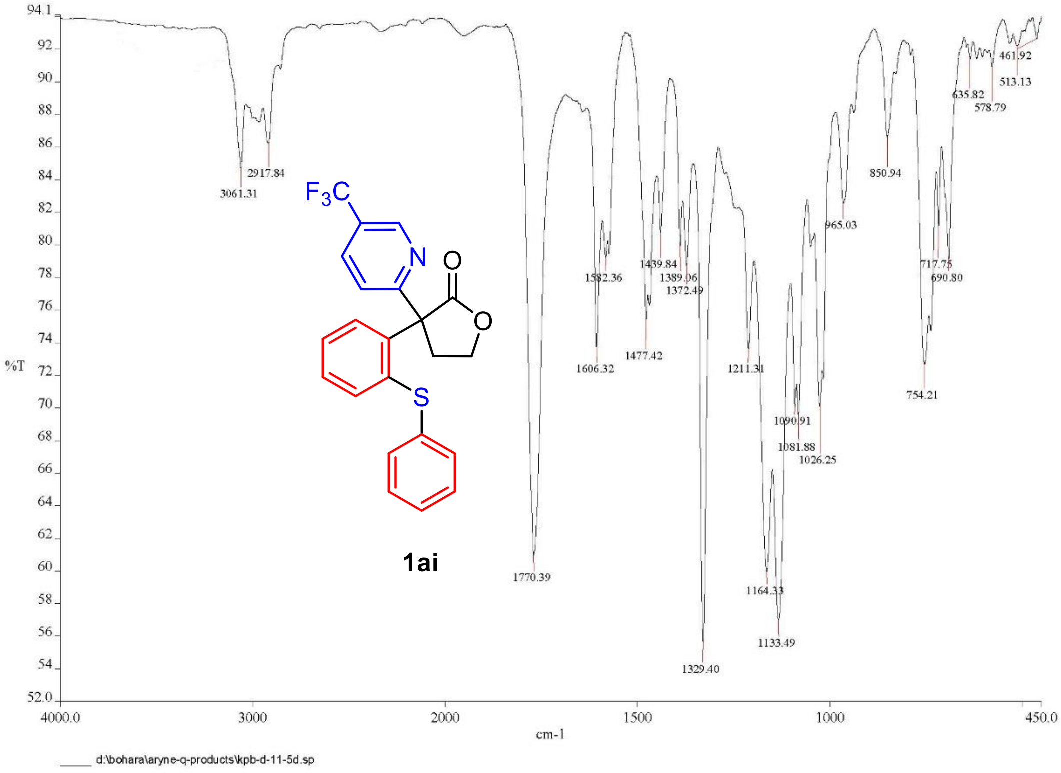

IR spectrum of compound **1ai**

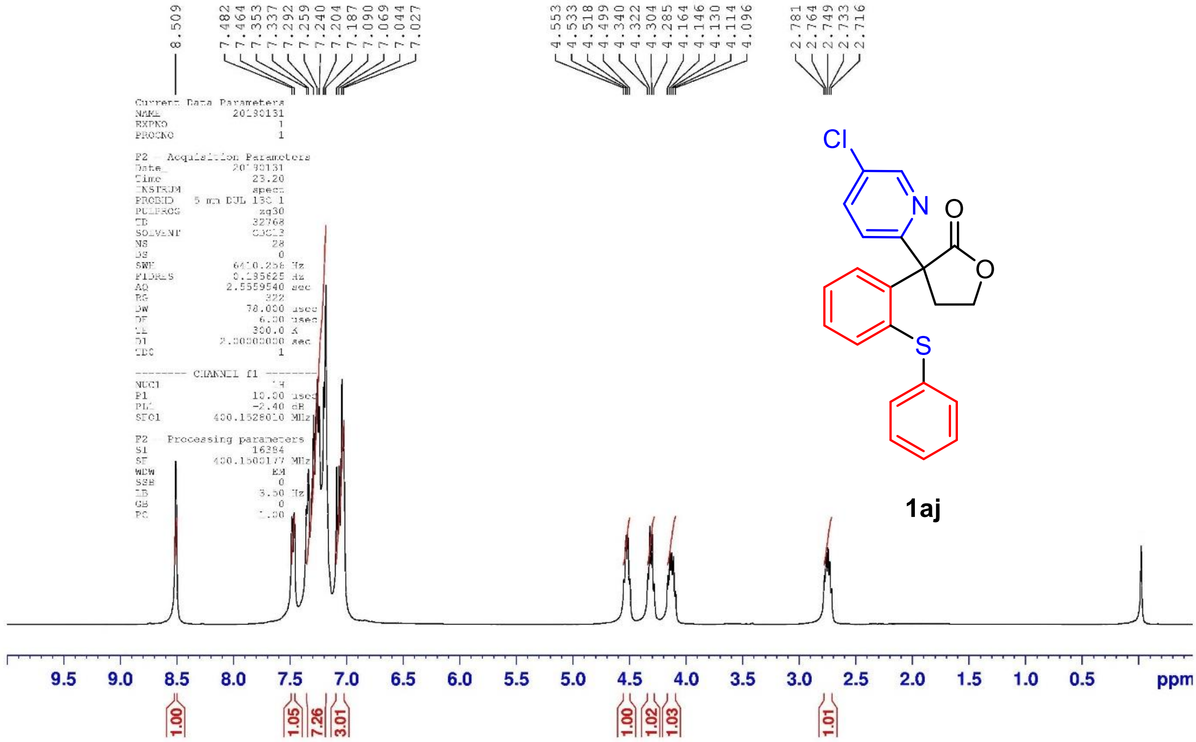

^{1}H NMR (400 MHz, $CDCl_3$) spectrum of compound **1aj**

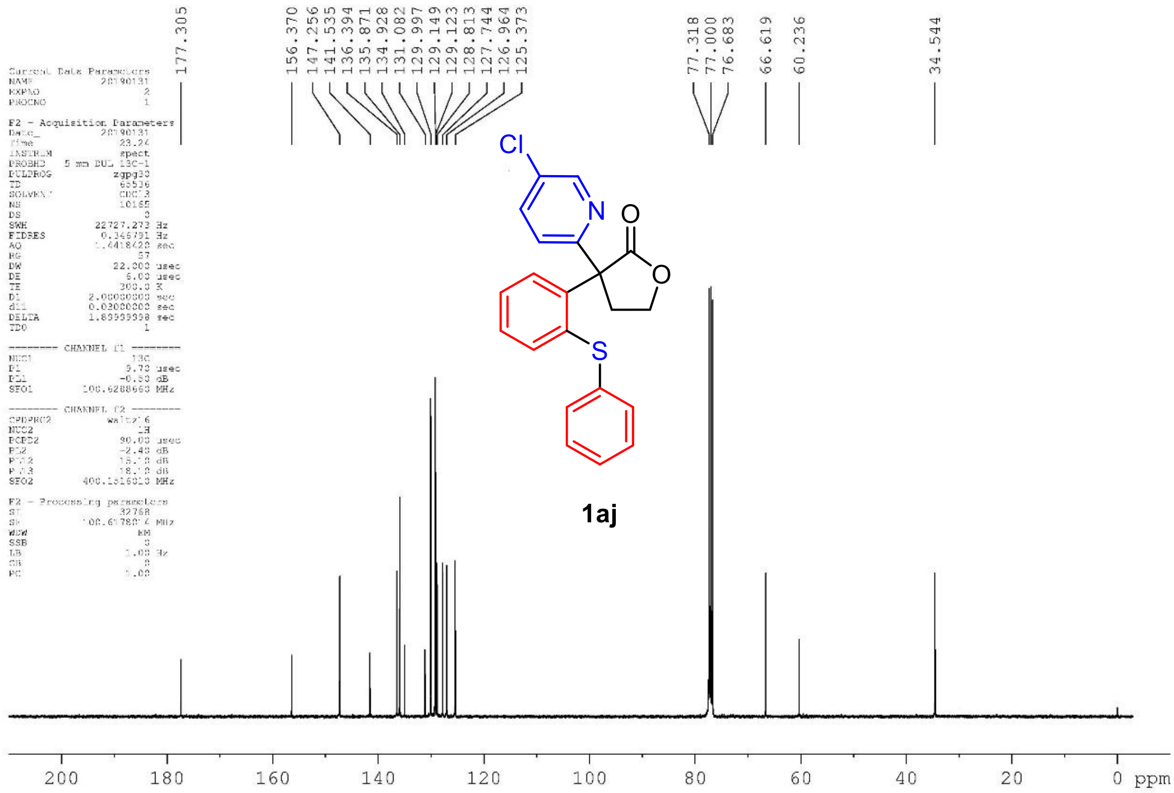

$^{13}C\{^{1}H\}$ NMR (100 MHz, $CDCl_3$) spectrum of compound **1aj**

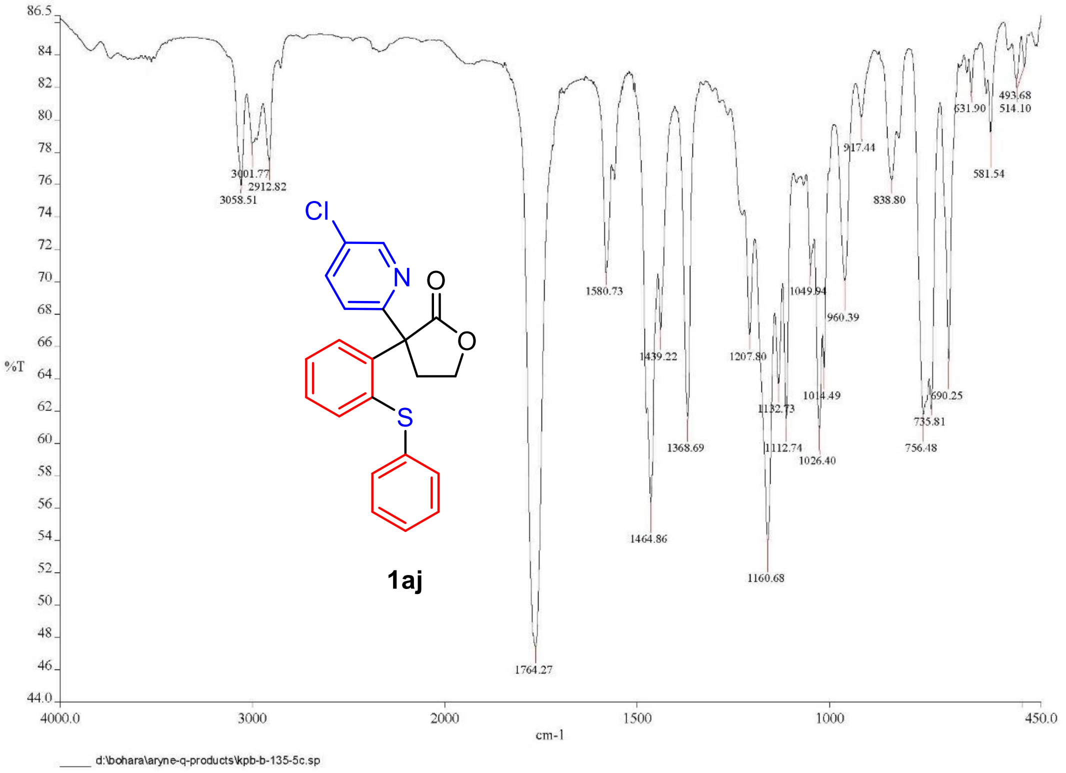

IR spectrum of compound **1aj**

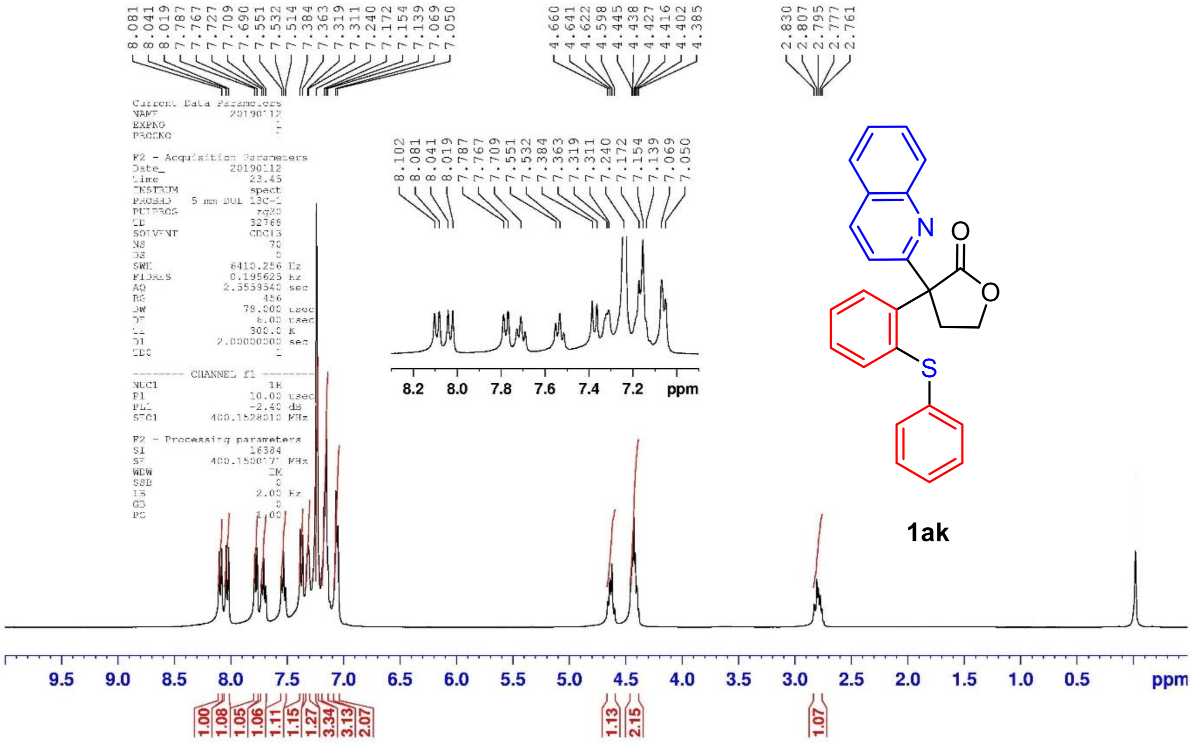

^{1}H NMR (400 MHz, $CDCl_3$) spectrum of compound **1ak**

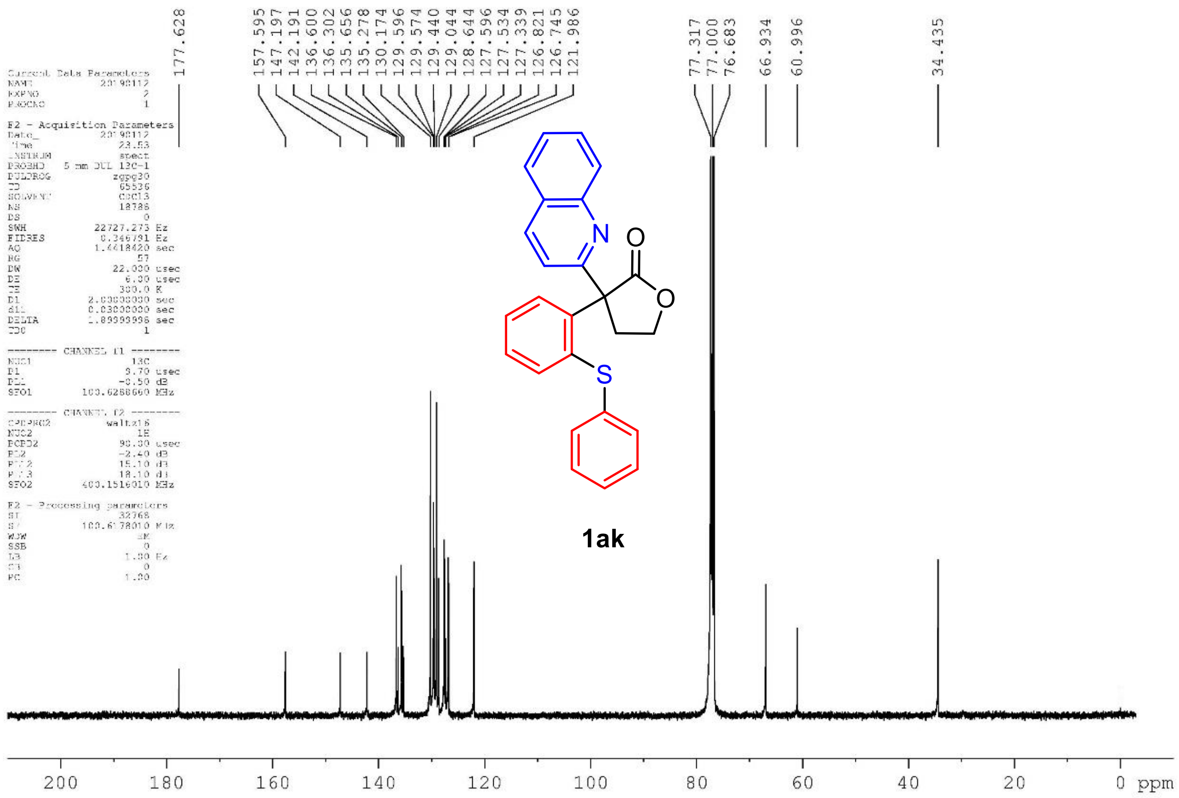

$^{13}C\{^{1}H\}$ NMR (100 MHz, $CDCl_3$) spectrum of compound **1ak**

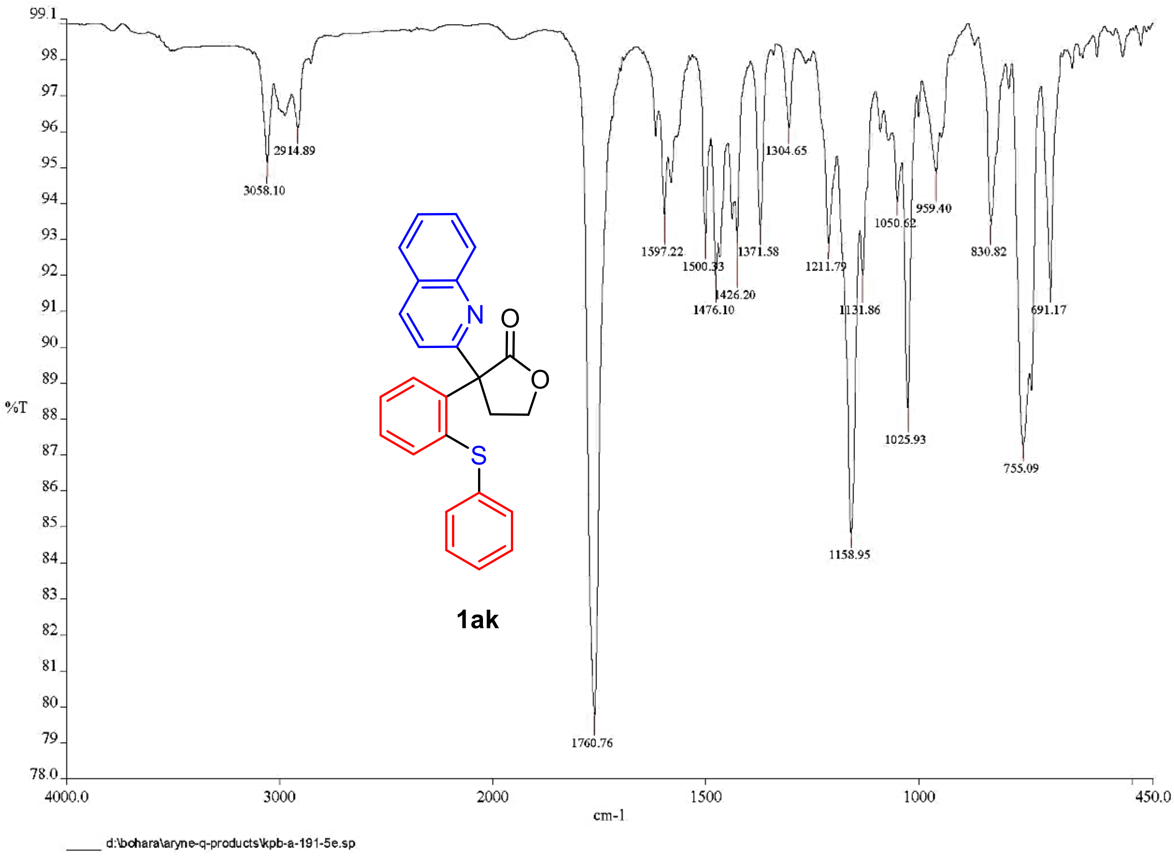

IR spectrum of compound **1ak**

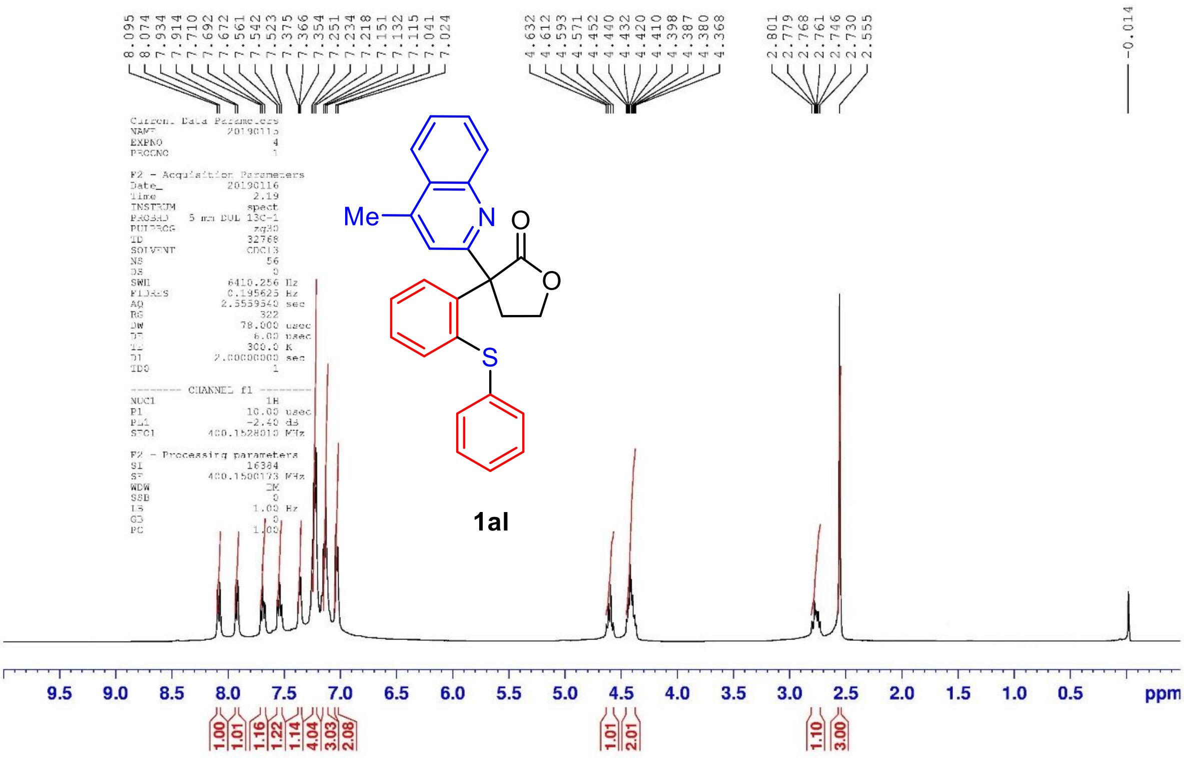

^{1}H NMR (400 MHz, $CDCl_3$) spectrum of compound **1al**

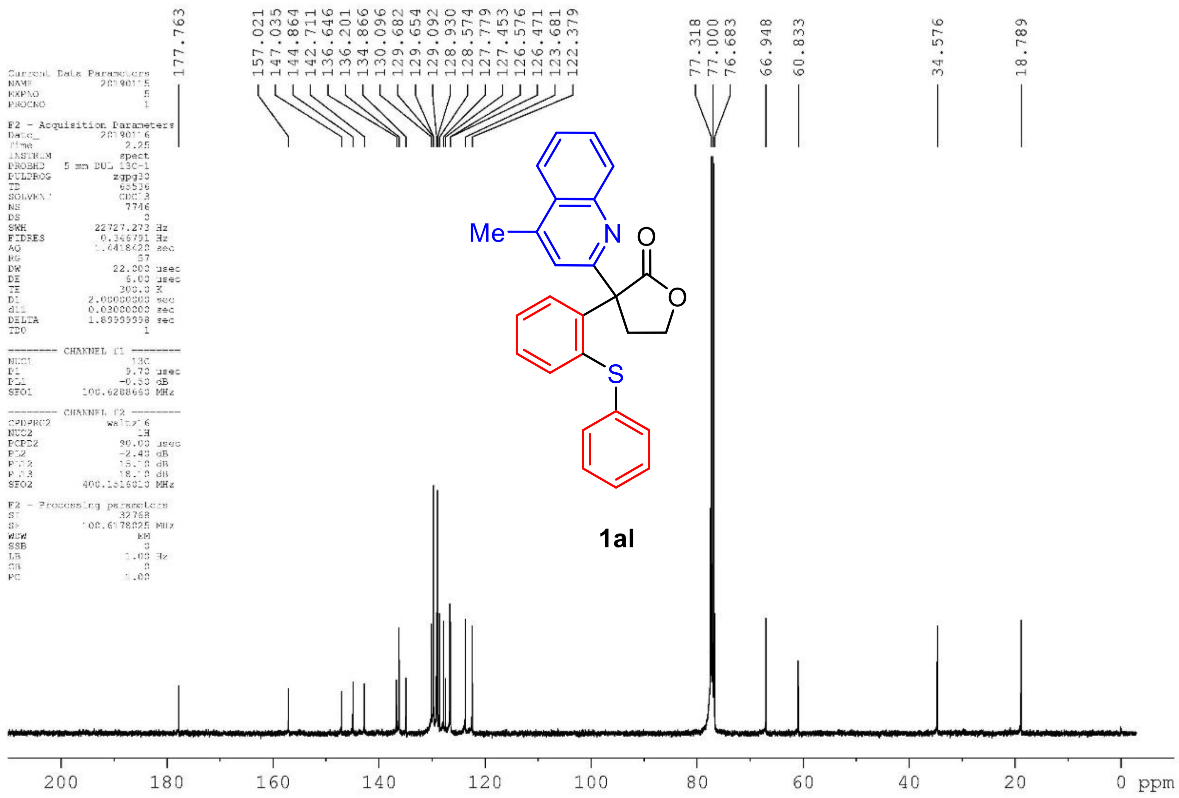

$^{13}C\{^1H\}$ NMR (100 MHz, $CDCl_3$) spectrum of compound **1al**

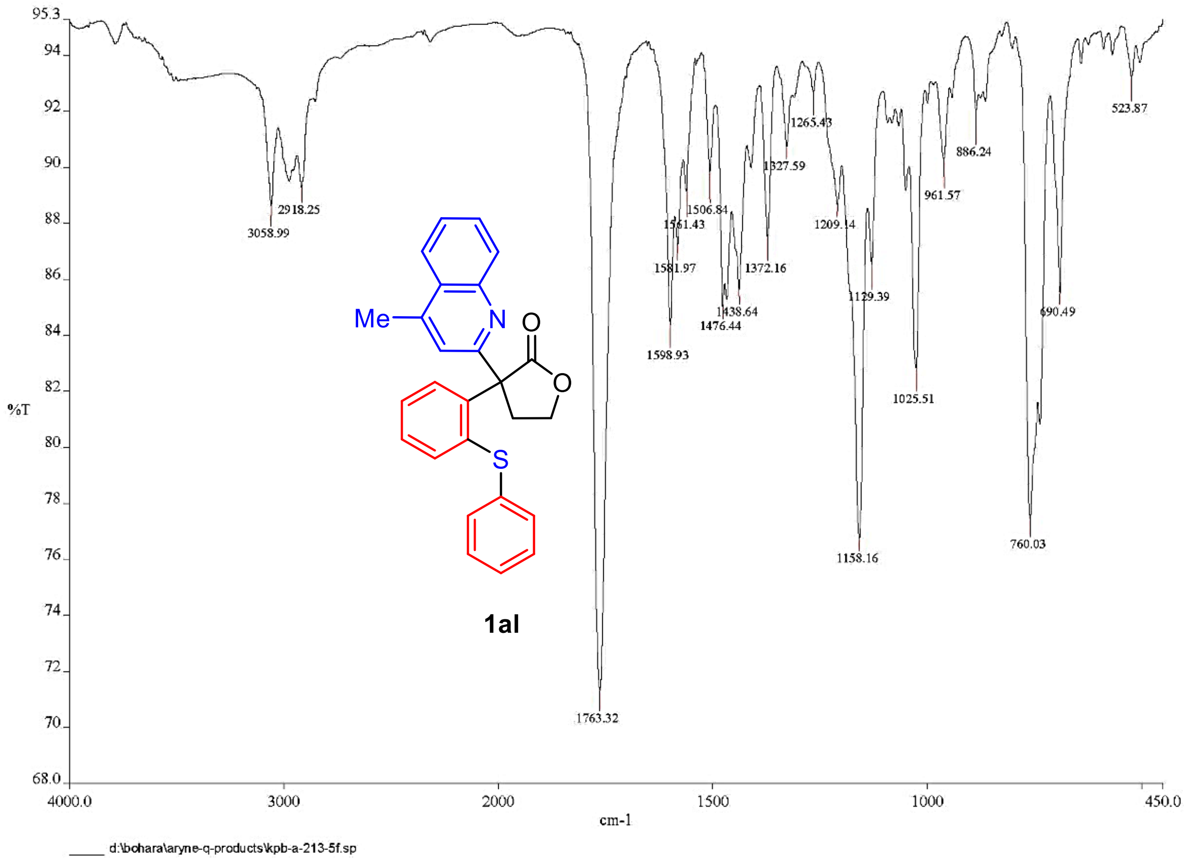

IR spectrum of compound **1al**

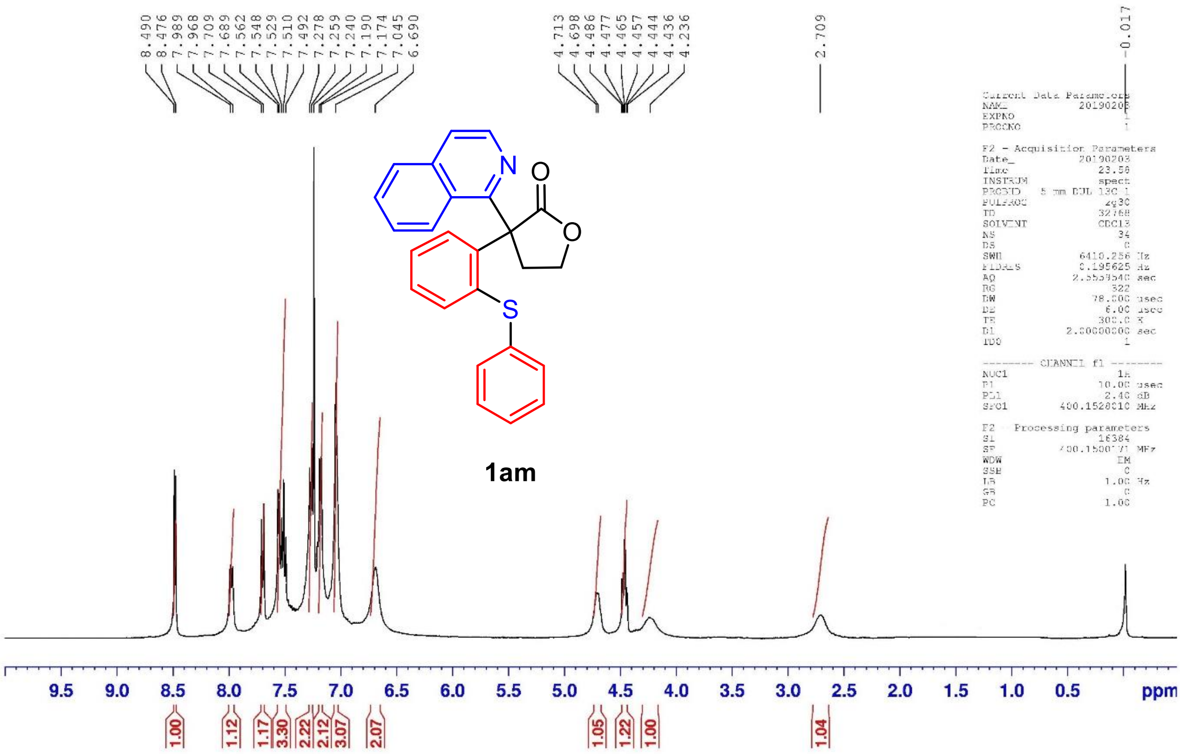

^{1}H NMR (400 MHz, $CDCl_3$) spectrum of compound **1am**

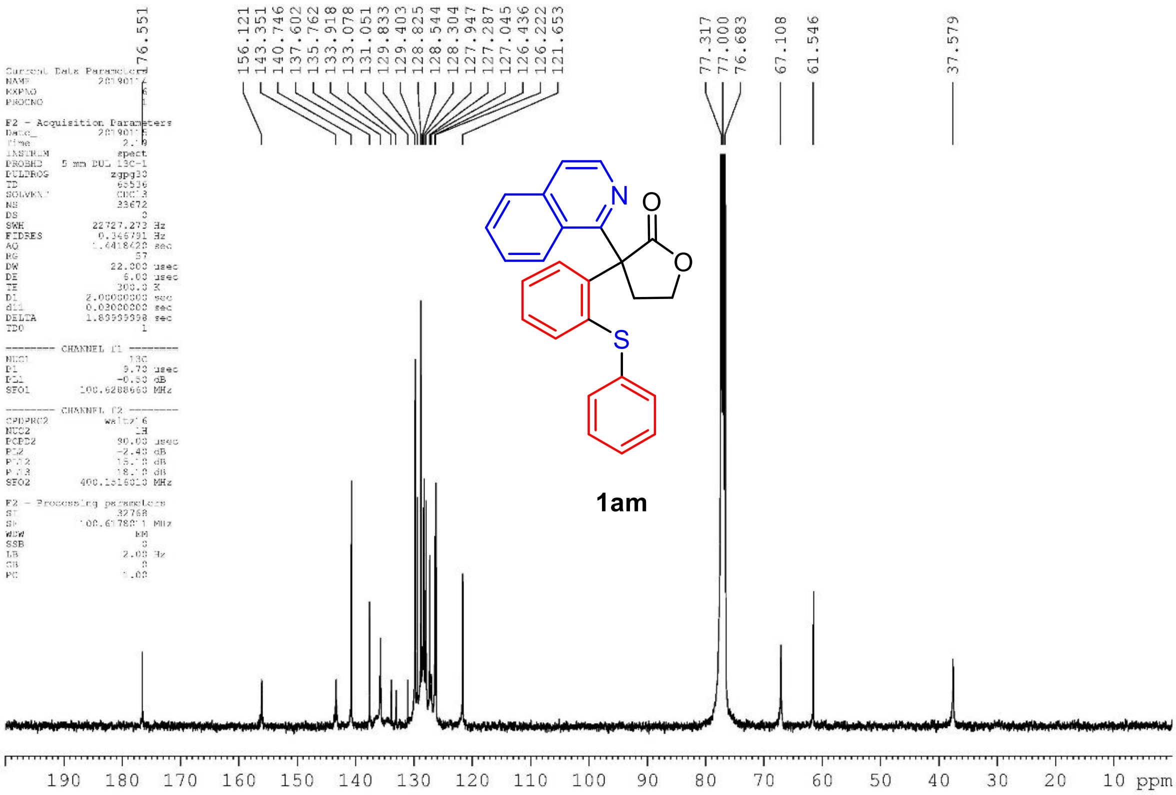

$^{13}C\{^{1}H\}$ NMR (100 MHz, $CDCl_3$) spectrum of compound **1am**

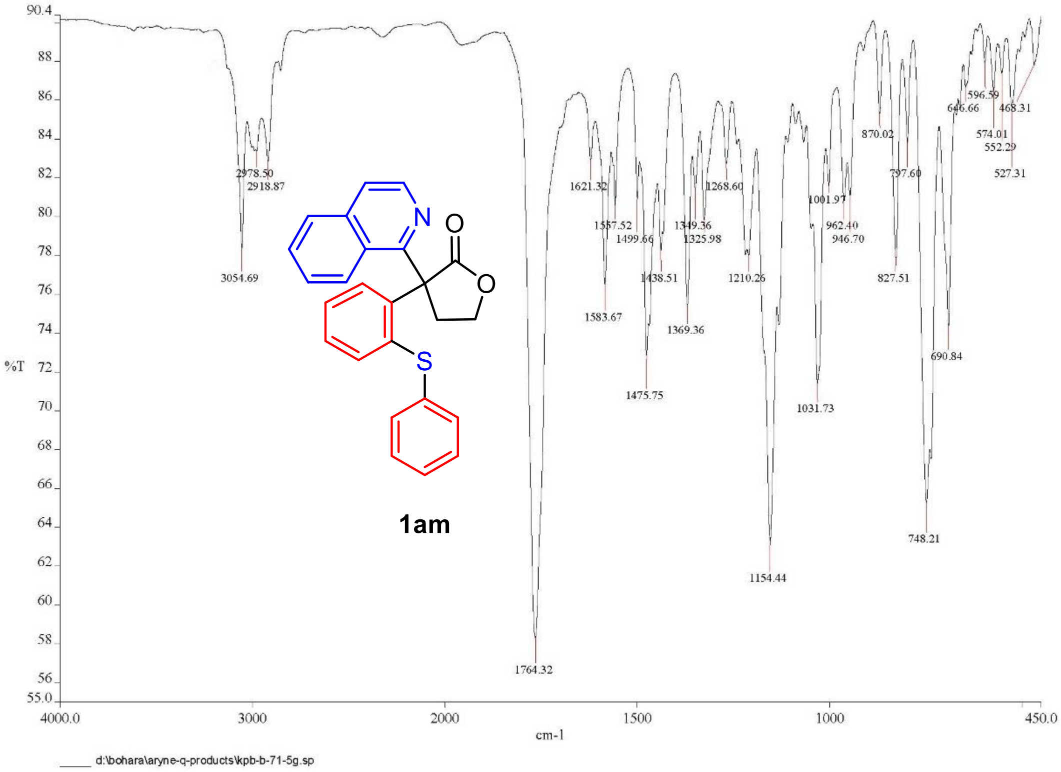

IR spectrum of compound **1am**

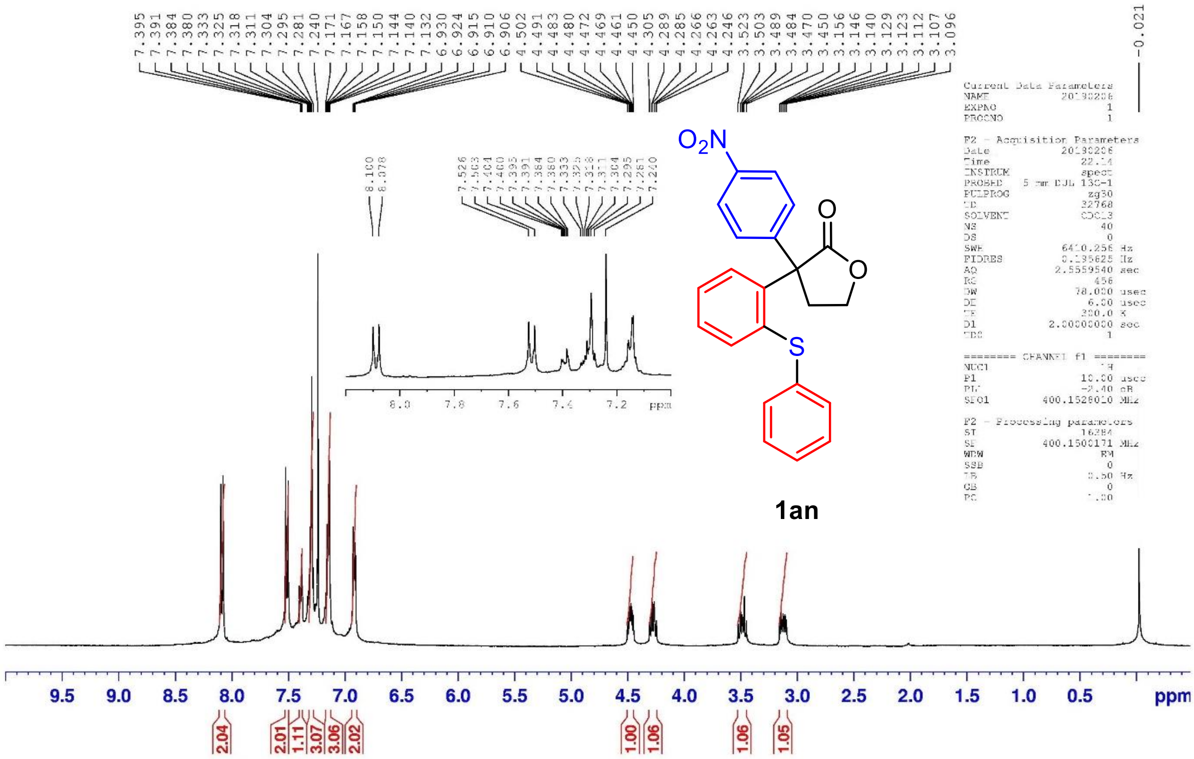

^{1}H NMR (400 MHz, $CDCl_3$) spectrum of compound **1an**

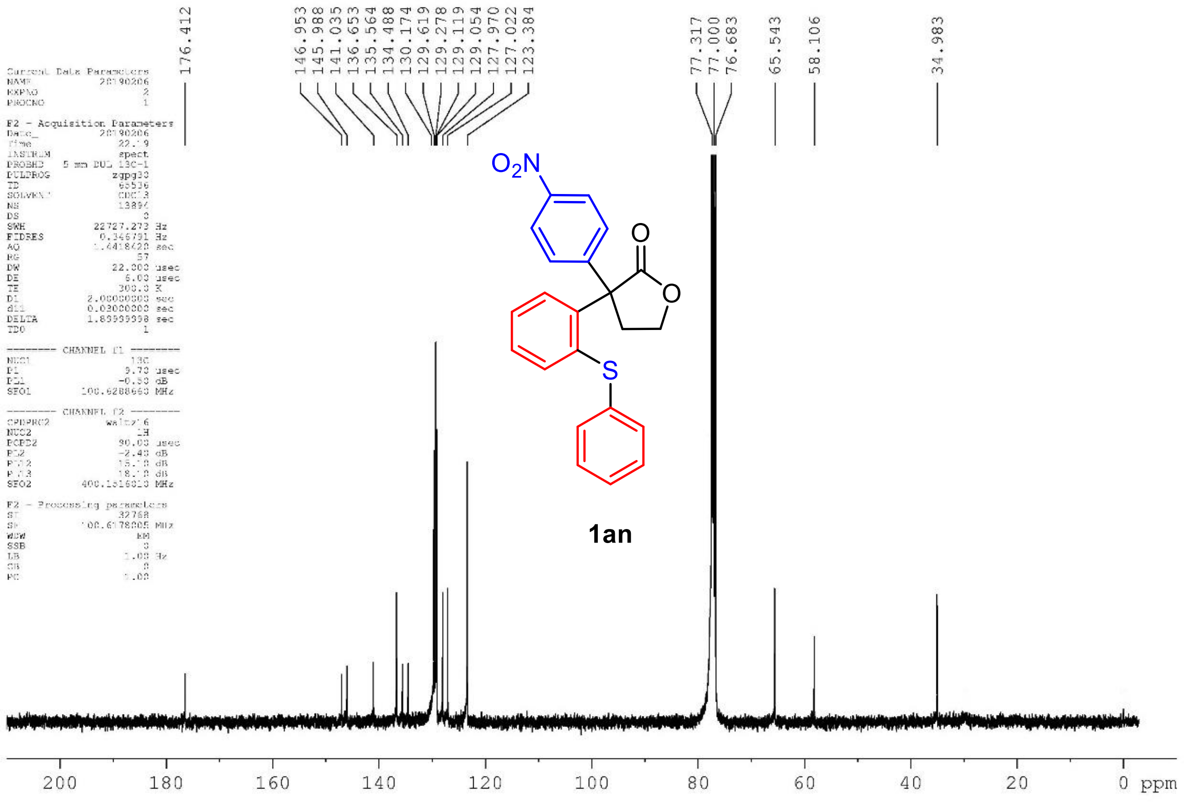

$^{13}C\{^{1}H\}$ NMR (100 MHz, $CDCl_3$) spectrum of compound **1an**

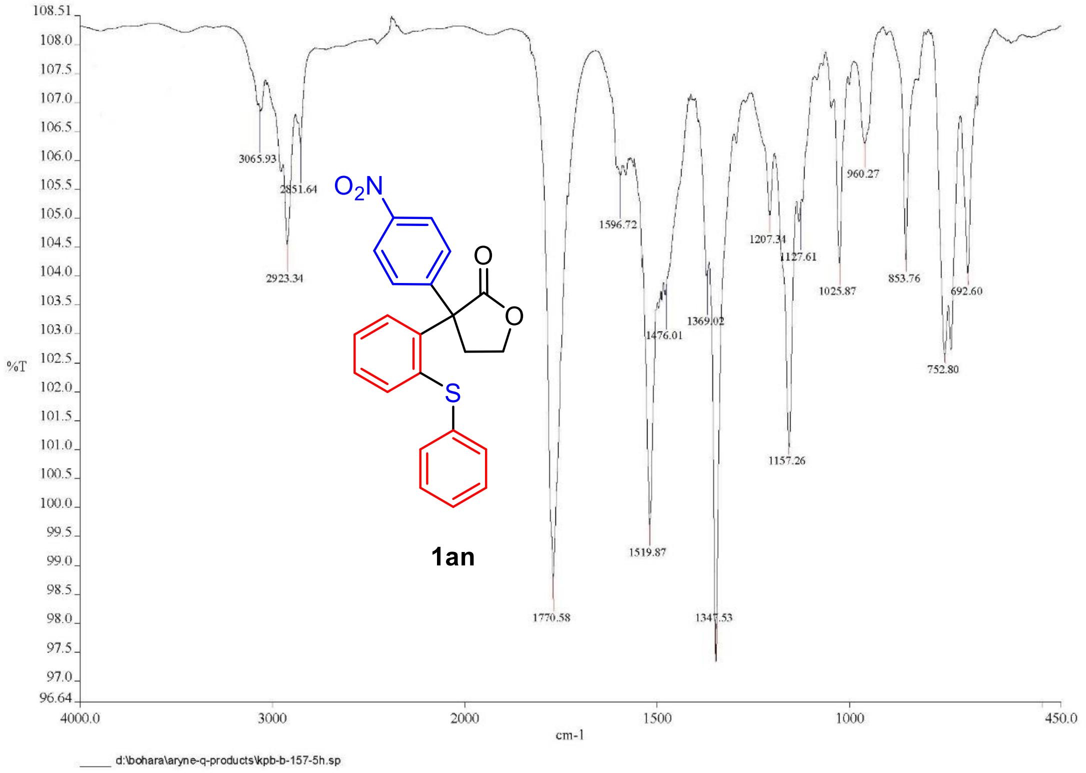

IR spectrum of compound **1an**

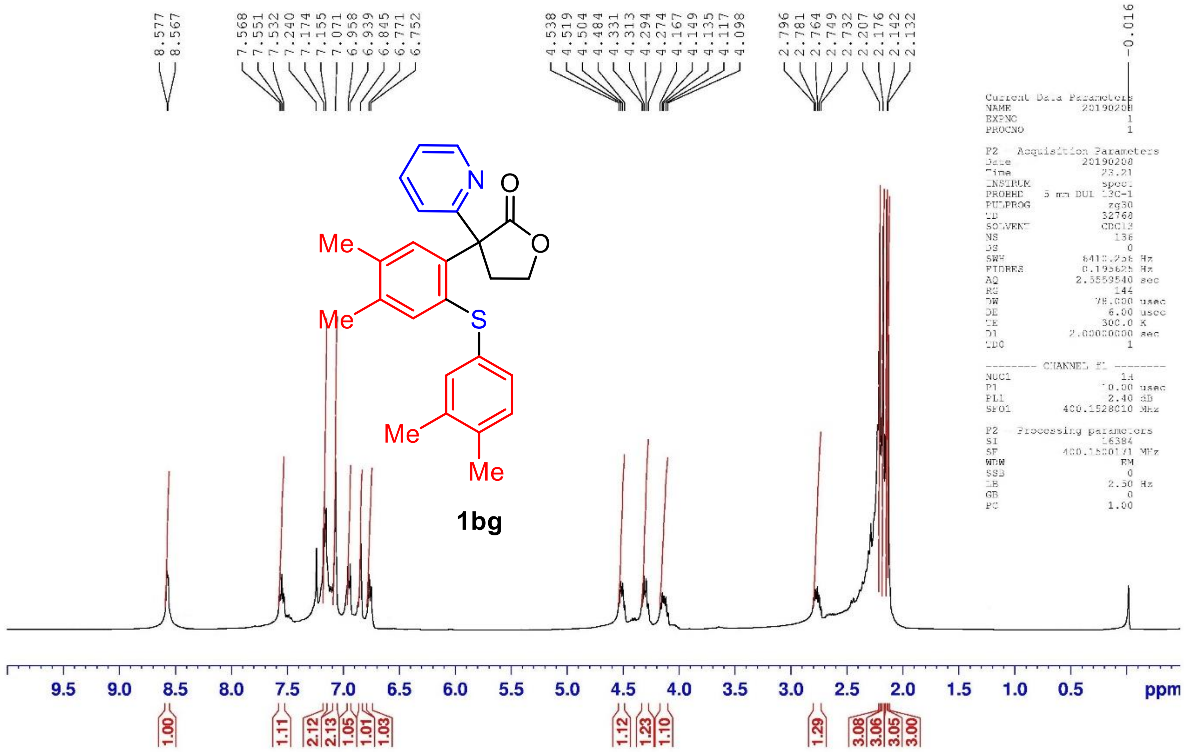

^{1}H NMR (400 MHz, CDCl3) spectrum of compound **1bg**

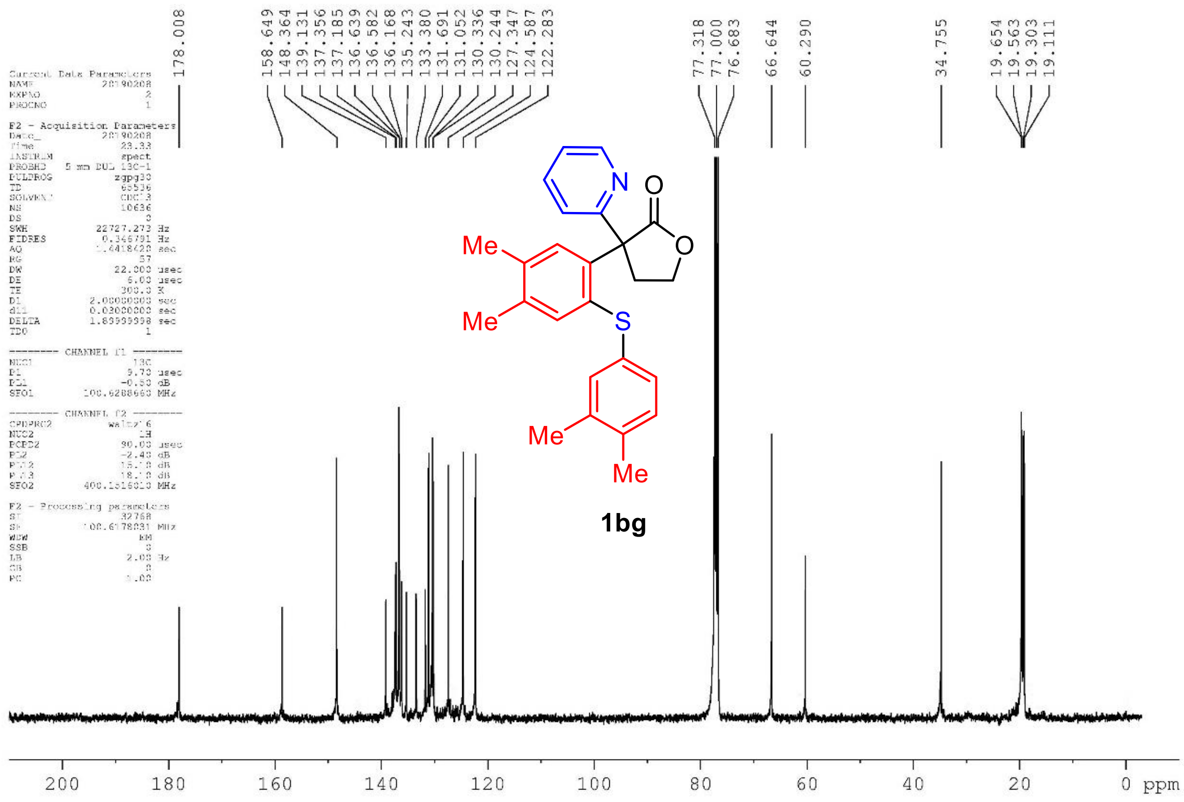

$^{13}C\{^{1}H\}$ NMR (100 MHz, $CDCl_3$) spectrum of compound **1bg**

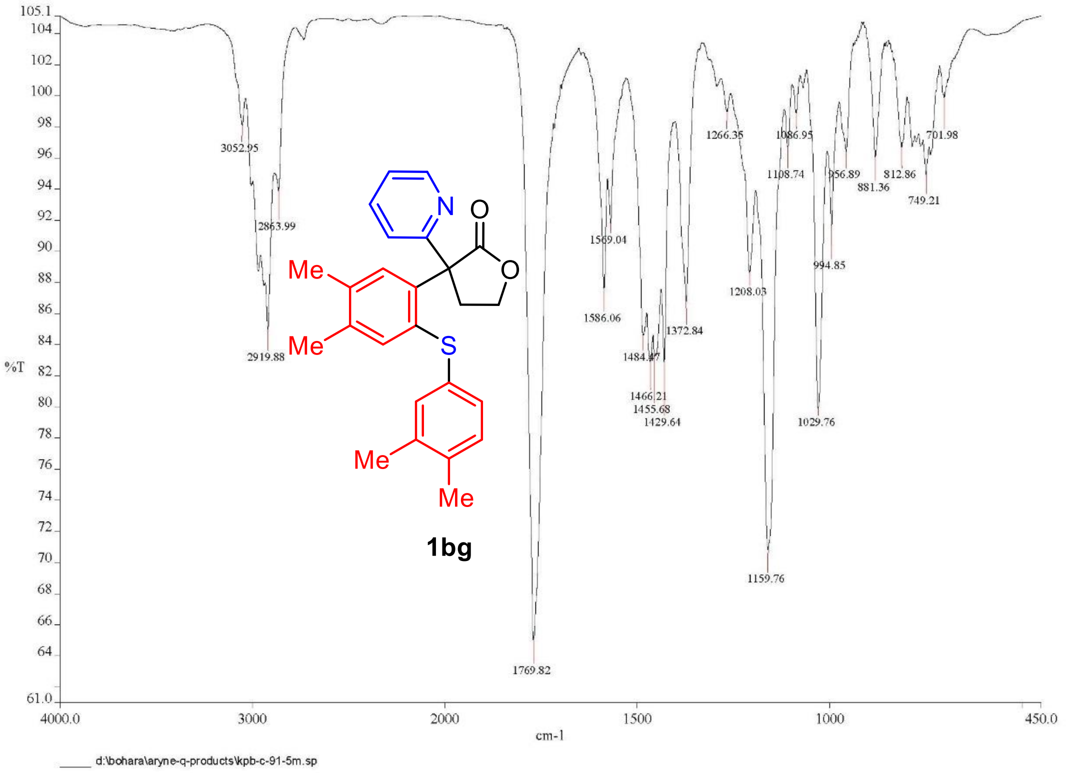

IR spectrum of compound **1bg**

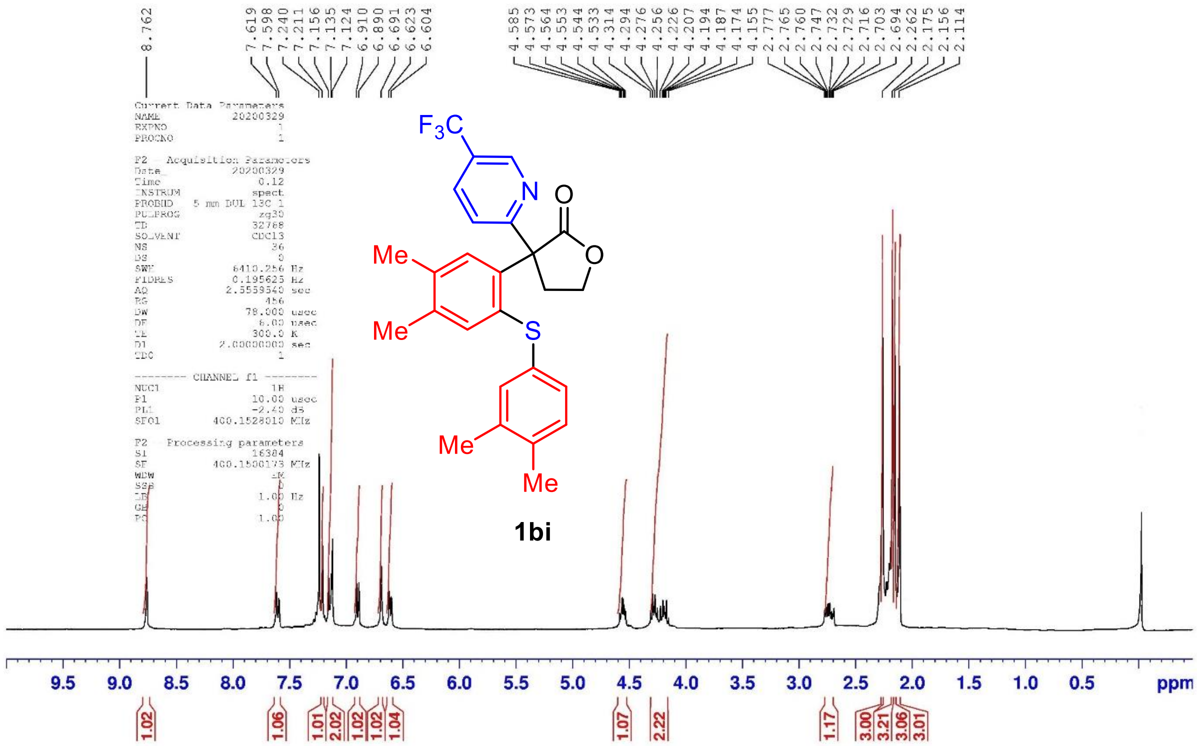

^{1}H NMR (400 MHz, $CDCl_3$) spectrum of compound **1bi**

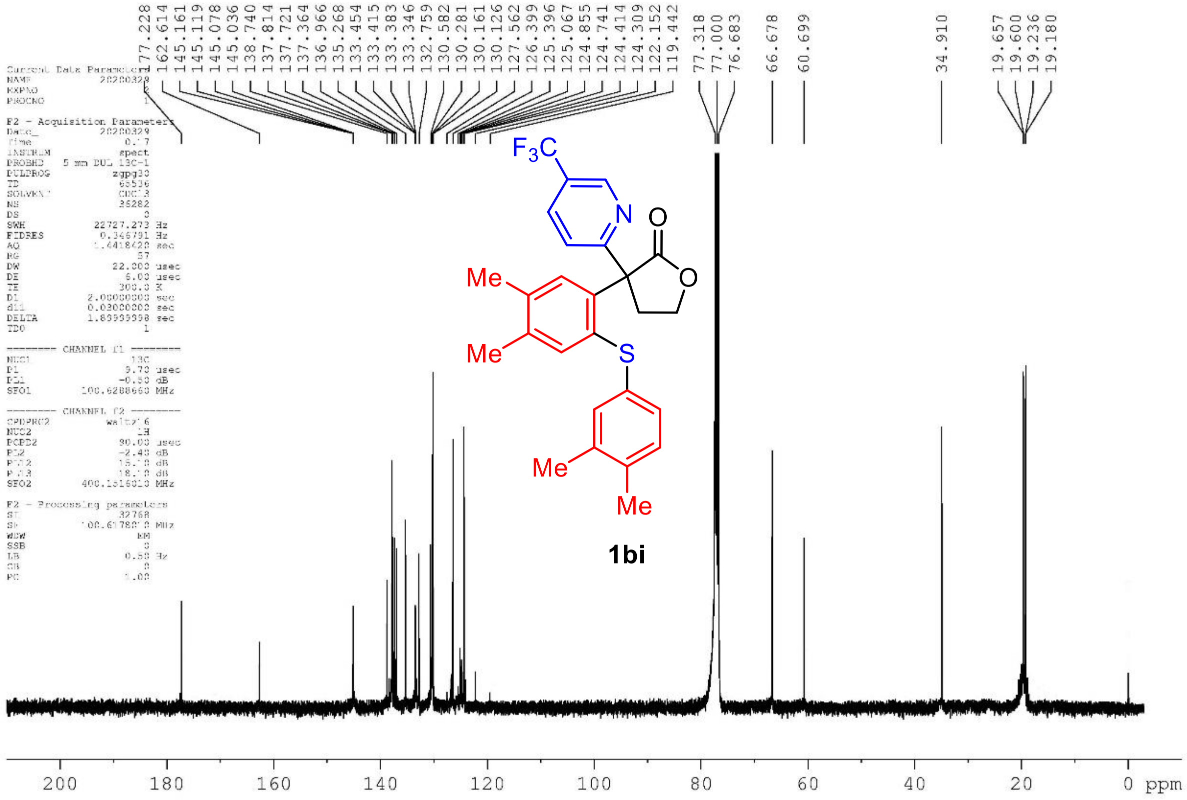

^{13}C{^{1}H} NMR (100 MHz, $CDCl_3$) spectrum of compound **1bi**

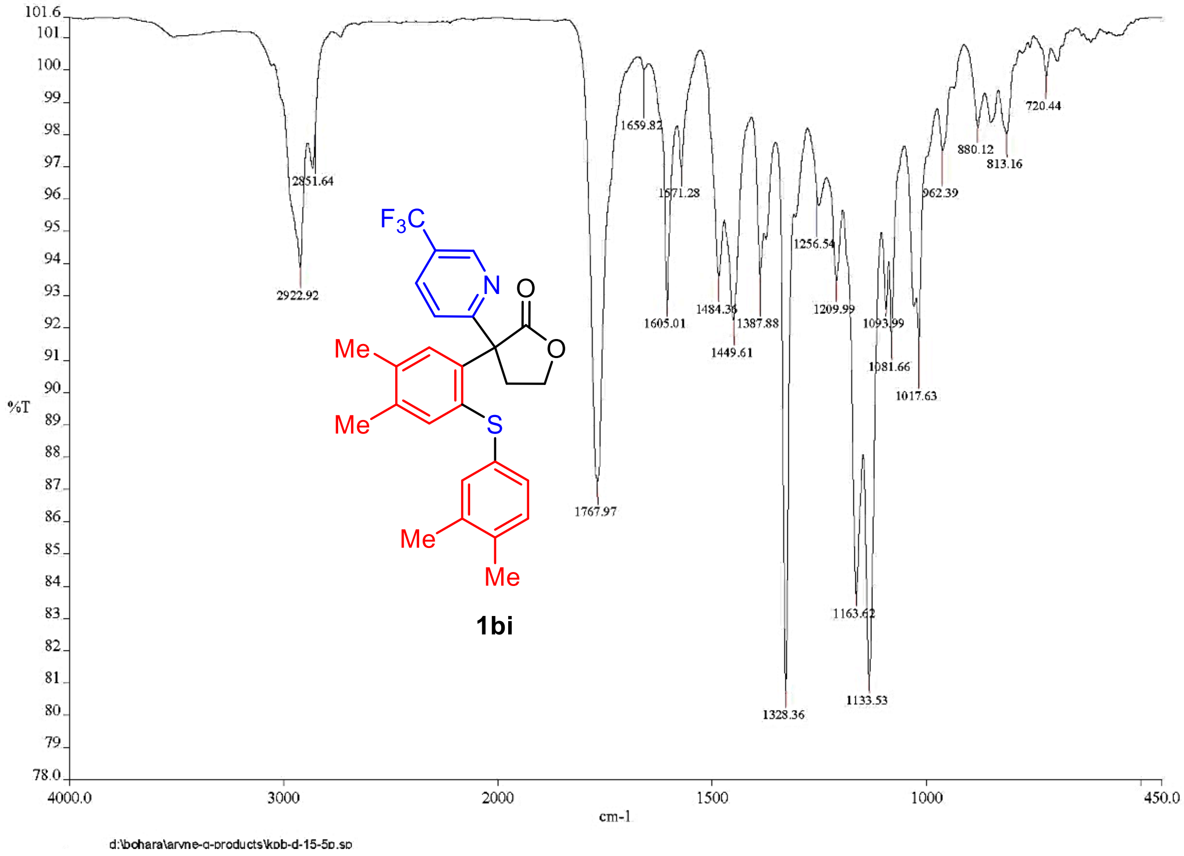

IR spectrum of compound **1bi**

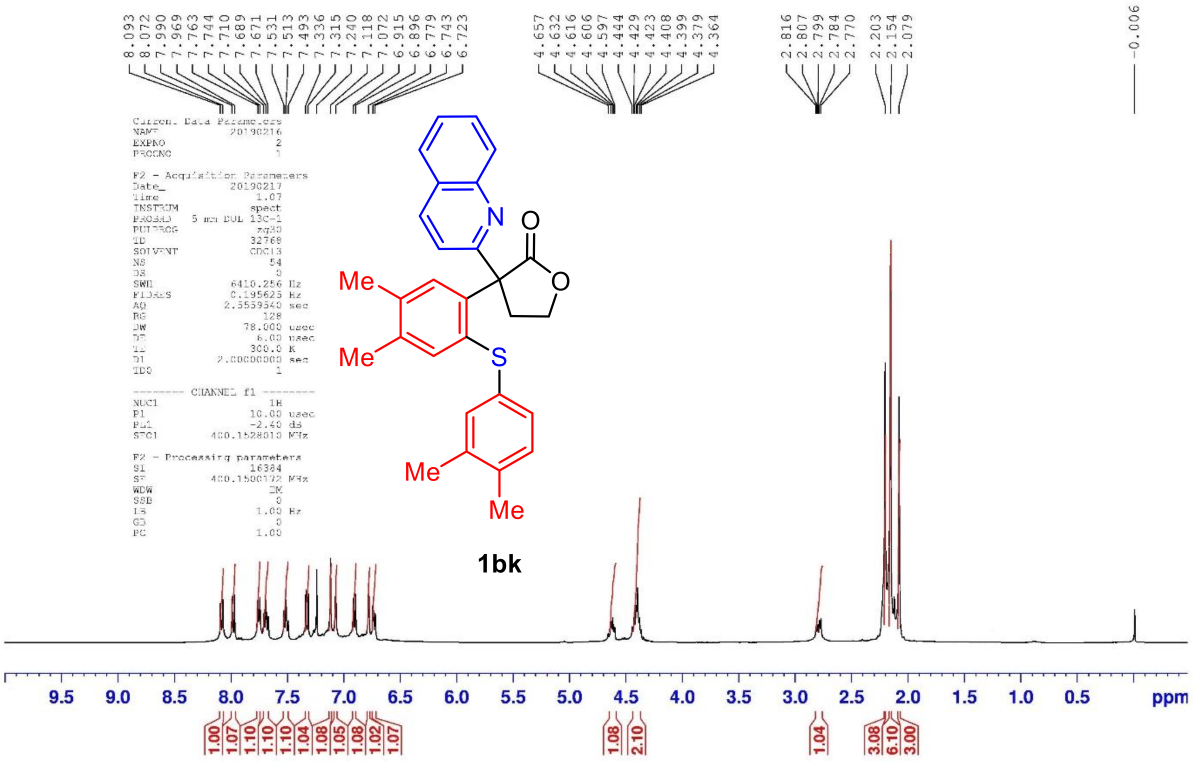

^{1}H NMR (400 MHz, $CDCl_3$) spectrum of compound **1bk**

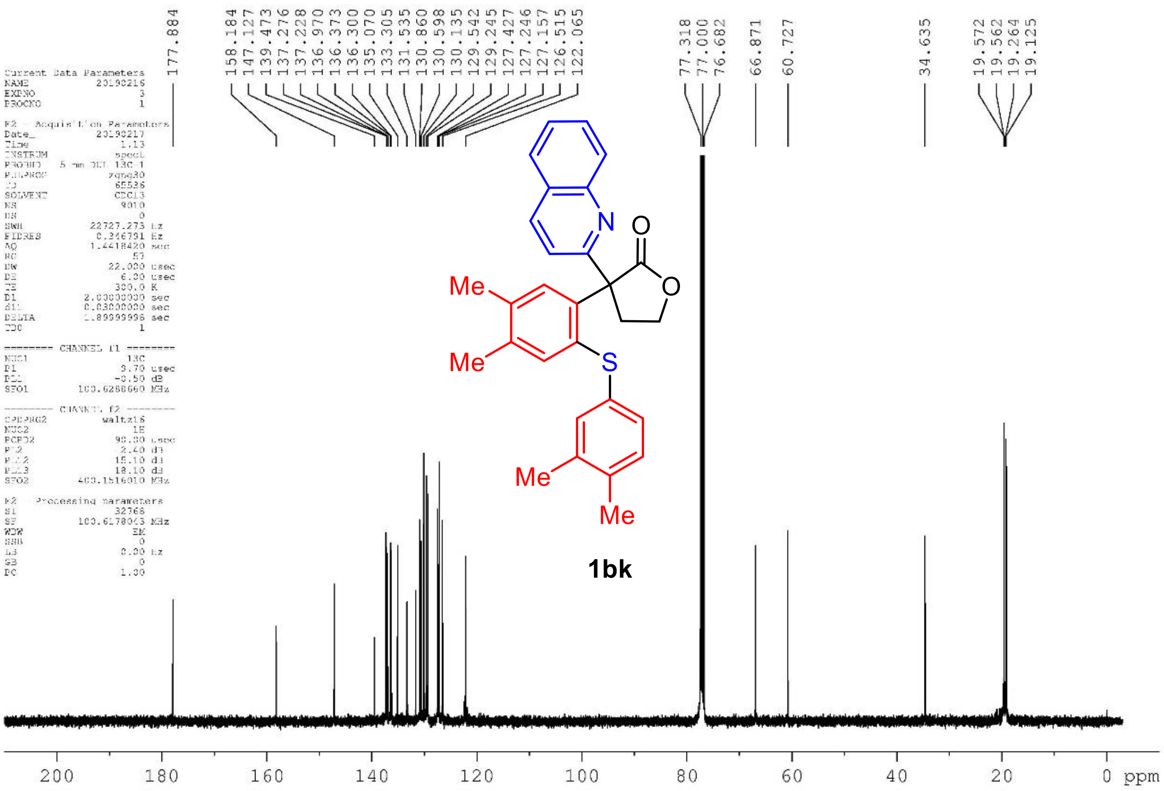

$^{13}C\{^{1}H\}$ NMR (100 MHz, $CDCl_3$) spectrum of compound **1bk**

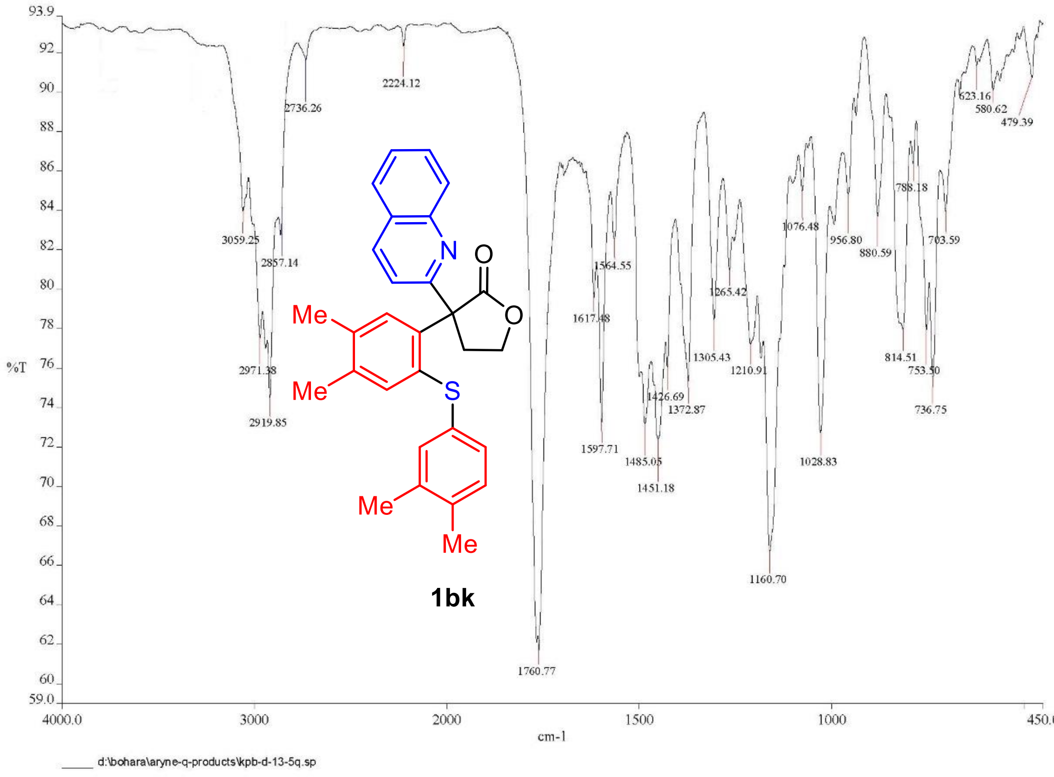

IR spectrum of compound **1bk**

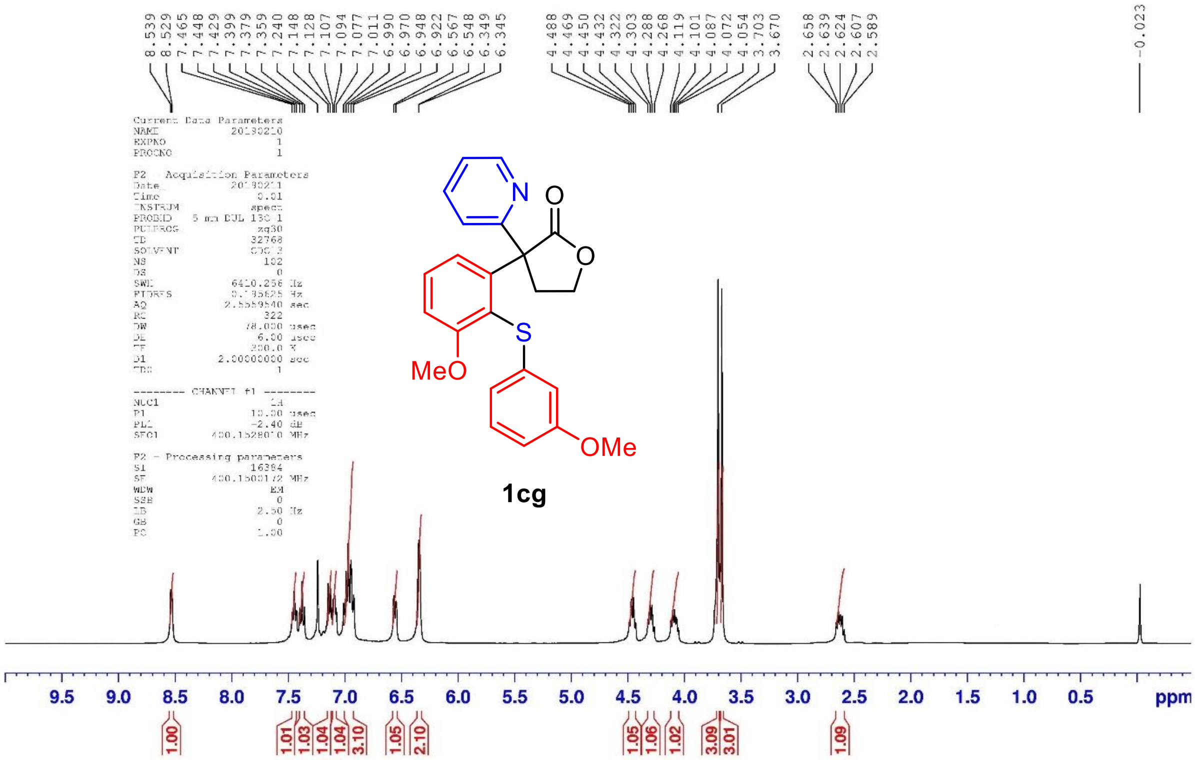

^{1}H NMR (400 MHz, $CDCl_3$) spectrum of compound **1cg**

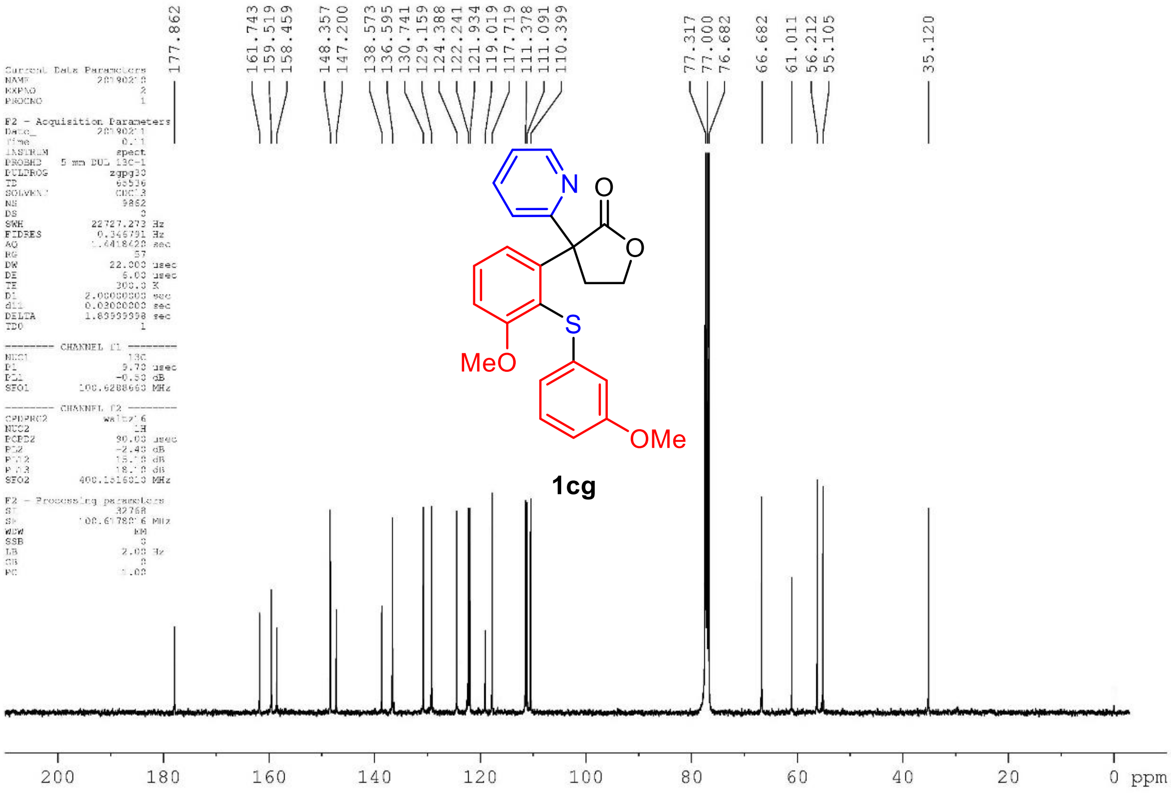

^{13}C{^{1}H} NMR (100 MHz, $CDCl_3$) spectrum of compound **1cg**

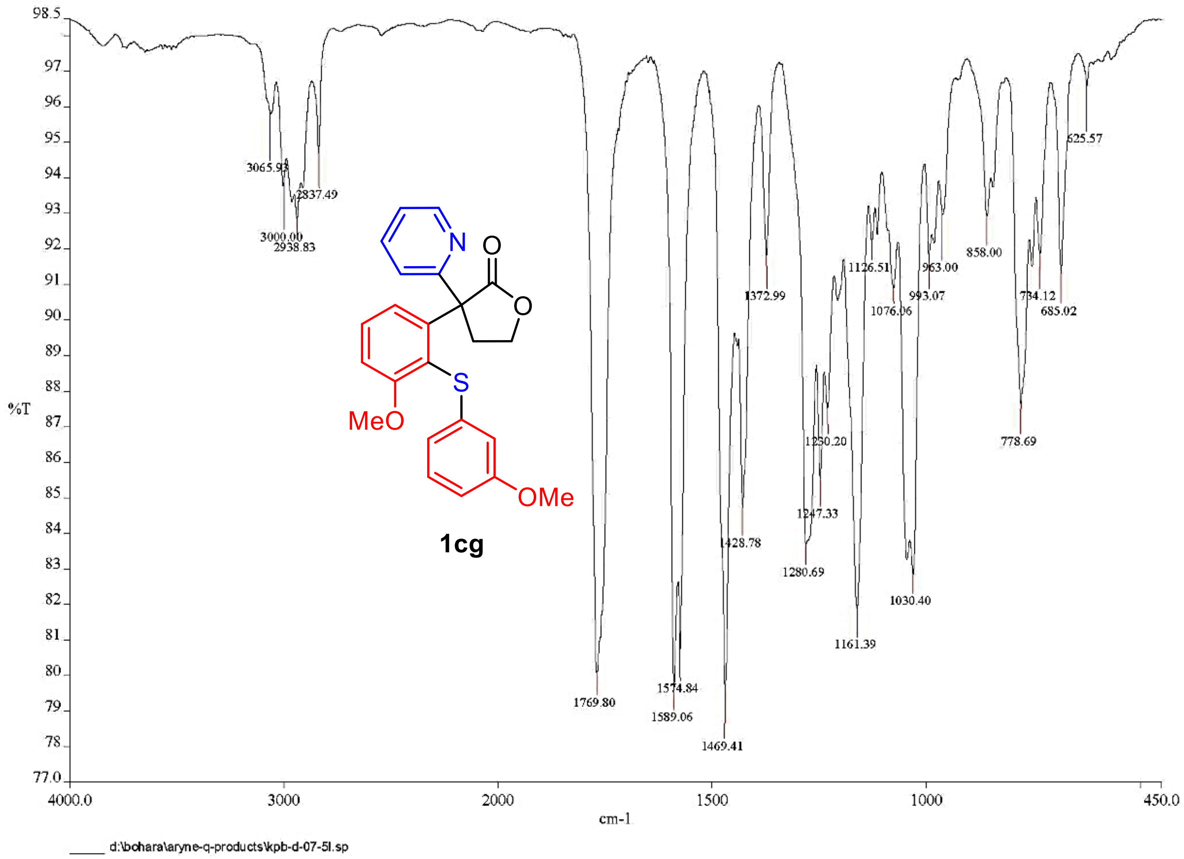

IR spectrum of compound **1cg**

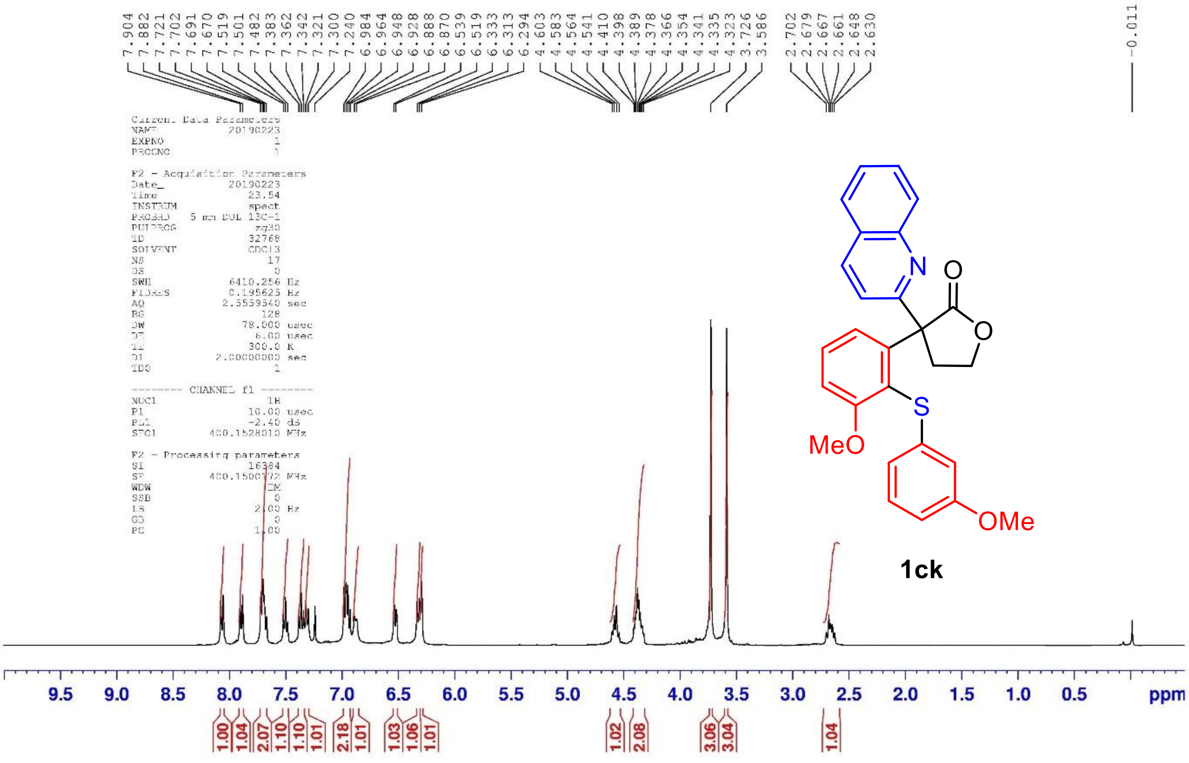

^{1}H NMR (400 MHz, $CDCl_3$) spectrum of compound **1ck**

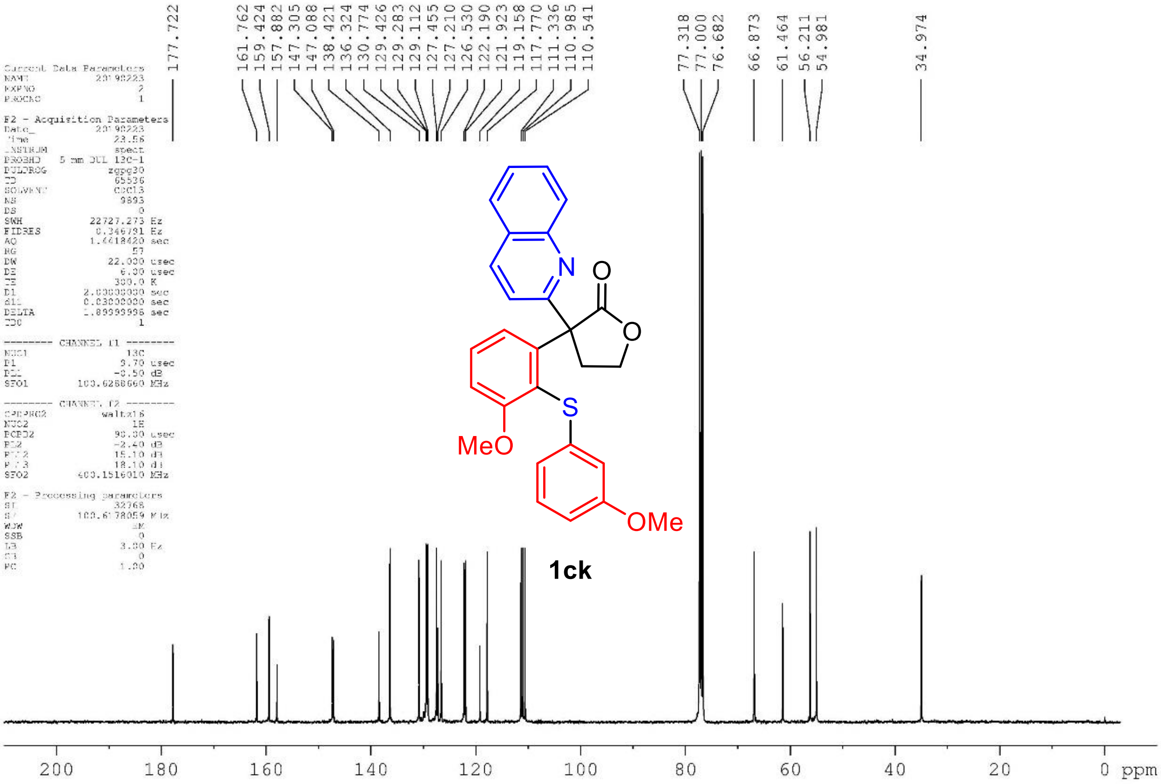

$^{13}C\{^1H\}$ NMR (100 MHz, $CDCl_3$) spectrum of compound **1ck**

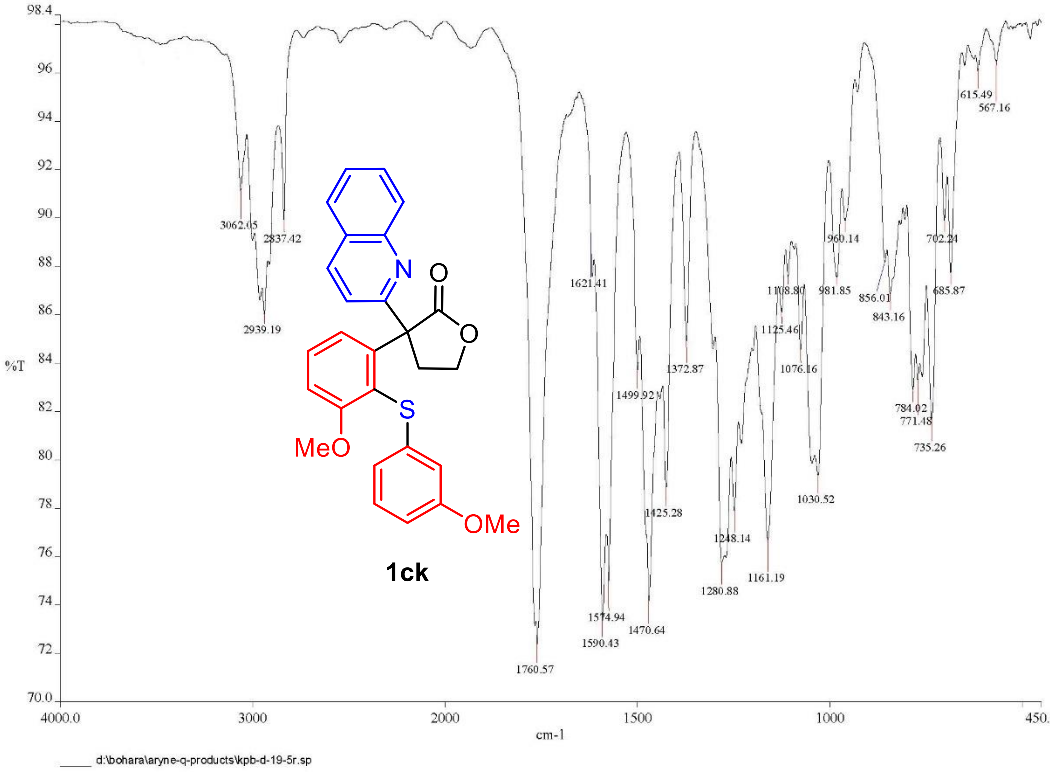

IR spectrum of compound **1ck**

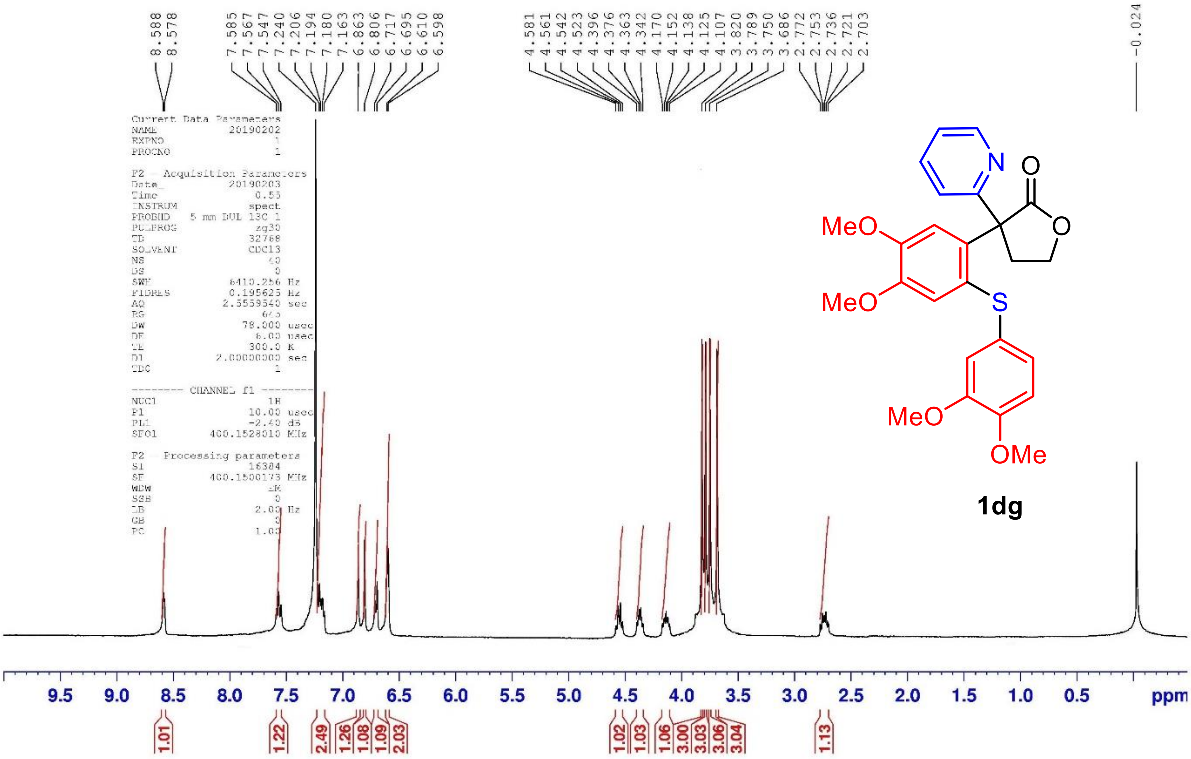

^{1}H NMR (400 MHz, $CDCl_3$) spectrum of compound **1dg**

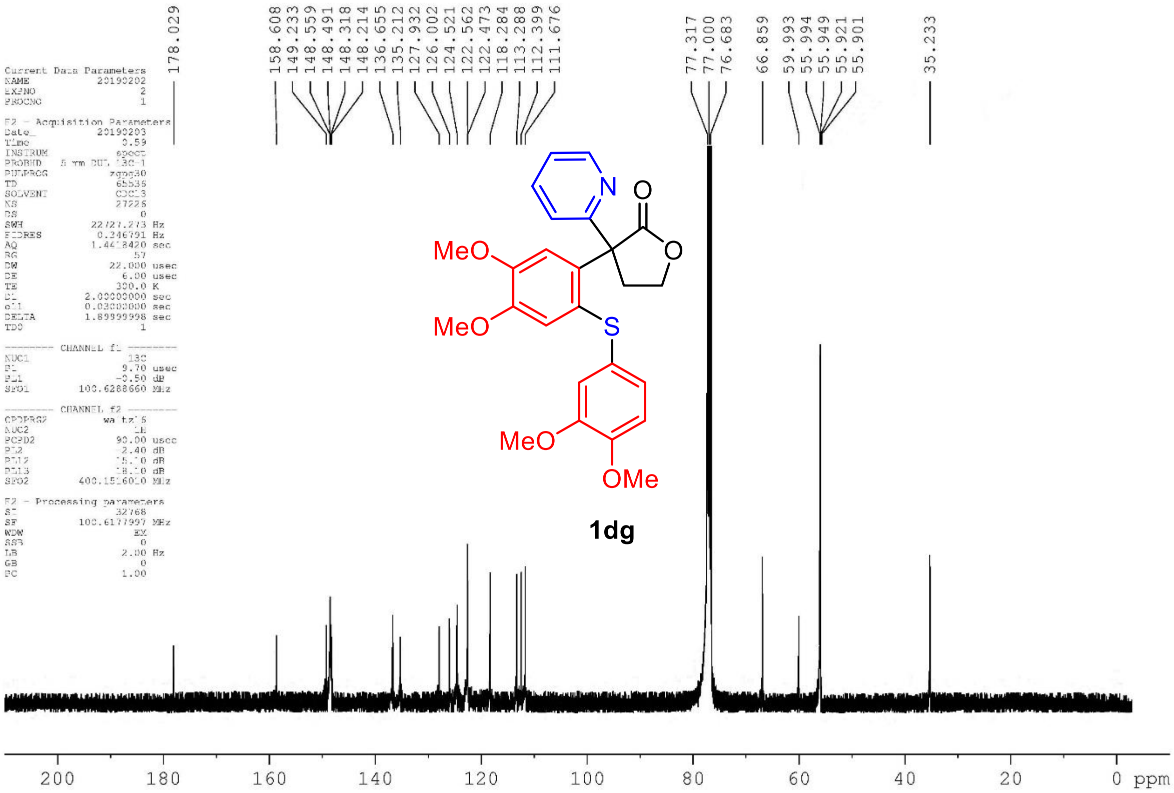

$^{13}C\{^{1}H\}$ NMR (100 MHz, $CDCl_3$) spectrum of compound **1dg**

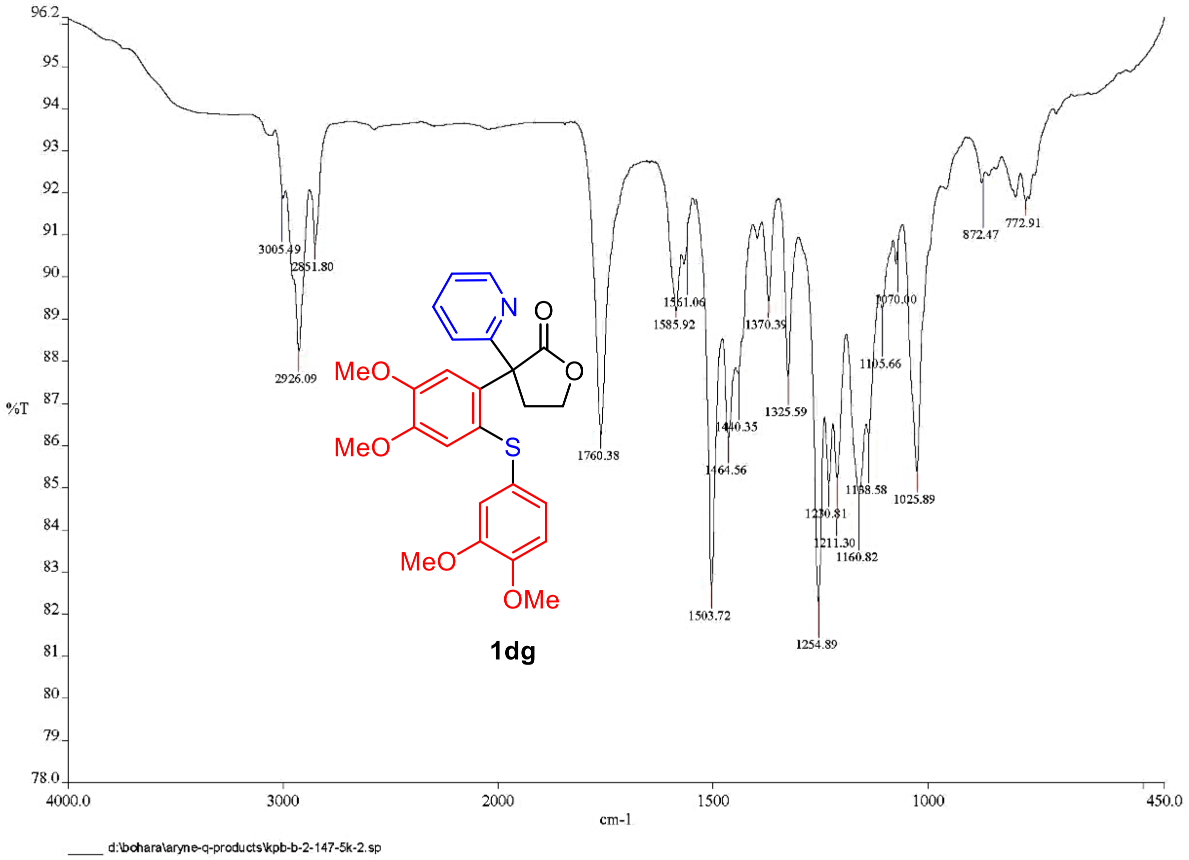

IR spectrum of compound **1dg**

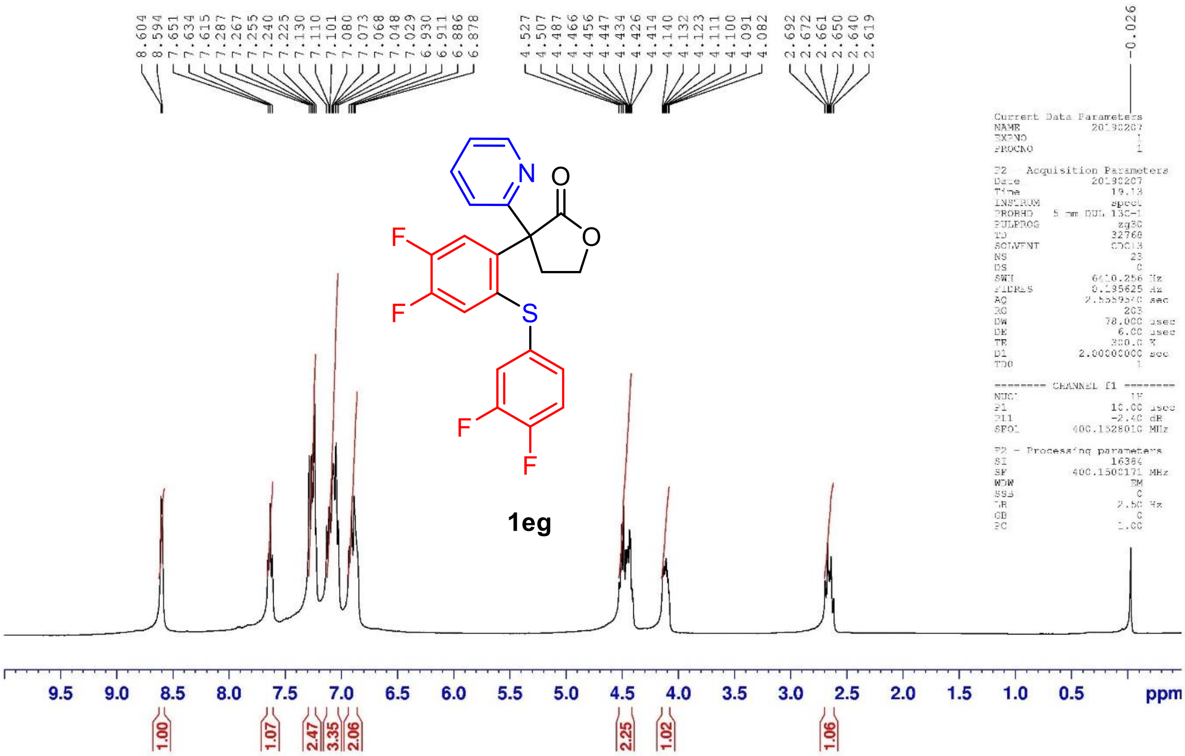

^{1}H NMR (400 MHz, $CDCl_3$) spectrum of compound **1eg**

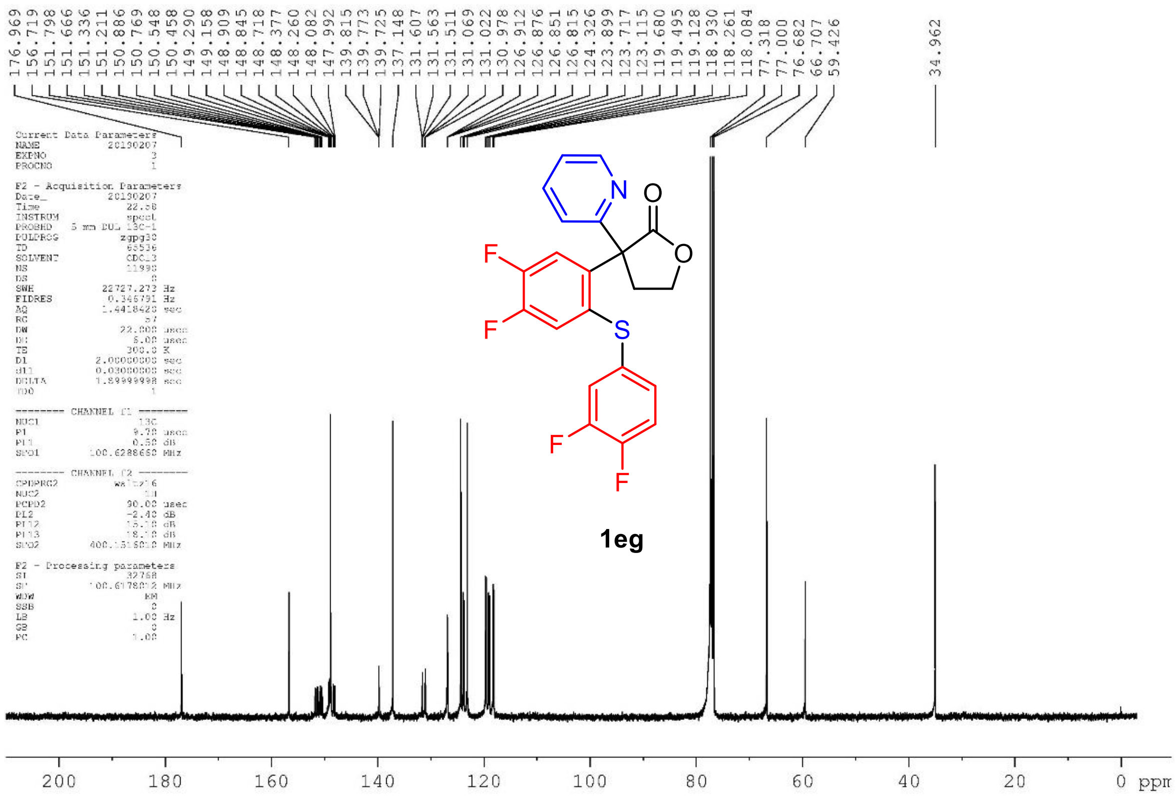

$^{13}C\{^1H\}$ NMR (100 MHz, $CDCl_3$) spectrum of compound **1eg**

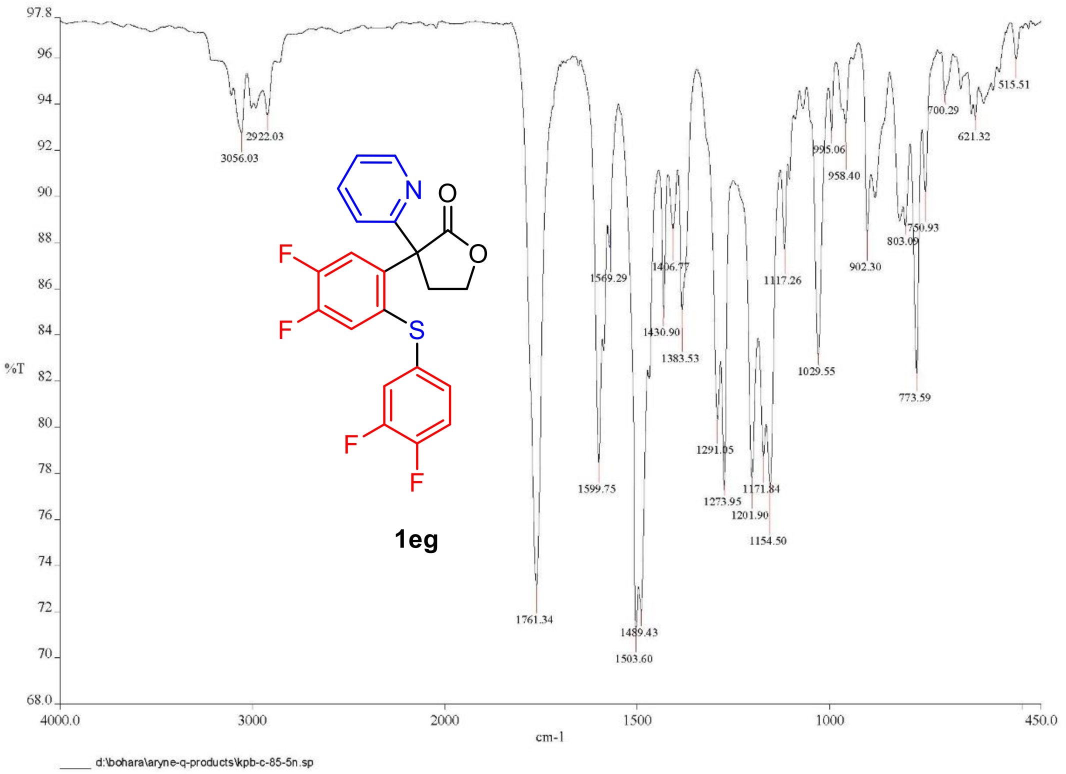

IR spectrum of compound **1eg**

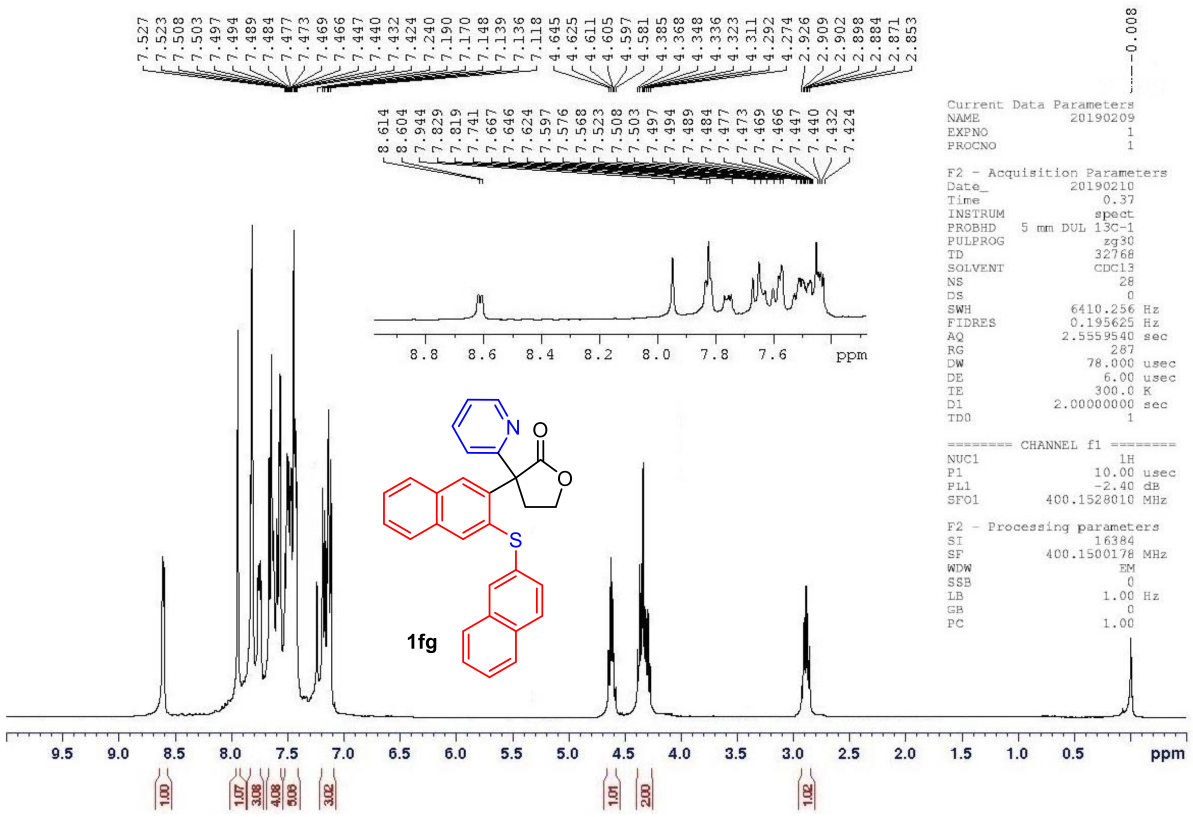

^{1}H NMR (400 MHz, $CDCl_3$) spectrum of compound **1fg**

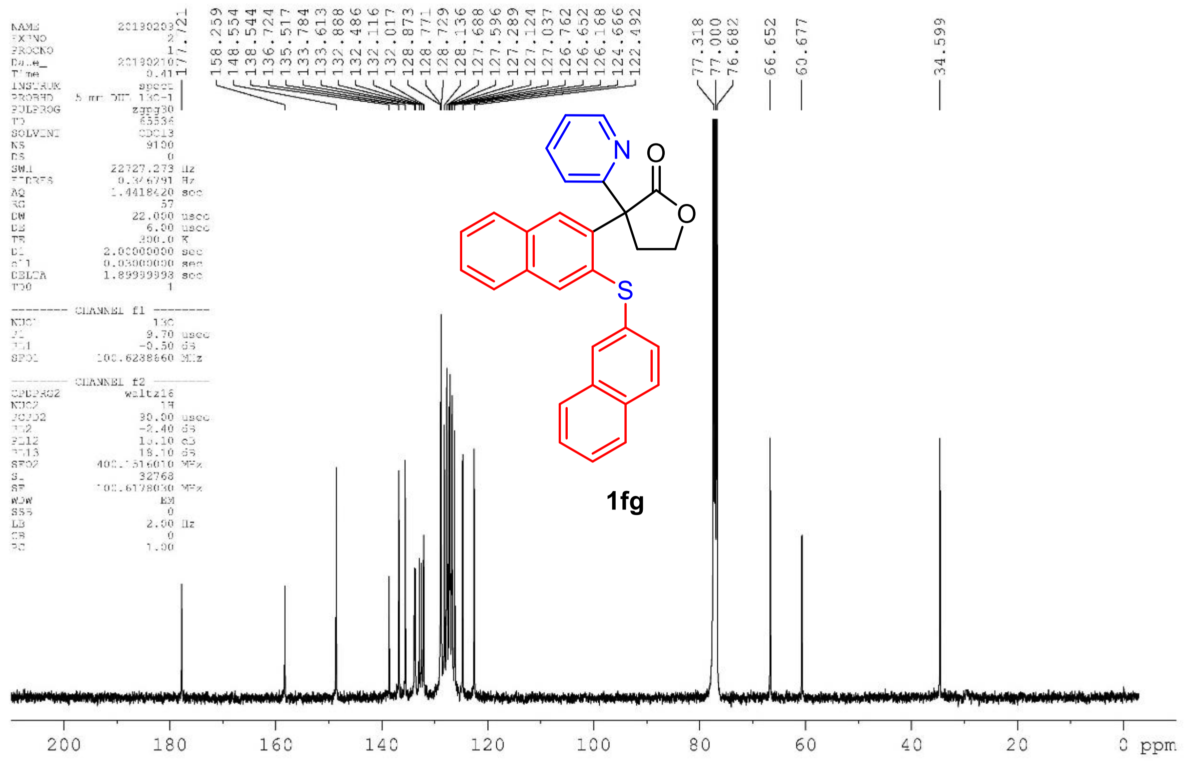

^{13}C{^{1}H} NMR (100 MHz, $CDCl_3$) spectrum of compound **1fg**

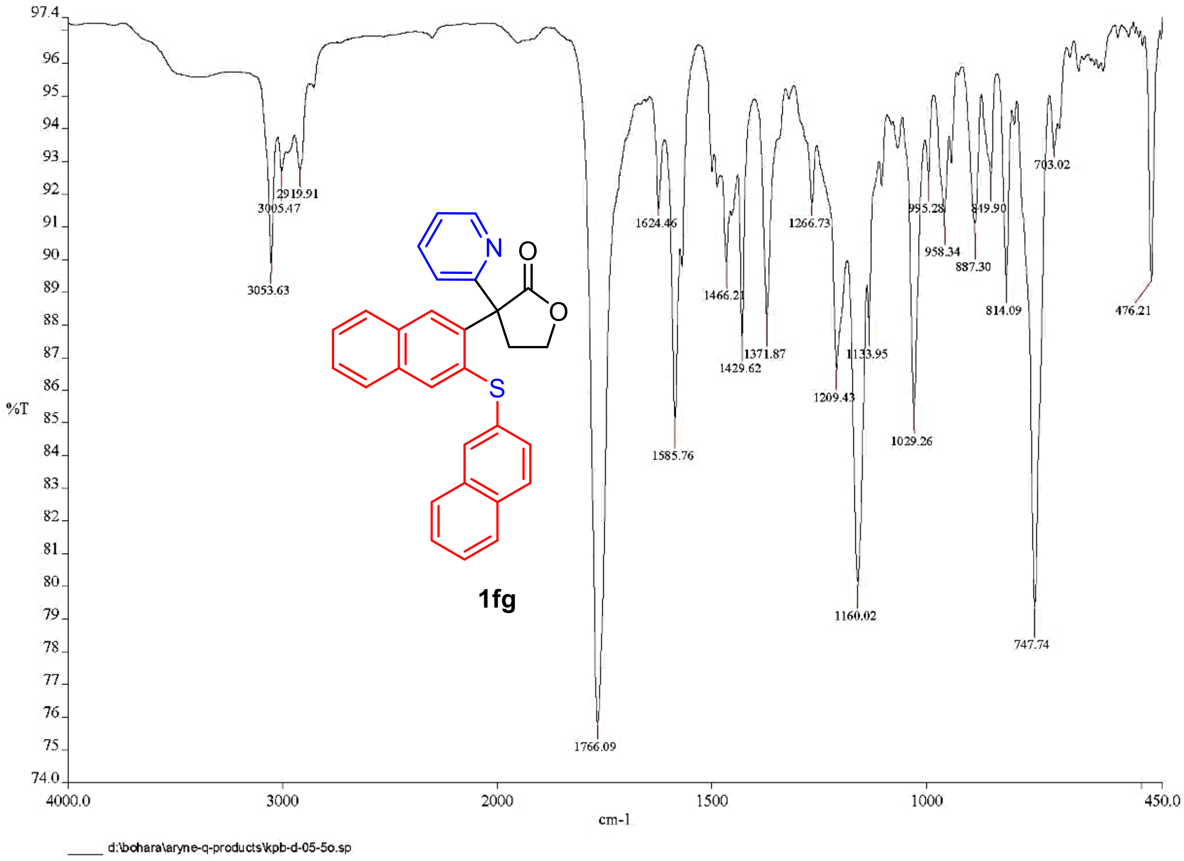

IR spectrum of compound **1fg**

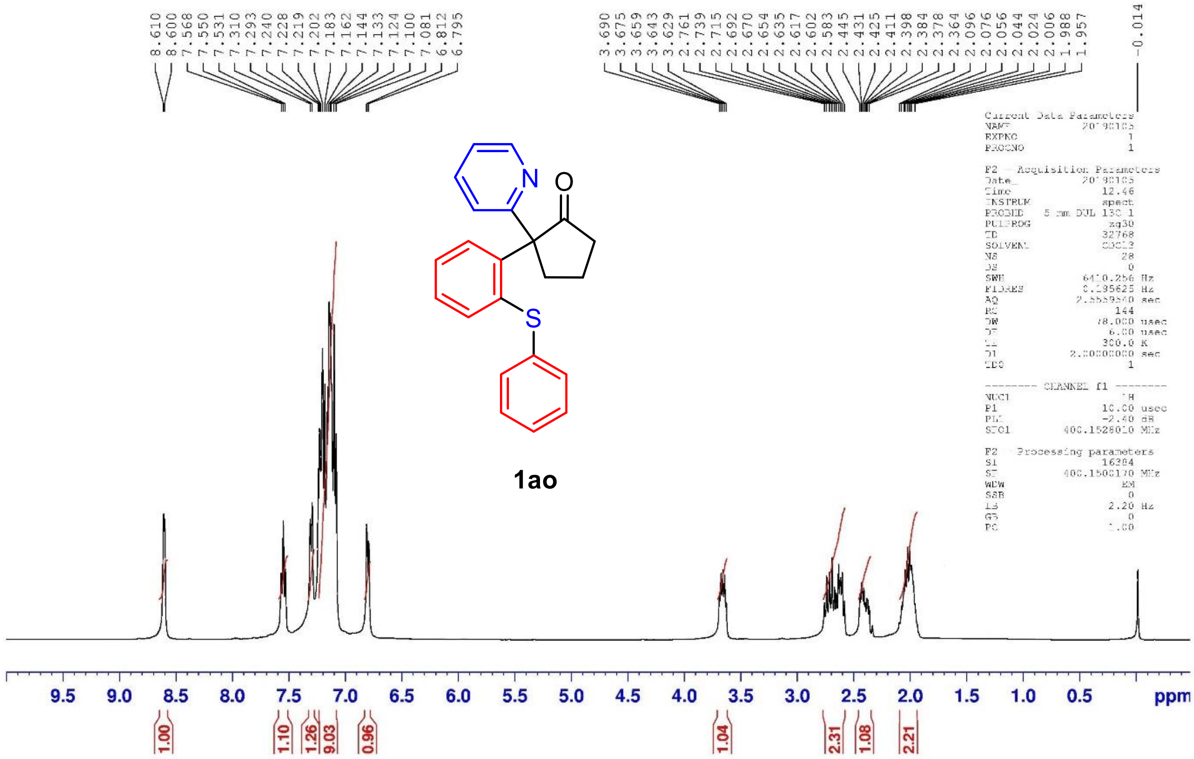

^{1}H NMR (400 MHz, $CDCl_3$) spectrum of compound **1ao**

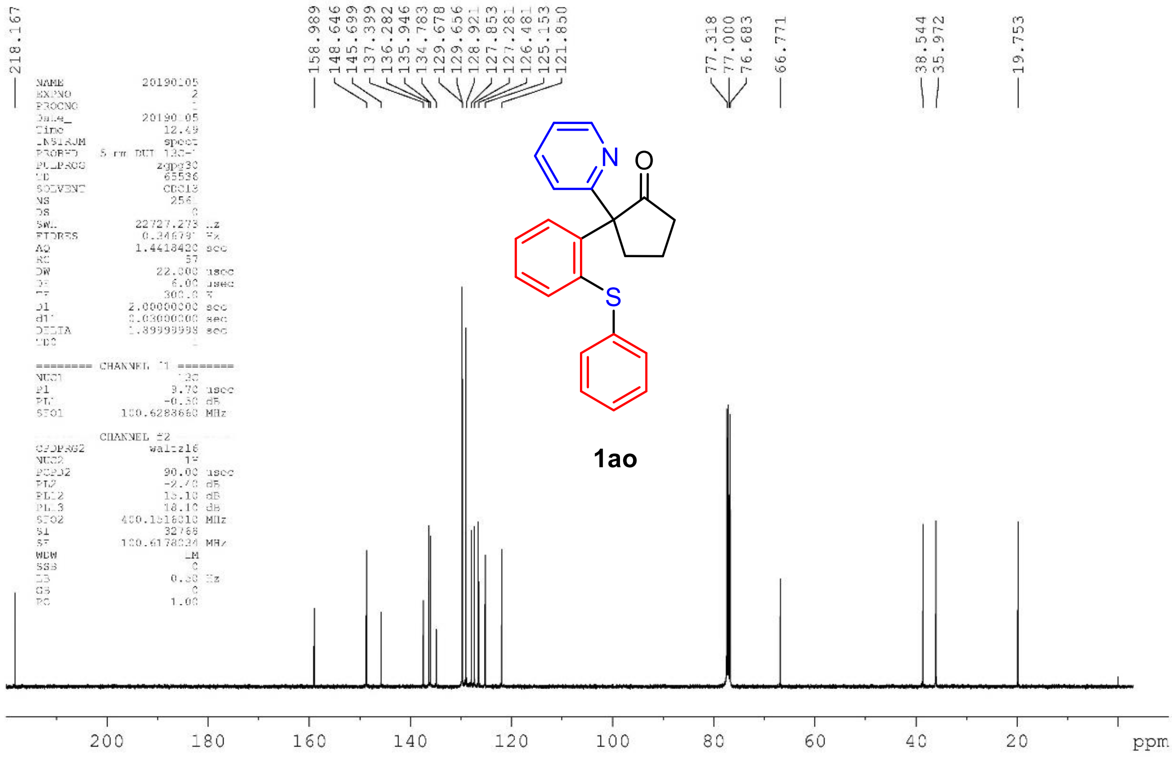

$^{13}C\{^{1}H\}$ NMR (100 MHz, $CDCl_3$) spectrum of compound **1ao**

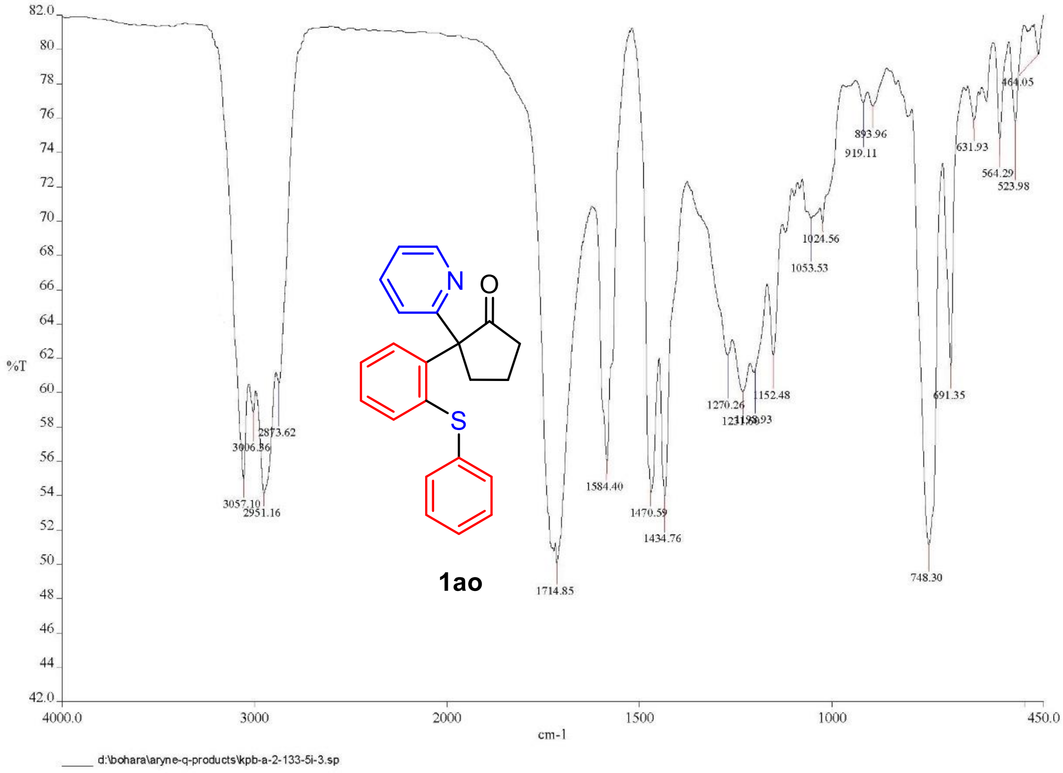

IR spectrum of compound **1ao**

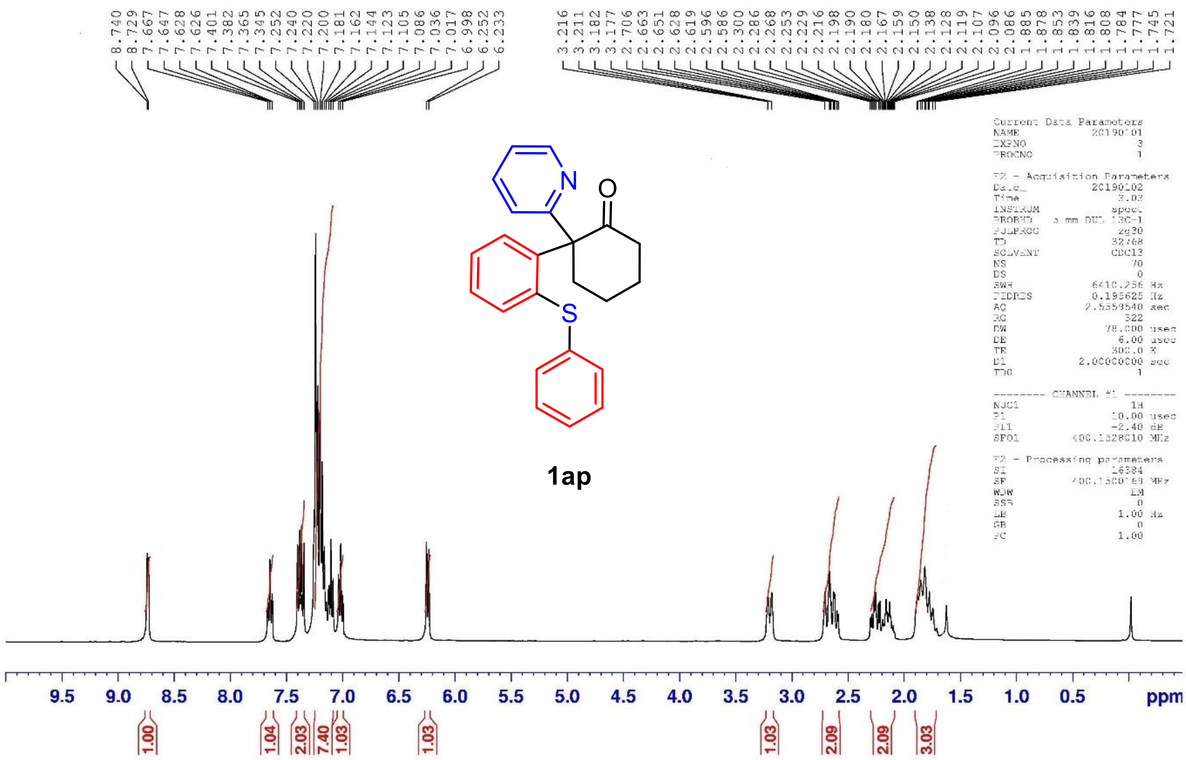

^{1}H NMR (400 MHz, $CDCl_3$) spectrum of compound **1ap**

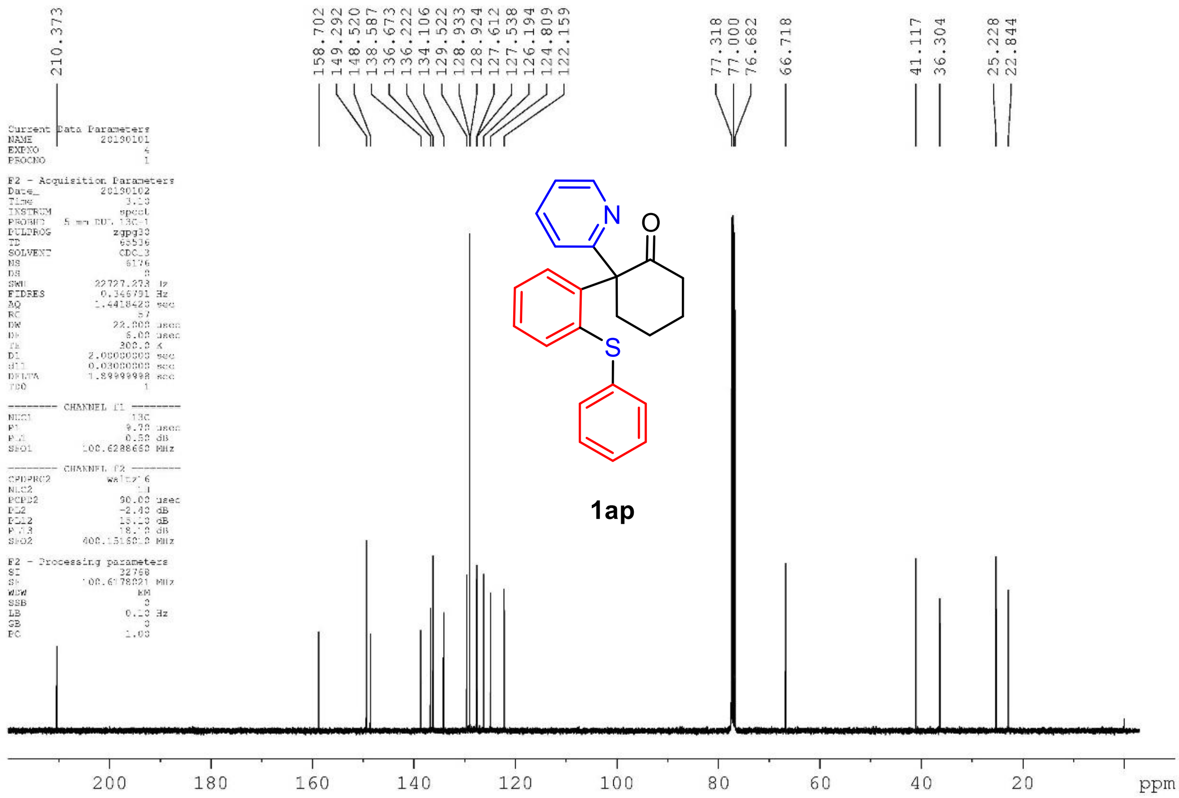

$^{13}C\{^{1}H\}$ NMR (100 MHz, $CDCl_3$) spectrum of compound **1ap**

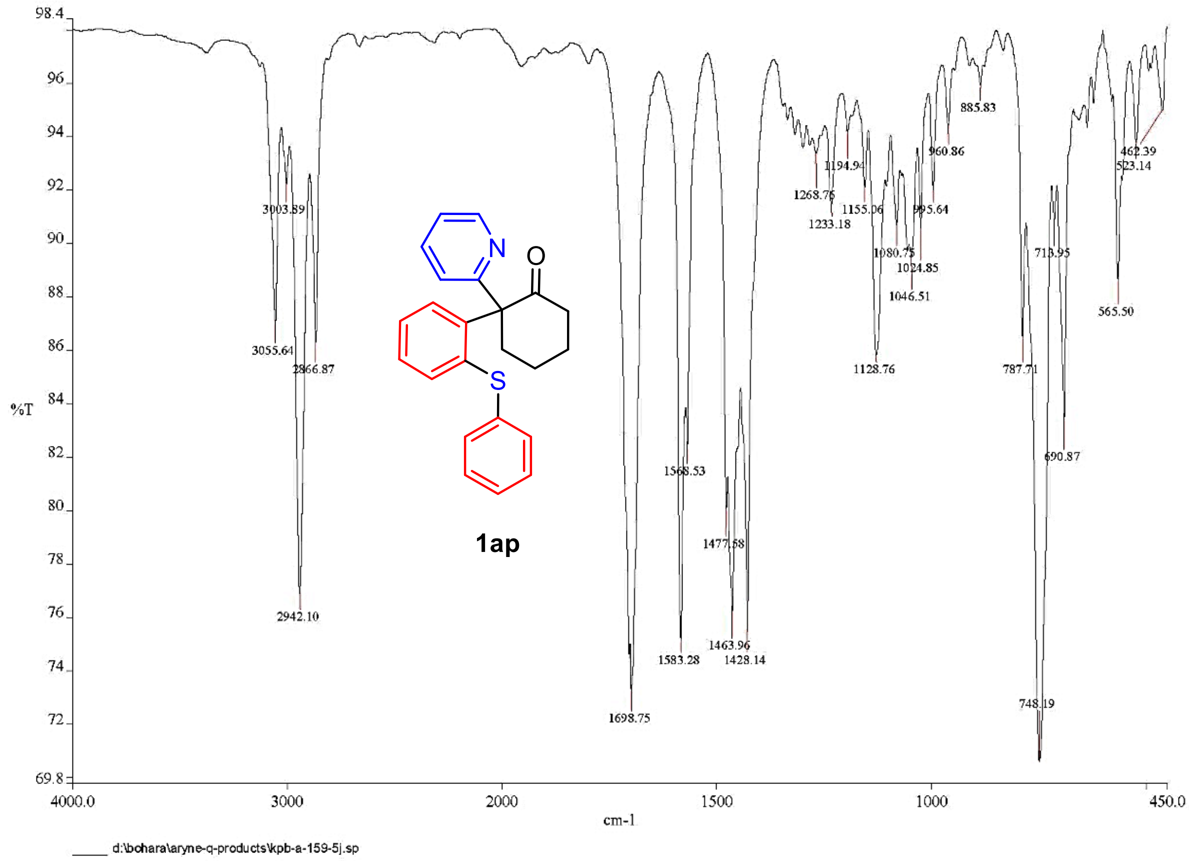

IR spectrum of compound **1ap**
